# Complex hybridization patterns in European pond turtles (*Emys orbicularis*) in the Pyrenean Region

**DOI:** 10.1038/s41598-018-34178-0

**Published:** 2018-10-29

**Authors:** Julia Pöschel, Botond Heltai, Eva Graciá, Marc Franch Quintana, Guillermo Velo-Antón, Oscar Arribas, Aitor Valdeón, Michael Wink, Uwe Fritz, Melita Vamberger

**Affiliations:** 1 Museum of Zoology (Museum für Tierkunde), Senckenberg Dresden, A. B. Meyer Building, 01109 Dresden, Germany; 2 Agricultural Biotechnology Institute – National Agricultural Research and Innovation Center, Szent-Györgyi Albert u. 4, 2100 Gödöllő, Hungary; 3 Ecology Area, Department of Applied Biology, Miguel Hernández University, Av. de la Universidad, Torreblanca, 03202 Elche, Spain; 4 CICGE – Centro de Investigação em Ciências Geo-Espaciais, Universidade de Porto, Observatório Astronómico Prof. Manuel de Barros Alameda do Monte da Virgem, 4430-146 Vila Nova de Gaia, Portugal; 5 CIBIO/InBIO, Centro de Investigação em Biodiversidade e Recursos Genéticos da Universidade do Porto, Instituto de Ciências Agrárias de Vairão, R. Padre Armando Quintas 7, 4485-661 Vairão, Portugal; 6 Avinguda Francesc Cambó 83, 08003 Barcelona, Spain; 7 Department of Geography and Regional Planning, University of Zaragoza, Pedro Cerbuna 12, 50009 Zaragoza, Spain; 8 Aranzadi Society of Sciences, Zorroagagaina, 11, 20014 Donostia-San Sebastian, Spain; 9 Heidelberg University, Institute of Pharmacy and Molecular Biotechnology, Im Neuenheimer Feld 364, 69120 Heidelberg, Germany

## Abstract

Hybrid zones are natural laboratories allowing insights in genetic processes like lineage diversification, speciation and introgression. Using large sampling, 15 microsatellite loci and a mitochondrial marker, we examined the Pyrenean contact zone of three pond turtle taxa (*Emys orbicularis orbicularis*, *E*. *o*. *galloitalica*, *E*. *o*. *occidentalis*). The Pyrenees are a biogeographically important region separating many lineages endemic to the Iberian Peninsula from their Western European counterparts. We discovered limited admixture, reflecting a complex biogeographic scenario. Simulations using Approximate Bayesian Computing supported that *E*. *o*. *orbicularis* invaded the Iberian Peninsula in the Holocene, circumventing the Pyrenees along the Mediterranean coast, and hybridized in the northern peninsula with the local coastal subspecies *galloitalica*, and to a lesser extent, with *occidentalis*. While *E*. *o*. *occidentalis*, and in particular *E*. *o*. *orbicularis*, expanded their ranges considerably during Holocene warming, *E*. *o*. *galloitalica* remained largely confined to its former Iberian refuge. Admixture among the three taxa is surprisingly low, and a future taxonomic investigation that includes the unstudied subspecies of *E*. *orbicularis* from North Africa, Eastern Europe and Asia has to determine whether their current status properly reflects their evolutionary divergence or whether certain taxa should be regarded as full species.

## Introduction

Hybridization between closely related taxa is widespread and plays an important evolutionary role because introgression may lead to genetic enrichment^1,2^. Introgression is defined as the introduction of foreign genetic material into the genome^1^, which happens in geographic regions where genetically distinct populations meet, mate and hybridize^3^. Such geographic contact zones correspond to two basic types^3^. Primary hybrid zones are understood as continuous populations diverging gradually along geographic clines, i.e. divergence is caused by isolation-by-distance with genetic divergence increasing with geographic distance. In contrast, secondary hybrid zones represent contact zones of genetically distinct populations that diverged in allopatry and came into contact again due to range shifts^3,4^. In the Western Palearctic, climatic changes during the Pleistocene triggered such processes^5,6^. During this period many thermophilic species retreated to southern glacial refugia^5–7^, e.g. common toads^8^, sand and green lizards^9,10^, pond turtles^11–13^ and grass snakes^14^, and recolonized more northerly regions with Holocene warming. Many refuge areas were located on the southern European peninsulas^5–7^, and this is particularly relevant for the Iberian Peninsula, which is separated from Western Europe by the Pyrenees. This mountain chain constitutes an important dispersal barrier and contact zone of Iberian and Western European lineages^5–7,15,16^. The Iberian Peninsula itself harbored several microrefugia or ‘refugia-within-refugia’^17^, in which distinct genetic lineages survived the last glacial. Holocene range expansions led to secondary contact and hybridization, for instance in tree frogs (*Hyla* spp.)^18^, ocellated lizards (*Timon lepidus* complex)^19^, Iberian wall lizards (*Podarcis bocagei* and *P*. *carbonelli*)^20^ and the Iberian hare and mountain hare (*Lepus granatensis*, *L*. *timidus*)^2,21^. Another example is the European pond turtle (*Emys orbicularis*).

**Figure 1:**
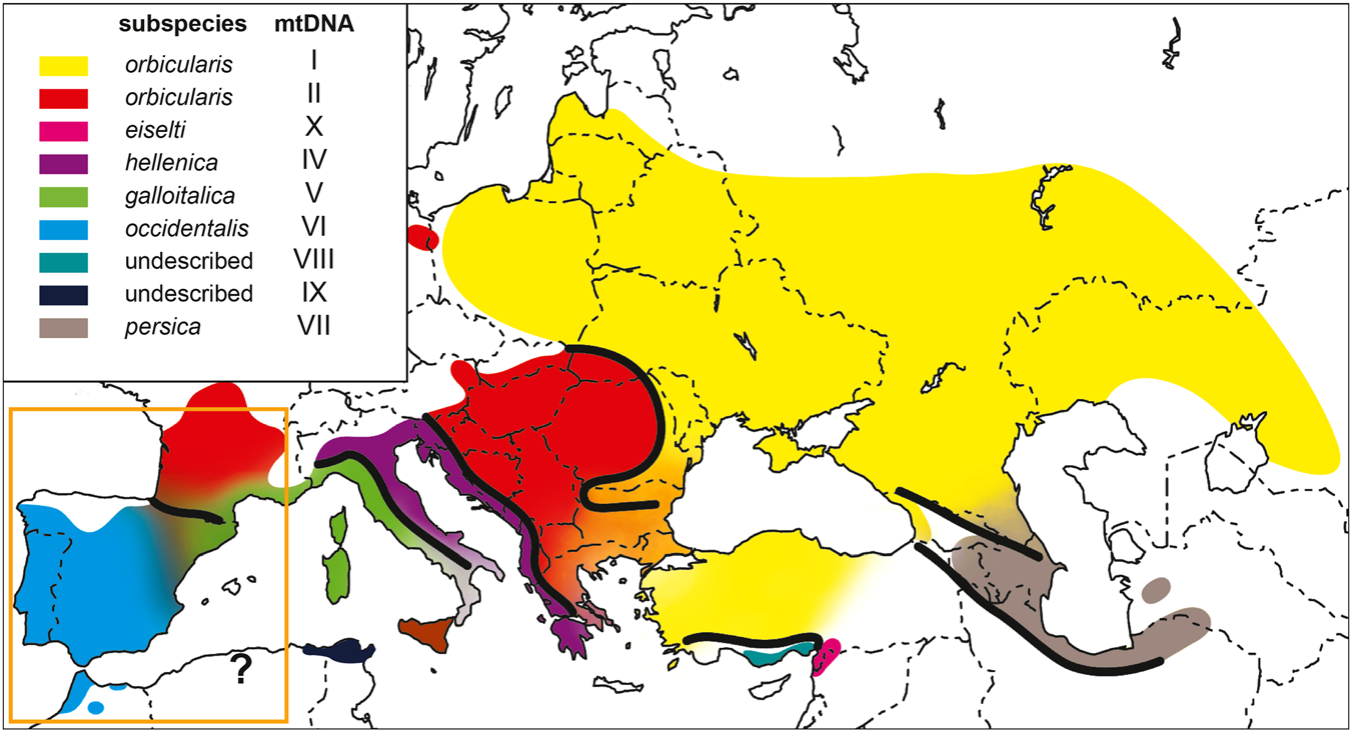
Distribution of *Emys orbicularis* and *E*. *trinacris* (brown). Different taxa correspond to different colors. The morphologically different subspecies of *E*. *orbicularis* correspond to distinct mtDNA lineages as shown in the inset. Merging colors indicate hybrid zones. Orange box shows the study region. Question mark represents an isolated population of unknown genetic identity. Map modified from Sommer *et al*.^31^. Map was created using ADOBE ILLUSTRATOR CS6 (http://www.adobe.com/products/illustrator.html).

The latter species has a wide distribution range, from northwestern Africa across the Iberian Peninsula and most of Europe and Turkey to the former Aral Sea in Central Asia^22^. Over this range, six named and two unnamed subspecies occur (Fig. 1), which differ morphologically and genetically. Except for *E*. *o*. *orbicularis*, each subspecies is characterized by a unique mitochondrial lineage and corresponds, using nuclear microsatellite loci, to at least one distinct nuclear genomic cluster^12,13,23,24^. The wide-ranging subspecies *E*. *o*. *orbicularis*, distributed from the Atlantic coast of France to Central Asia^22^, harbors two distinct mitochondrial lineages, which are less divergent than the other lineages^11,12,22^.

The distribution ranges of three subspecies (*Emys orbicularis orbicularis*, *E*. *o*. *galloitalica*, and *E*. *o*. *occidentalis*)^12,24^ meet in northern Spain. *Emys orbicularis occidentalis*, having an Ibero-Maghrebian range^25–28^, and *E*. *o*. *galloitalica*, distributed along the Mediterranean coast from northeastern Spain to Italy^13,24^, are thought to be old faunal elements of the Iberian Peninsula. Yet, the northern Iberian populations of *E*. *o*. *occidentalis* are derived from a recent range expansion from the south of the peninsula^26,28^. *Emys o*. *orbicularis* is considered a Holocene invader from Central and Southeastern Europe^11,12,29–31^. A pioneering study examining gene flow across the Iberian contact zone, using eight microsatellite loci and the mitochondrial cytochrome *b* (cyt *b*) gene of 146 samples from this region, found pronounced cytonuclear discordance, with mitochondrial introgression, but surprisingly low levels of nuclear admixture^24^. Because this result is unexpected for subspecies or recently diverged lineages, it required further examination. Here we use sequences of the mitochondrial cyt *b* gene and 15 highly polymorphic nuclear microsatellite loci of over 800 pond turtles, including 663 samples from the contact zone, to scrutinize the geographic and genetic extent of genetic admixture and introgression in the Iberian contact zone.

## Materials and Methods

### Sampling

**Figure 2:**
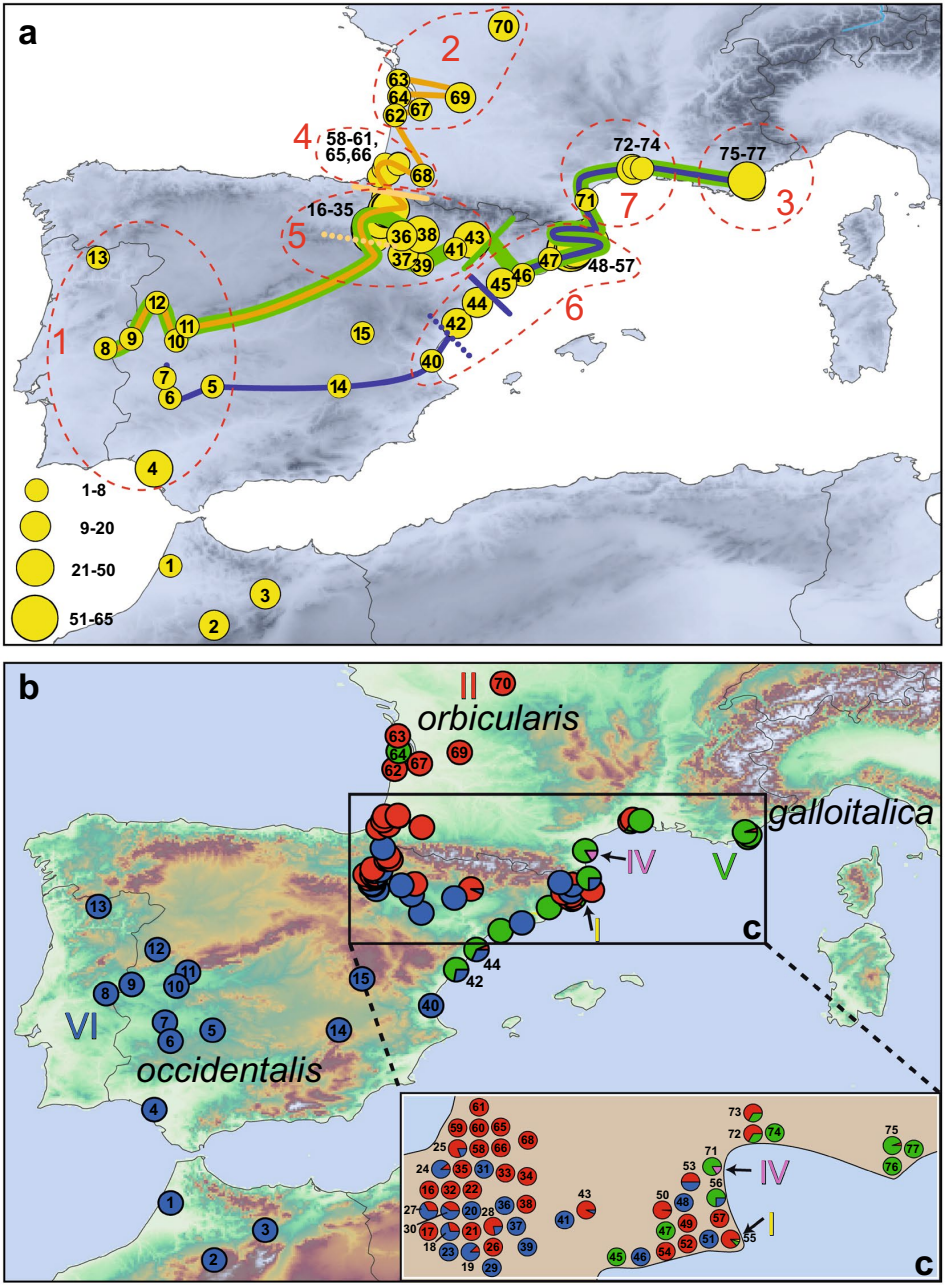
(**a**) Sampling sites and transects for cline analyses. Individual collection sites are consecutively numbered; adjacent sites, combined (for details, see Supporting Table S1). Symbol size corresponds to sample size. Orange line connects sites for cline analysis of Atlantic transect starting with population 8. Blue line connects sites for cline analysis of Mediterranean transect starting with population 7. Green line connects sites for cline analysis combining the Atlantic and Mediterranean transects over the Ebro valley, starting with population 8. Solidly colored bars indicate cline centers for microsatellites; dotted bars, cline centers for mtDNA. Sites 6 and 10 were excluded for cline analysis of microsatellite data because no data were available. Dashed red outlines (1–7) represent pooled groups for diversity and divergence analyses summarized in Table 1. (**b**) Distribution of mitochondrial lineages (I = yellow, II = red, IV = magenta, V = green, VI = blue, see Fig. S1). Colored circle segments represent proportions of mitochondrial lineages in respective population. Arrows highlight non-native lineages (I and IV). **(c)** Detail of map; height relief not shown for clarity. Individual sampling sites arbitrarily spread to show percentages; for original locations see major map and Table S1. Map was created using ARCGIS 10.2 (http://www.esri.com/arcgis) and ADOBE ILLUSTRATOR CS6 (http://www.adobe.com/products/illustrator.html).

We collected tissue, saliva or blood samples of 827 *Emys orbicularis* from 77 individual sampling sites in France, Spain, Portugal and Morocco (Fig. 2a; Table S1). No turtles were sacrificed for the present study. All sampling and methods were carried out in accordance with relevant guidelines, regulations and best ethical and experimental practice of the Senckenberg Nature Research Society. In addition to the 744 samples processed for this study, we included data for 26 samples from Morocco and 57 samples from France from previous studies^13,25^, representing together with the new samples the whole distribution range of *E*. *o*. *occidentalis* and the adjacent parts of the ranges of *E*. *o*. *galloitalica* and *E*. *o*. *orbicularis*, including the previously identified secondary contact zones of these taxa^11,32–34^. The taxonomic identity of the samples was determined according to the accepted distribution range of the taxa and their previously identified contact zones^22,32–34^. Samples were preserved in ethanol or EDTA buffer^35^ and stored at −80 °C in the tissue sample collection of the Museum of Zoology, Senckenberg Dresden, until processing.

### DNA extraction, PCR and sequencing

We isolated total DNA using standard proteinase K and phenol chloroform protocols^36^, the DTAB method^37^ or the Innu-Prep DNA Mini Kit (Analytik Jena GmbH, Jena, Germany). Laboratory procedures for cyt *b* gene amplification and sequencing followed an established protocol^13^.

If sampling sites were represented by more than twenty individuals, we randomly selected for mtDNA sequencing twenty samples. In total, we generated 564 new cyt *b* sequences that were merged with 101 previously published sequences^11–13,24,25,27,32,38–40^ using BIOEDIT 7.0.5.2^41^. New sequences were trimmed to 1031 bp length to match previously published data. Using POPART (http://popart.otago.ac.nz) and the implemented parsimony network algorithm of TCS^42^, we built a haplotype network using these data. Haplotype nomenclature follows previous studies^11–13,24,25,27,32,38–40,43^ in that a letter follows a Roman numeral, corresponding to the individual haplotype and the mitochondrial lineage, respectively. The European Nucleotide Archive (ENA) accession number of a newly identified haplotypes is LS997573.

In addition to the cyt *b* gene, we genotyped 744 samples using 15 previously characterized unlinked and highly polymorphic microsatellite loci^33,44^ according to procedures described elsewhere^25^ (Table S2). Eighty-three additional samples were genotyped in previous studies^13,25^ and added to our data set (Table S1). We determined microsatellite lengths on an ABI 3130xl Genetic Analyzer (Applied Biosystems) using the GeneScan 600 LIZ Size Standard (Applied Biosystems) and PEAK SCANNER 1.0 (Life Technologies, Carlsbad, CA, USA).

### Population genetic analysis

We revealed the presence of null alleles using MICRO-CHECKER 2.2.3^45^ and corrected the data set accordingly for null alleles^46^. Then, we analyzed microsatellite data with the unsupervised Bayesian clustering approach of STRUCTURE 2.3.4^46–48^ using the admixture model and correlated allele frequencies. We ran STRUCTURE with *K* values up to 15 and determined the optimal number of clusters using the Δ*K* method^49^ in STRUCTURE HARVESTER^50^. We repeated calculations 10 times for each *K* using a burn-in of 250,000 generations and set the number of further MCMC runs to 750,000 generations for each run. We visualized clustering results and individual admixture as bar plots using DISTRUCT 1.1^51^.

Because uneven sample sizes may bias STRUCTURE results^52^, we reduced local samples representing 25 or more individuals by randomly selecting 20 individuals. Based on the remaining 732 samples, we performed STRUCTURE analyses for this data set and for two subsets, corresponding to turtles from north (133 samples) and south of the Pyrenees (599 samples), respectively.

In addition to STRUCTURE analyses, we examined our microsatellite data with Principal Component Analyses (PCAs) using the package ADEGENET^53^ for R 3.2.3.

### Determination of admixture threshold

We used simulations with the software HYBRIDLAB 1.0^54^ for determining the threshold for mixed ancestry in STRUCTURE analyses. One data set included 20 genotypes each of pure *E*. *o*. *orbicularis* and *E*. *o*. *galloitalica* as parental genotypes, another data set included 20 genotypes each of pure *E*. *o*. *galloitalica* and pure *E*. *o*. *occidentalis* as parental genotypes, and the third data set included 20 genotypes each of pure *E*. *o*. *orbicularis* and *E*. *o*. *occidentalis* as parental genotypes. Based on each data set, we modeled 20 genotypes of each hybrid class (F_1_, F_2_ and two backcrosses). We then subjected the simulated hybrid data to STRUCTURE analyses, together with the data of the 20 pure genotypes of each taxon. According to these STRUCTURE runs including modeled hybrid genotypes, we identified individuals with cluster membership proportions below 85% as having mixed ancestries.

### Diversity within and divergence among pure and hybrid populations

We assigned our 71 individual collection sites to seven geographically coherent groups to achieve comparable sample sizes. For each individual subspecies, we lumped together collection sites representing pure populations (groups 1–3). According to the collection sites, the four remaining groups 4–7 corresponded to putatively admixed populations. Groups 4 and 5 represented populations in the west of the contact zone (Fig. 2a); groups 6 and 7, populations in the east of the contact zone (Fig. 2a).

Group 1 consisted exclusively of pure *E*. *o*. *occidentalis* (Fig. 2a: sites 4–13); group 2, of pure *E*. *o*. *orbicularis* (Fig. 2a: sites 62, 63, 67, 69, 70); and group 3, of pure *E*. *o*. *galloitalica* (Fig. 2a: sites 75–77). Group 4 was comprised of putatively admixed populations north of the Pyrenees (Fig. 2a: sites 58–61, 65, 66, 68); group 5, of putatively admixed populations southwest of the Pyrenees (Fig. 2a: sites 16–39, 41, 43); group 6, of putatively admixed populations from the Spanish Mediterranean coast (Fig. 2a: sites 42, 44–57); and group 7, of putatively admixed populations from the French Mediterranean coast (Fig. 2a: sites 71–74). For each group, diversity and divergence parameters were calculated for microsatellite data and mtDNA using two different approaches. Firstly, groups 1 (*E*. *o*. *occidentalis*), 2 (*E*. *o*. *orbicularis*) and the admixed groups 4 and 5 were compared. Then groups 1 (*E*. *o*. *occidentalis*), 2 (*E*. *o*. *orbicularis*), 3 (*E*. *o*. *galloitalica*), and the admixed groups 6 and 7 were compared. We calculated for the two approaches population genetic diversity indices, pairwise *F*_ST_ values and analyses of molecular variance (AMOVAs) using CONVERT 1.31^55^ and ARLEQUIN 3.5.1.2^56^.

### Cline analyses

To examine nuclear genomic and mitochondrial introgression patterns across the Pyrenean contact zone, we computed hybrid clines for three transects (Fig. 2a). An Atlantic transect starts inland in the Iberian Peninsula and runs along the Atlantic coastline from Spain to France, i.e. it runs from pure populations of *E*. *o*. *occidentalis* through the contact zone to pure populations of *E*. *o*. *orbicularis*. A Mediterranean transect starts also inland in the Iberian Peninsula and runs across the contact zone of the three taxa at the Mediterranean coast to southeast France, i.e. the transect runs from pure populations of *E*. *o*. *occidentalis* to pure populations of *E*. *o*. *galloitalica*. The third transect connects the Atlantic and Mediterranean transects along the course of the Ebro valley. For analyzing the third transect, we used only microsatellite data to calculate the cline, because the wide syntopic occurrence of distinct mitochondrial lineages along the transect prevents the use of this marker system.

For the cline analyses of microsatellite data, we calculated the mean proportion *Q* of cluster membership (as inferred by STRUCTURE) for each population^13,19^. For mtDNA data, we used the frequency of haplotypes of each mitochondrial lineage per population, that is the frequency of haplotypes of *E*. *o*. *orbicularis* (lineage II), *E*. *o*. *galloitalica* (lineage V) or *E*. *o*. *occidentalis* (lineage VI).

We ran each of the three independent cline analyses in HZAR^57^, corresponding to each transect. This R package fits molecular genetic data to classic equilibrium cline models using the Metropolis-Hastings Markov chain Monte Carlo algorithm. For cline fitting, we added geographical information of sites to genetic information. For this, we measured geographical distances between populations using ARCGIS 10.3 (www.esri.com/arcgis) and adjusted then the 15 HZAR models to the mean proportions of cluster membership or haplotype frequencies (Table S3). We set a burn-in of 10,000 iterations followed by additional 90,000 iterations and determined the best cline model using AIC scores (Table S3).

### Demographic analyses using DIYABC

**Figure 3:**
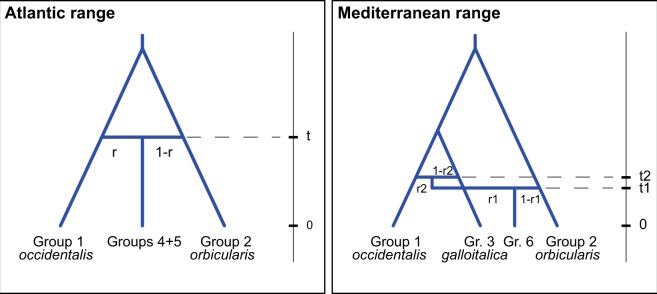
Phylogeographic scenarios tested using DIYABC. Numbers refer to groups of populations shown in Fig. 2a: group 1—pure *Emys orbicularis occidentalis*; group 2—pure *E*. *o*. *orbicularis*; group 3—pure *E*. *o*. *galloitalica*; groups 4 and 5—contact *E*. *o*. *occidentalis* x *E*. *o*. *orbicularis*; group 6—contact *E*. *o*. *occidentalis* x *E*. *o*. *galloitalica* x *E*. *o*. *orbicularis*. Times and admixture rates not to scale.

For dating and inferring the history of the secondary contact between the pond turtle taxa, we analyzed both microsatellite and mtDNA data using the Approximate Bayesian Computation approach of DIYABC 2.1.0^58^. This software allows for testing demographic scenarios and estimating their demographic parameters. For doing so, we used the groups of Fig. 2a representing pure or admixed populations for inferring the contact between *E*. *o*. *occidentalis* and *E*. *o*. *orbicularis* (Atlantic range) and among *E*. *o*. *occidentalis*, *E*. *o*. *galloitalica* and *E*. *o*. *orbicularis* (Mediterranean range; Fig. 3). For both scenarios, we assumed constant effective population sizes and that pure populations originated from common ancestors, with an underlying branching pattern corresponding to the topology reported in Stuckas *et al*.^25^. For the Atlantic range, we assumed a single admixture event between *E*. *o*. *occidentalis* and *E*. *o*. *orbicularis t* time ago, with an admixture rate *r* (contribution of *E*. *o*. *occidentalis* to the admixed population). For the Mediterranean range, we assumed two different admixture events: First, contact between *E*. *o*. *occidentalis* and *E*. *o*. *galloitalica*, and subsequently contact with *E*. *o*. *orbicularis*. The latter subspecies is thought to be a Holocene invader whereas *E*. *o*. *galloitalica* survived the last glaciation along the Mediterranean coast^24^ and, therefore, contact with the northwards expanding *E*. *o*. *occidentalis*^26,28^ should have established first. From the present to the past, these contacts have times *t1* and *t2* and admixture rates *r1* (contribution of the admixed population *occidentalis* x *galloitalica*) and *r2* (contribution of *E*. *o*. *orbicularis*).

We set priors to follow uniform distributions ranging from 10 to 10,000 for effective population sizes and admixture times and for diversification times, from 10 to 100,000. We simulated 10^5^ data sets for each lineage to build a reference table and used the closest 1% of the simulated data sets to the observed data to estimate demographic parameters. As summary statistics we used for mtDNA data: (i) the number of haplotypes, (ii) the number of segregating sites, (iii) the mean of pairwise differences, (iv) private segregating sites, (v) the mean of pairwise differences (W), (vi) the mean of pairwise differences (B), and (vii) *F*_ST_; and for microsatellites: (viii) the mean number of alleles, (ix) the mean genetic diversity, and (x) *F*_ST_. We accepted the default mutation rate suggested for microsatellites in the software (minimum of 10^−4^ and a maximum of 10^−3^). For mtDNA, we considered a minimum mutation rate of 10^−9^ (as approximately calculated in Lenk *et al*.^11^) and two orders of magnitude more as maximum. All other settings were left as suggested in the DIYABC 2.0 handbook. To convert the obtained estimates of admixture and divergence times from generations to years, we assumed an approximate generation time of 15 years, based on life history data of a pond turtle population from southwestern Spain (sexual maturity approx. 6 years, maximum longevity 29 years^59^).

## Results

### Mitochondrial phylogeography

We found that most turtles had mitochondrial haplotypes of lineages II, V or VI (Fig. 2b), which corresponded to the subspecies *Emys orbicularis orbicularis*, *E*. *o*. *galloitalica*, and *E*. *o*. *occidentalis*, respectively^12,13,25^. This is in line with expectations from previous investigations^11–13,24,26,27^. In addition, we recorded a few turtles having haplotypes of lineages I or IV, which were not expected to occur in our study region and which are interpreted as introduced turtles (a common phenomenon in Europe^22,39^). The native distribution ranges of lineages I and IV are Eastern Europe and Western Asia as well as eastern Italy and the southwestern Balkans, respectively^12,13^. In parsimony network analyses (Fig. S1), haplotypes of lineages II and V differed by a minimum of 16 mutational steps; haplotypes of lineages II and VI, by a minimum of 15 mutational steps; and haplotypes of lineages V and VI, by a minimum of three mutational steps. Among the 15 haplotypes of lineage II, a maximum number of six mutational steps occurred; among the nine haplotypes of lineage V, three steps; and among the 16 haplotypes of lineage VI, ten steps.

Haplotypes typical for *E*. *o*. *occidentalis* (lineage VI) occurred in Morocco and the Iberian Peninsula (Fig. 2). Haplotypes typical for *E*. *o*. *orbicularis* (lineage II) were distributed in our study region along the Atlantic coast of France through northern Spain to the Mediterranean coast of Spain and France. Along the Mediterranean coast of Spain haplotypes of lineage II occurred together with haplotypes of *E*. *o*. *galloitalica* (lineage V) and *E*. *o*. *occidentalis* (lineage VI). Moreover, haplotypes of *E*. *o*. *orbicularis* occurred together with haplotypes of *E*. *o*. *occidentalis* south of the Pyrenees. Furthermore, we found haplotypes of *E*. *o*. *galloitalica* (lineage V) along the Mediterranean coast of France and in one turtle (Fig. 2b: site 64) from the Atlantic coast of France. We recorded the non-native haplotypes of lineages I and IV far away from their known distribution ranges (Fig. 2b: sites 55 and 71).

In lineage II (*E*. *o*. *orbicularis*), there was no obvious geographical pattern in haplotype distribution (Fig. S2). Almost all samples yielded haplotype IIa. Only two samples of the Département Pyrénées-Atlantiques (Fig. 2b; site 68 and Fig. S2) had haplotype IIh, which differed in one mutation step from IIa. Also, for lineage V (*E*. *o*. *galloitalica*), there was no obvious pattern. All turtles harbored haplotype Va. In contrast, in lineage VI (*E*. *o*. *occidentalis*) some haplotypes were clearly geographically differentiated. Three haplotypes (VIc, VIf, VIi) were endemic to northern Morocco and showed mutually exclusive distributions with Iberian haplotypes. In the Iberian Peninsula, haplotype VIa was widespread and the most frequent haplotype. We found it in some sites together with rare Iberian haplotypes of lineage VI. Some western sites (Fig. S2) in Spain harbored only haplotype VId, which differed in two mutation steps from VIa. Furthermore, we recorded some rare haplotypes in the west and east of Spain (VIb, VIg, VIp). The newly identified haplotype VIp differed in one mutational step from VIa. The North African haplotype VIc differed from VIa in one mutational step, and haplotype VIi in one step from VIc. Haplotype VIf differed in three, and haplotype VIn from Morocco and the Iberian Peninsula^27^, in seven mutation steps from VIa.

### Microsatellite analyses

**Figure 4:**
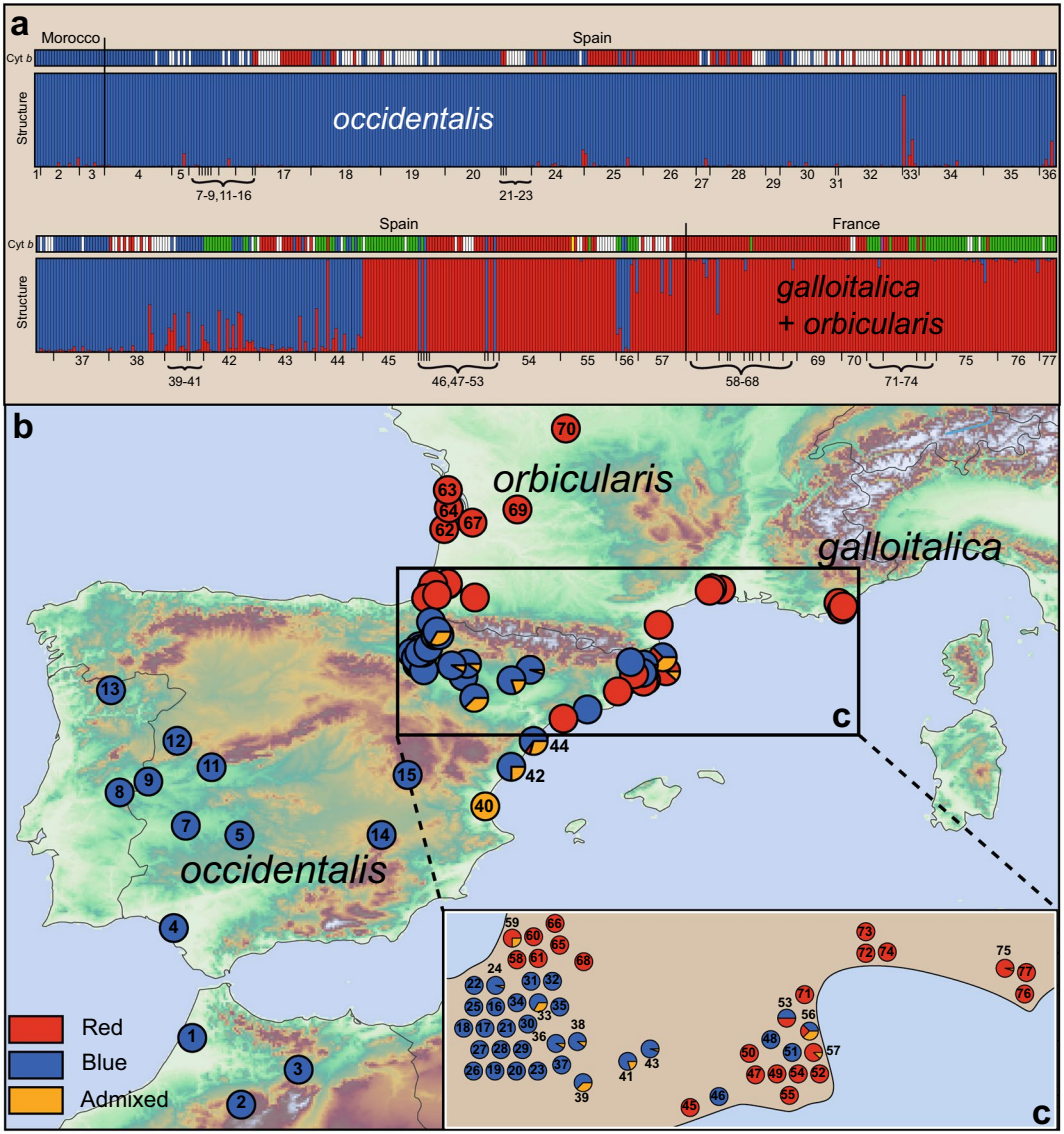
(**a**) Genotypic structuring of 732 pond turtles from 75 sites for *K* = 2 using 15 microsatellite loci. The STRUCTURE run with the best probability value is shown. Distinct clusters are color-coded; samples are arranged according to site numbers (Fig. 1). Within each cluster, an individual is represented by a vertical segment that reflects its ancestry. Mixed ancestries are indicated by differently colored sectors corresponding to inferred genetic percentages of the respective cluster. The mitochondrial lineage of each sample is shown above the STRUCTURE diagrams (red = lineage II, green = lineage V, blue = lineage VI, white = no data available). Colors of populations in the map **(b)** correspond to STRUCTURE clusters; slices represent turtles with mixed ancestries or conflicting cluster assignment (percentages). Detail of map **(c);** height relief not shown for clarity. Individual sampling sites arbitrarily spread to show percentages; for original locations see major map and Table S1. Map was created using ARCGIS 10.2 (http://www.esri.com/arcgis) and ADOBE ILLUSTRATOR CS6 (http://www.adobe.com/products/illustrator.html).

The 15 microsatellite loci were highly polymorphic. The number of alleles per locus varied between 12 and 29 (Table S2), and the total allele number was 293. According to our HYBRIDLAB calculations, individuals with *Q* values > 85% were to be identified as pure, while lower percentages corresponded to admixed turtles. For the analysis of the whole data set, the Δ*K* method^49^ supported *K* = 2 as optimal number of STRUCTURE clusters (Figs 4 and S3: blue and red clusters). The blue cluster corresponded to turtles from Morocco and the Iberian Peninsula. The red cluster was comprised of all turtles from France and many, but not all, turtles from the Spanish Mediterranean coast (Fig. 4). The geographical distribution of turtles assigned to the blue cluster matched well with the range of the haplotypes typical for *E*. *o*. *occidentalis*. However, with respect to mitochondrial haplotypes, pronounced cytonuclear discordance was obvious in northern and northeastern Spain (Ebro valley, Mediterranean coast) in that haplotypes typical for *E*. *o*. *galloitalica* (lineage V) and *E*. *o*. *orbicularis* (lineage II) were combined with nuclear genotypes of *E*. *o*. *occidentalis* (blue cluster). Weak nuclear admixture also occurred in these regions.

Since STRUCTURE is known to detect only the uppermost hierarchical level of genetic partitioning^49^, we ran STRUCTURE also for subsets from north and south of the Pyrenees, respectively. For both subsets, *K* = 2 was revealed as best solution (Figs S4 and S5). One of the two clusters from north of the Pyrenees corresponded to turtles distributed along the Atlantic coast of France and in central France (Fig. S6), i.e. with *E*. *o*. *orbicularis*^22,34^. All of these turtles, with one exception (haplotype Va), had mitochondrial haplotypes of lineage II, typical for *E*. *o*. *orbicularis* (Fig. 2). This ‘*orbicularis* cluster’ and another microsatellite cluster, corresponding to haplotypes typical for *E*. *o*. *galloitalica* (with mtDNA lineage V, typical for this subspecies), were represented by turtles occurring on the Mediterranean coast of France (Fig. S6). In the region of the Rhône mouth, where haplotypes of lineages II and V occur syntopically^34,43^ (Fig. 2), turtles were largely assigned to the ‘*orbicularis* cluster’, whereas the remaining populations from southern France mainly corresponded to the ‘*galloitalica* cluster’.

As regards the southern subset, one of the two clusters was distributed from Morocco over the entire Iberian Peninsula, matching well with the distribution range of haplotypes typical for *E*. *o*. *occidentalis* (Fig. S6). However, some populations from the Mediterranean coast of Spain were assigned to the second cluster, with cytonuclear discordance and nuclear admixture between both clusters along the Mediterranean coast and in the Ebro valley (Fig. S6).

**Figure 5:**
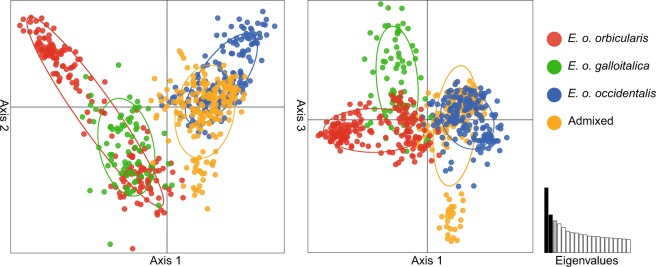
PCA using microsatellite data of 732 pond turtles representing the currently recognized *Emys* taxa of the Iberian Peninsula. The first, second and third principal components explain 11.44%, 6.69% and 5.62% of the variance. Oval outlines represent 95% confidence intervals; non-overlapping intervals denote significantly different clusters. The figure was created using R^57^ and ADOBE ILLUSTRATOR CS6 (http://www.adobe.com/products/illustrator.html).

The Principal Component Analyses (PCAs) corroborated these results (Fig. 5). The two subspecies *E*. *o*. *orbicularis* (red) and *E*. *o*. *galloitalica* (green) were not differentiated, with widely overlapping individual values and 95% confidence intervals, matching the finding that these subspecies were lumped together in STRUCTURE analyses using the whole data set. In contrast, *E*. *o*. *occidentalis* (blue) was clearly distinct. Admixed individuals (turtles having admixed genotypes or pure genotypes combined with mismatched mitochondrial haplotypes; orange) were also highly distinct from *E*. *o*. *orbicularis* and *E*. *o*. *galloitalica*, but not clearly differentiated from *E*. *o*. *occidentalis*.

### Cline analyses

The Atlantic and Mediterranean transects (Fig. 2a) were analyzed (Table S3) for the cyt *b* gene and microsatellites, whereas only microsatellites were examined for the transect running through the Ebro valley, because the widely syntopic occurrence of distinct mitochondrial lineages prevents the use of this marker system for cline analyses in this region.

**Figure 6:**
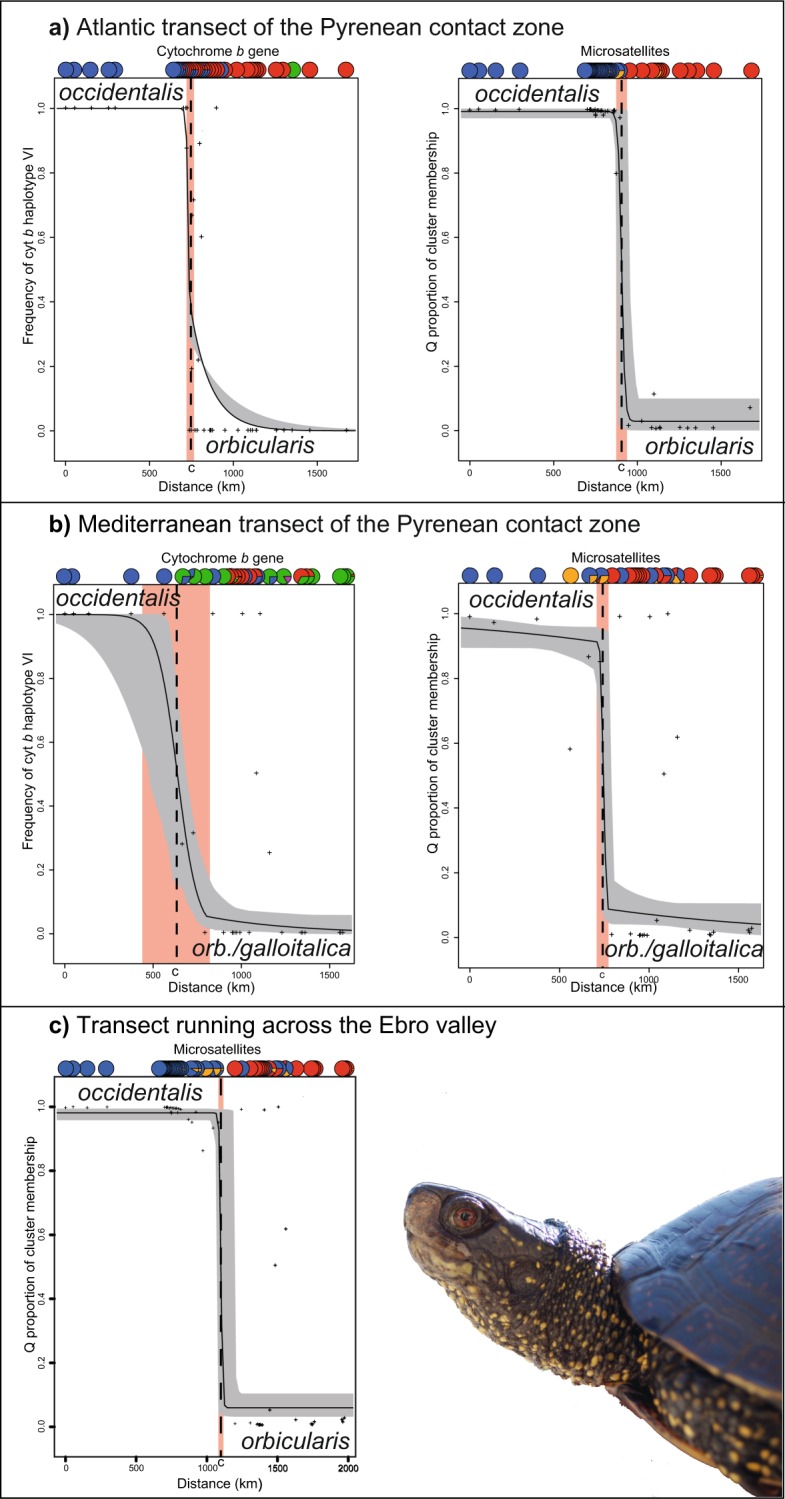
Maximum likelihood clines of **(a)** the Atlantic and **(b)** the Mediterranean transects of the Pyrenean contact zone and **(c)** the transect running across the Ebro valley over the associated fuzzy cline region (95% credible cline region, grey) as returned by the function hzar.plot.fzCline in HZAR. The broken line represents the cline center (**c**) and the orange frame the cline width (w). Above the curves are percentages for mitochondrial lineages or cluster assignment. For color-coding, see Figs 1 and 2. Inset: *Emys orbicularis orbicularis*; photo: Melita Vamberger. The figure was created using R^57^ and ADOBE ILLUSTRATOR CS6 (http://www.adobe.com/products/illustrator.html).

The cline analysis of the Atlantic transect revealed an extremely steep transition for mitochondrial and microsatellite data (Fig. 6a; Table S4). However, the cline centers of the two marker systems differed (Fig. 2a). For the cyt *b* gene, the center was estimated to be 735.73 km (95% confidence interval: 730.52–735.74 km) northeast of the reference site (Fig. 2a: site 8), with a cline width of 26.67 km (18.74–26.67 km). For microsatellites, the center was inferred to be more northerly, 906.14 km (875.44–949.60 km) distant to the reference site, with a cline width of 39.18 km (0.01–86.46 km). The cline center of the microsatellites approximately matches with the Pyrenees (Fig. 2a).

The analysis of the Mediterranean transect revealed for mtDNA data a slightly smoother cline with a larger introgression tail (Fig. 6b; Table S4). The cline center was inferred to be 637.24 km (520.29–675.60 km) distant to the reference site (Fig. 2a: site 7), with a cline width of 230.52 km (44.96–550.18 km). The center for the microsatellite cline was steeper and estimated to be 745.31 km (731.70–787.79 km) distant to the reference site, with a width of 40.03 km (1.75–98.60 km).

The third analysis, which examined the connection between the Atlantic and Mediterranean transects across the Ebro valley using microsatellites only, unveiled again a very steep cline (Fig. 6c; Table S4). Its center was estimated to be 1099.93 km (1082.71–1200.65 km) northeast of the reference site (Fig. 2a: site 8), with a cline width of only 21.2 km (0.01–109.7 km).

### Demographic analyses

The scenarios for our DIYABC calculations were well supported (Fig. S7). In the Atlantic range, the contact between *E*. *o*. *occidentalis* and *E*. *o*. *orbicularis* was dated to the Holocene, with an estimated average of 5,700 years BP (Fig. S8a), supporting that *E*. *o*. *orbicularis* is a Holocene invader of the Iberian Peninsula^24^. The contribution of *E*. *o*. *orbicularis* to the resulting population (the admixture rate *r*) was estimated to 0.26 (Fig. S8b). For the Mediterranean range, the contact among *E*. *o*. *occidentalis* and *E*. *o*. *galloitalica* was dated to the late Pleistocene, with an estimated average of 16,950 years BP. The contact of this admixed population with *E*. *o*. *orbicularis* was inferred to be very recent, with an estimated average of only 1,710 years BP. The admixture rates *r1* (contribution of the admixed population to the contact with *E*. *o*. *orbicularis*) and *r2* (contribution of *E*. *o*. *occidentalis* to the contact with *E*. *o*. *galloitalica*; Fig. 3) resulted in mean estimates of 0.80 and 0.38, respectively (Fig. S8b).

### Diversity within and divergence among pure and admixed populations

In the first analysis (Atlantic transect), we compared pure individuals of *E*. *o*. *occidentalis* (group 1) and *E*. *o*. *orbicularis* (group 2) with admixed populations from the Atlantic coast north (group 4) and south (group 5) of the Pyrenees (Fig. 2a; Table 1). Group 4 (admixed) showed the highest number of alleles (126), the highest average number of alleles per locus (8.4) and the lowest inbreeding coefficient (0.051). Group 5 (admixed) had the highest inbreeding coefficient (0.265), the highest number of segregating sites (15), and the highest nucleotide diversity (0.00725). The genetic diversity indices of the three other groups were similar and lower. *F*_ST_ values among populations ranged from 0.026 to 0.334 for microsatellites (Table S5), and the AMOVA indicated that 20.69% of the observed global genetic variance occurred between groups and 79.31% within groups. For mtDNA, *F*_ST_ values among the groups ranged from 0.000 to 0.975 (Table S5), and the AMOVA showed that 82.12% of the molecular variance were found among and 17.88% within the groups.

**Table 1:** Genetic diversity indices for two analyses comparing pure (1–3) and admixed groups (4–7).

Group	Microsatellites								mtDNA					
	*n*	*n*_*A*_	*n*_*Ā*_	*n*_*P*_	*AR*	*H*_*O*_	*H*_*E*_	*F*_*IS*_	*n*	*S*	*h*	*h*_*p*_	*Hd*	π (*10^−3^)
Group 1 (*occidentalis*)	35	102	6.8	26	6.022	0.483	0.616	0.219*	37	3	3	1	0.39	0.73
Group 2 (*orbicularis*)	35	120	8.0	14	7.017	0.604	0.736	0.182*	35	0	1	0	0	0
Group 4 (N Pyrenees)	16	126	8.4	15	7.923	0.737	0.775	0.051	16	0	1	0	0	0
Group 5 (SW Pyrenees)	35	111	7.4	12	6.421	0.509	0.690	0.265*	23	15	2	0	0.50	7.25
Group 1 (*occidentalis*)	35	102	6.800	16	6.023	0.483	0.616	0.219*	37	3	3	1	0.39	0.73
Group 2 (*orbicularis*)	35	120	8.000	19	7.017	0.604	0.736	0.182*	35	0	1	0	0	0
Group 3 (*galloitalica*)	38	133	8.867	15	7.427	0.630	0.700	0.105*	38	0	1	0	0	0
Group 6 (Spanish Mediterranean coast)	35	133	8.867	15	7.826	0.553	0.755	0.271*	28	18	3	2	0.67	8.16
Group 7 (French Mediterranean coast)	25	134	8.933	19	8.501	0.761	0.795	0.044*	25	22	3	1	0.55	7.46

The results of the two analyses are separated by a bold line.

*n* = sample size; *n*_*A*_ = total number of alleles per group; *n*_Ā_ = mean number of alleles per locus; *n*_*P*_ = number of private alleles; *AR* = allelic richness; *H*_*O*_ = average expected heterozygosity; *H*_*E*_ = average expected heterozygosity; *F*_*IS*_ = average inbreeding coefficient; *statistically significant; *S* = number of segregating sites; *h* = number of haplotypes; *h*_P_ = number of private haplotypes; *Hd* = Haplotype diversity, π = nucleotide diversity.

For the second analysis (Mediterranean transect), we compared pure individuals of *E*. *o*. *orbicularis* (group 2), *E*. *o*. *galloitalica* (group 3) and *E*. *o*. *occidentalis* (group 1) with admixed populations from the Spanish and French Mediterranean coast (group 6 and 7, respectively; Fig. 2a; Table 1). Both admixed groups, 6 and 7, showed high indices. The genetic diversity indices of the three pure groups were lower. *F*_ST_ values among groups ranged from 0.057 to 0.36 for microsatellites (Table S6), and the AMOVA indicated that 18.77% of the observed global genetic variance occurred between groups and 81.83% within groups. For the mtDNA the *F*_ST_ values varied between 0.003 and 1.00, and the AMOVA indicated that of the 71.39% molecular variance were found between and 28.61% within the groups.

## Discussion

Our data set of more than 800 samples covers the entire distribution range of the European pond turtle in Morocco, the Iberian Peninsula and large parts of its range in France, with a focus on the Pyrenean contact zone of three subspecies (Fig. 1; *Emys orbicularis orbicularis*, *E*. *o*. *galloitalica* and *E*. *o*. *occidentalis*). This region was already examined in a previous study^24^, which found only few turtles with mixed ancestry, but extensive cytonuclear discordance, suggestive of incipient isolation mechanisms reducing gene flow. However, due to patchy sampling and less informative genetic markers compared to our study, only preliminary conclusions could be drawn.

Here we present results using dense sampling from the contact zone and beyond, based on two genetic marker systems (15 microsatellite loci, cyt *b* gene), which have been used successfully to scrutinize fine-scale gene flow in another contact zone of the *E*. *orbicularis* complex in southern Italy^13^. Most notably, using approximately six-fold greater sampling and fine-scale analyses (Fig. 4 and Fig. S6), we found surprisingly little admixture among the subspecies. In particular, gene flow between *E*. *o*. *occidentalis* and the other two subspecies was negligible and with steep hybrid clines (Fig. 6). Gene flow occurred mainly from *occidentalis* into *galloitalica* and *orbicularis*, resulting in mitochondrial introgression in the opposite direction as reflected by discordant cline centers for mtDNA and microsatellites. Fine-scale analyses of the hybridization pattern of *E*. *o*. *galloitalica* and *E*. *o*. *orbicularis* also revealed little admixture (Fig. S6, top), even though the population from the Camargue and many other French populations had been previously identified as hybrid swarms of these two taxa^57^.

Our analyses found *E*. *o*. *occidentalis*, *E*. *o*. *orbicularis* and *E*. *o*. *galloitalica* distinct in mitochondrial and nuclear markers, with the latter two subspecies being only poorly differentiated. Despite weak differentiation of *E*. *o*. *orbicularis* and *E*. *o*. *galloitalica* (Table S6; *F*_ST_ value 0.083 for microsatellites), gene flow was largely unidirectional from *orbicularis* into *galloitalica* (Fig. S6). Compared to these two subspecies, *E*. *o*. *occidentalis* was much more distinct (Table S6; pairwise *F*_ST_ values 0.334 and 0.360 for microsatellites). Gene flow among all three taxa was evident (Figs 4 and S6). However, we recorded not a single turtle with a genotype corresponding to pure or mostly pure *E*. *o*. *galloitalica* or *E*. *o*. *orbicularis* (red columns) combined with a mitochondrial haplotype of *E*. *o*. *occidentalis* (lineage VI, blue). In contrast, there were many pure genotypes of *E*. *o*. *occidentalis* (blue columns) or admixed genotypes combined with haplotypes of *E*. *o*. *orbicularis* (lineage II, red) or *E*. *o*. *galloitalica* (lineage V, green). This provides evidence for unidirectional gene flow from *E*. *o*. *occidentalis* into *E*. *o*. *orbicularis* and *E*. *o*. *galloitalica*. This pattern was also supported by PCAs using microsatellite data in that admixed individuals clustered with *E*. *o*. *occidentalis* but are distinct from the two other taxa (Fig. 5).

This pattern of unidirectional gene flow could be caused by several factors, among them genetic incompatibility^60^. Other, well-known reasons for cytonuclear discordance are sex-biased dispersal or asymmetric mating preferences^60,61^. Male pond turtles are known to prefer large females, promising higher reproductive success^24,62^. In this vein, the larger average body size of *E*. *o*. *orbicularis* compared to *E*. *o*. *occidentalis*^22,63^ may contribute to the observed genetic pattern.

We found only turtles harboring the mitochondrial lineage of *E*. *o*. *orbicularis* (II) or *E*. *o*. *galloitalica* (V) north of the Pyrenees. The vast majority of turtles there represented genotypically pure *E*. *o*. *orbicularis* or *E*. *o*. *galloitalica* and their hybrids (Figs 4 and S6), corresponding to the red cluster in Fig. 2. Yet, among 133 turtles we found one individual from the Marais d’Orx, Landes, that could be unambiguously identified as a backcross with *E*. *o*. *occidentalis*, supporting very weak genetic impact from this taxon in southwesternmost France, as already suggested by a previous record of a turtle with haplotype VIa at Pau, Aquitaine^24,43^.

South of the Pyrenees admixture with *E*. *o*. *occidentalis* was more pronounced. In the western Ebro Basin, we recorded turtles harboring haplotypes of lineages II and VI (Fig. 2). Nevertheless, most individuals represented genotypically pure *E*. *o*. *occidentalis* (blue cluster; Fig. 4: sites 16–43), irrespective of their mitochondrial haplotype. Only 16 out of 388 turtles were genotypically admixed. Eight individuals were backcrosses, while the remaining turtles represented F_1_ and F_2_ hybrids (Fig. 4). In the Mediterranean transect, where a putative glacial refuge of *E*. *o*. *galloitalica* was located along the northern Mediterranean coast of Spain^24^, haplotypes of mitochondrial lineage V, characteristic for *E*. *o*. *galloitalica*, occurred together with haplotypes of lineages II and VI. However, the isolated haplotypes of lineage VI in the northernmost sites (Fig. 2a, sites 46, 48, 51 and 56) represented most probably introduced turtles^64,65^ and any conclusions for this area can therefore be misleading. The general pattern here was the same as in the western Ebro Basin, i.e. largely unidirectional gene flow from *E*. *o*. *occidentalis* into the other two taxa, even though more backcrosses occurred. This situation mirrors a complex biogeographic scenario, with a northward Holocene range expansion of *E*. *o*. *occidentalis* across the Iberian Peninsula^26,28^, largely stationary populations of *E*. *o*. *galloitalica* along the Spanish Mediterranean coast, and a very recent Holocene invasion of the peninsula by *E*. *o*. *orbicularis*^24^ (Fig. S9). This scenario is supported by our Approximate Bayesian Computing, dating the contact of the northwards expanding *E*. *o*. *occidentalis* with *E*. *o*. *galloitalica* to the late Pleistocene (approx. 17,000 years BP), while the genetic impact of *E*. *o*. *orbicularis* commenced only approximately 5,700 years BP. During its northward range expansion, the topology forced *E*. *o*. *occidentalis* to follow two major routes, one along the Mediterranean coast to enter the Ebro valley from the east, and the other along the Atlantic coastal corridor to enter all the river basins dissecting the Spanish Meseta (Guadiana, Guadalquivir, Tajo, Duero, Miño; Fig. S9)^28^. A similar pattern of an expansion from the south of the Guadalquivir River to the Pyrenees was also described for painted frogs (*Discoglossus* spp.^66^).

For the pond turtle, the antidromic range expansions of *E*. *o*. *occidentalis* and *E*. *o*. *orbicularis* lead to admixture of these subspecies in the Ebro valley and with *E*. *o*. *galloitalica* in its former glacial refuge at the Mediterranean coast of Spain (Fig. S9). In contrast to the other two taxa, *E*. *o*. *galloitalica* was largely imprisoned in its refuge consisting of isolated short rivers, without many possibilities to disperse. This situation explains why until today largely pure *E*. *o*. *galloitalica* occur in some sites there.

The observed hybridization patterns across our study region suggest that the three taxa correspond to different stages in the speciation process, with *E*. *o*. *occidentalis* representing the most advanced stage. A future taxonomic investigation that includes also the subspecies from North Africa, Eastern Europe and Asia, not covered by the present study, has to resolve whether an elevation of one or more subspecies to full species level matches their evolutionary divergence better than lumping all together in one species, a situation reminiscent of the recent taxonomic break-up of smooth^67^ and crested newts^68^, common toads^69^, tree frogs^70,71^, slow worms^72^ and grass snakes^73,74^.

## Electronic supplementary material

Supporting Information

## Data Availability

The data sets generated and analyzed during the current study are available from the corresponding author on reasonable request.
