# Supplementary material for: Complex hybridization patterns in European pond turtles (Emys orbicularis) in the Pyrenean Region
Source: Sci Rep. 2018 Oct 29;8:15925. doi: 10.1038/s41598-018-34178-0 (PMC6206128; doi:10.1038/s41598-018-34178-0)
Supplement: Supplementary file 1 — Supporting Information [file 41598_2018_34178_MOESM1_ESM.pdf]

Complex hybridization patterns in European pond turtles (*Emys orbicularis*) in the Pyrenean Region

Julia Pöschel, Botond Heltai, Eva Graciá, Marc Franch Quintana, Guillermo Velo-Antón, Oscar Arribas, Aitor Valdeón, Michael Wink, Uwe Fritz, Melita Vamberger

Scientific Reports

DOI:10.1038/s41598-018-34178-0

Supporting Information

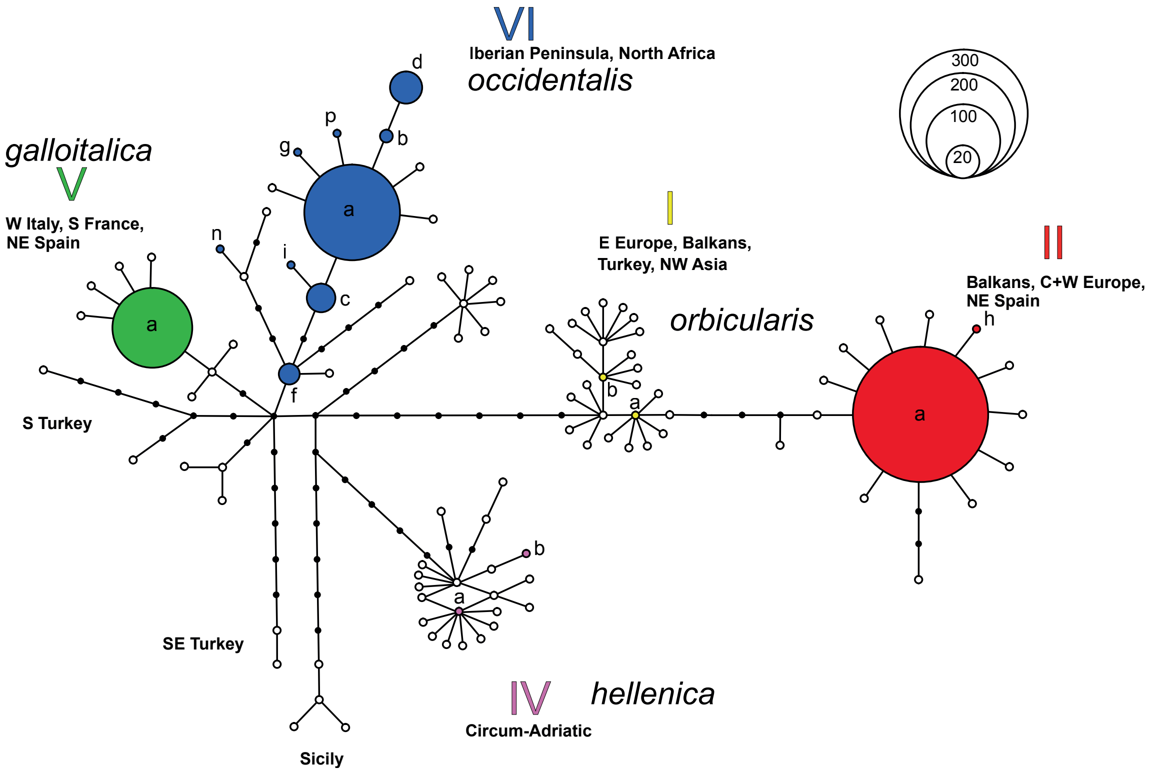

Figure S1. Parsimony network of all known cyt *b* haplotypes of *Emys orbicularis* and *E. trinacris* (Lenk *et al.*, 1999; Fritz *et al.*, 2005, 2007, 2009; Pedall *et al.*, 2011; Prusak *et al.*, 2011; Velo-Antón *et al.*, 2011, 2015; Stuckas *et al.*, 2014; Vamberger *et al.*, 2015; this study). For subspecies found in the study region, Roman numerals indicate haplotype lineages. Circle size corresponds for the study region to haplotype frequency; missing haplotypes, small black dots. Each line connecting two haplotypes represents one mutation step. In the Iberian Peninsula and Western Europe, only the subspecies *E. o. occidentalis*, *E. o. galloitalica* and *E. o. orbicularis* are native (Fritz, 2003). The figure was created using ADOBE ILLUSTRATOR CS6 (http://www.adobe.com/products/illustrator.html).

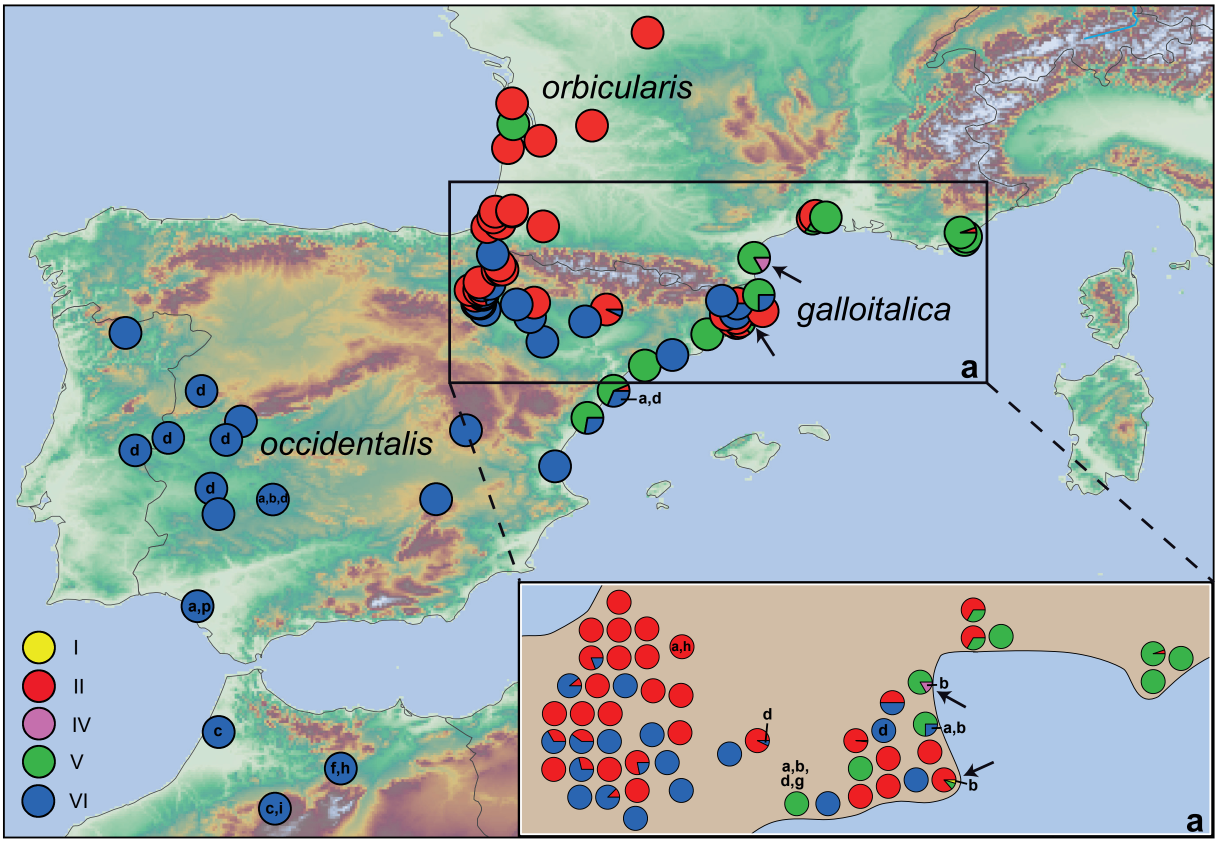

Figure S2. Geographical distribution of cyt *b* haplotypes of the five mitochondrial lineages (I, II, IV, V, VI) recorded in the study region. Individual haplotypes of each lineage bear letters; circles without letters indicate for each lineage haplotype a. The densely sampled Pyrenees region **(a)** is shown in detail with arbitrarily spread sampling sites. Arrows highlight non-native turtles with mtDNA lineages I and IV. V. Map was created using ARCGIS 10.2 (http://www.esri.com/arcgis) and ADOBE ILLUSTRATOR CS6 (http://www.adobe.com/products/illustrator.html).

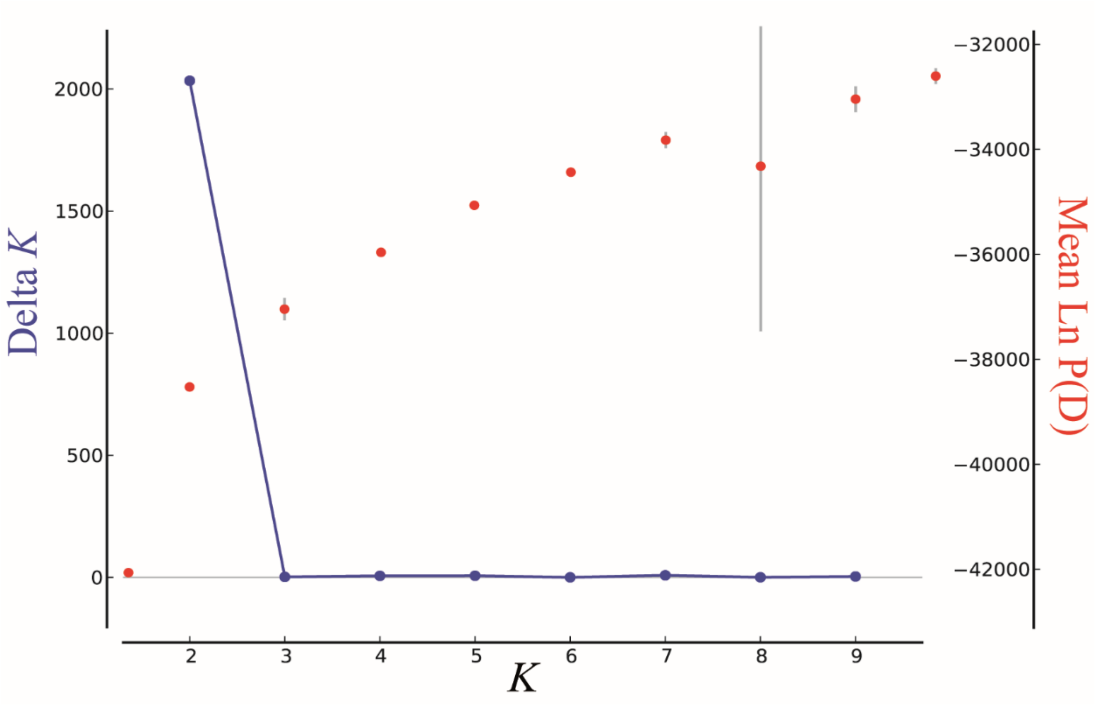

Figure S3. Delta *K* and Ln P(D) values for STRUCTURE runs using the whole data set (732 samples). The modal value is at *K* = 2.

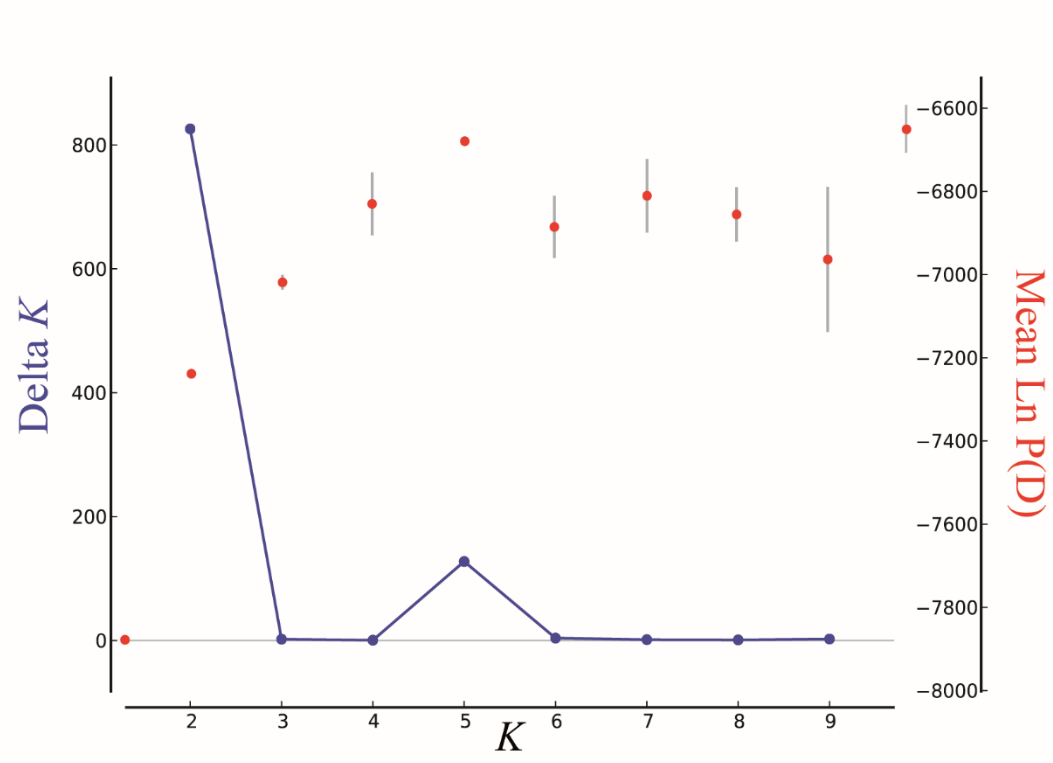

Figure S4. Delta *K* and Ln P(D) values for STRUCTURE runs for subsample 1 (north of the Pyrenees, 133 samples). The modal value is at *K* = 2.

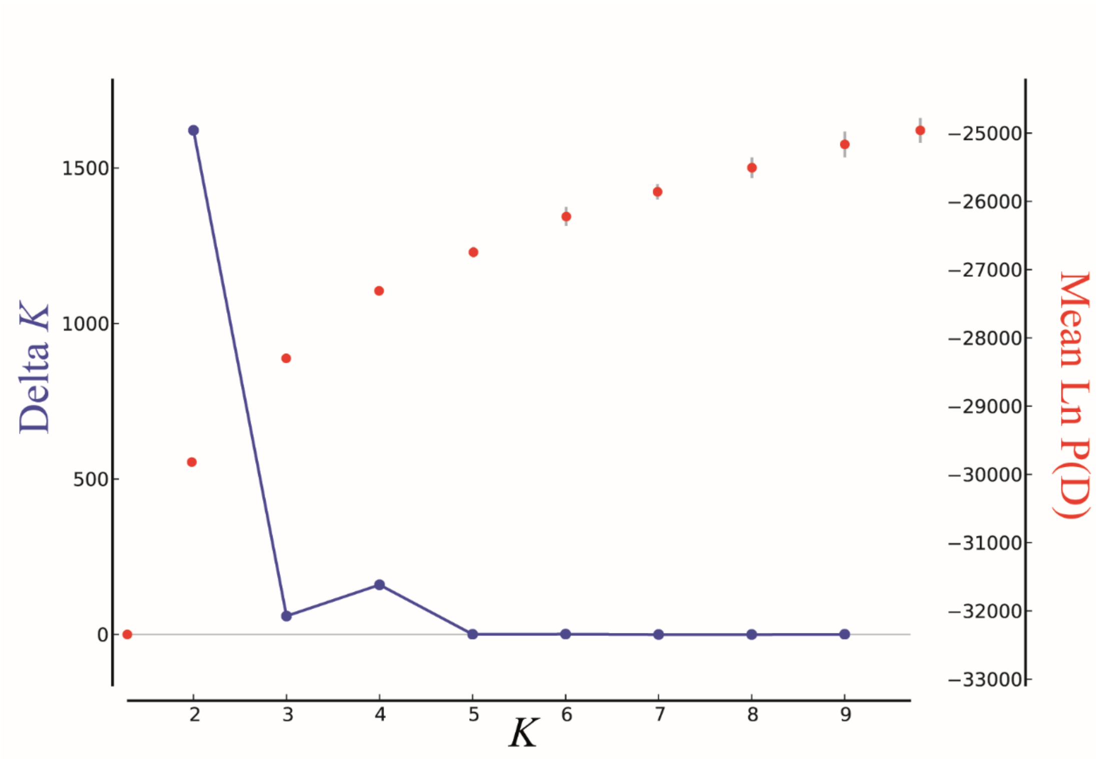

Figure S5. Delta *K* and Ln P(D) values for STRUCTURE runs for subsample 2 (south of the Pyrenees, 599 samples). The modal value is at *K* = 2.

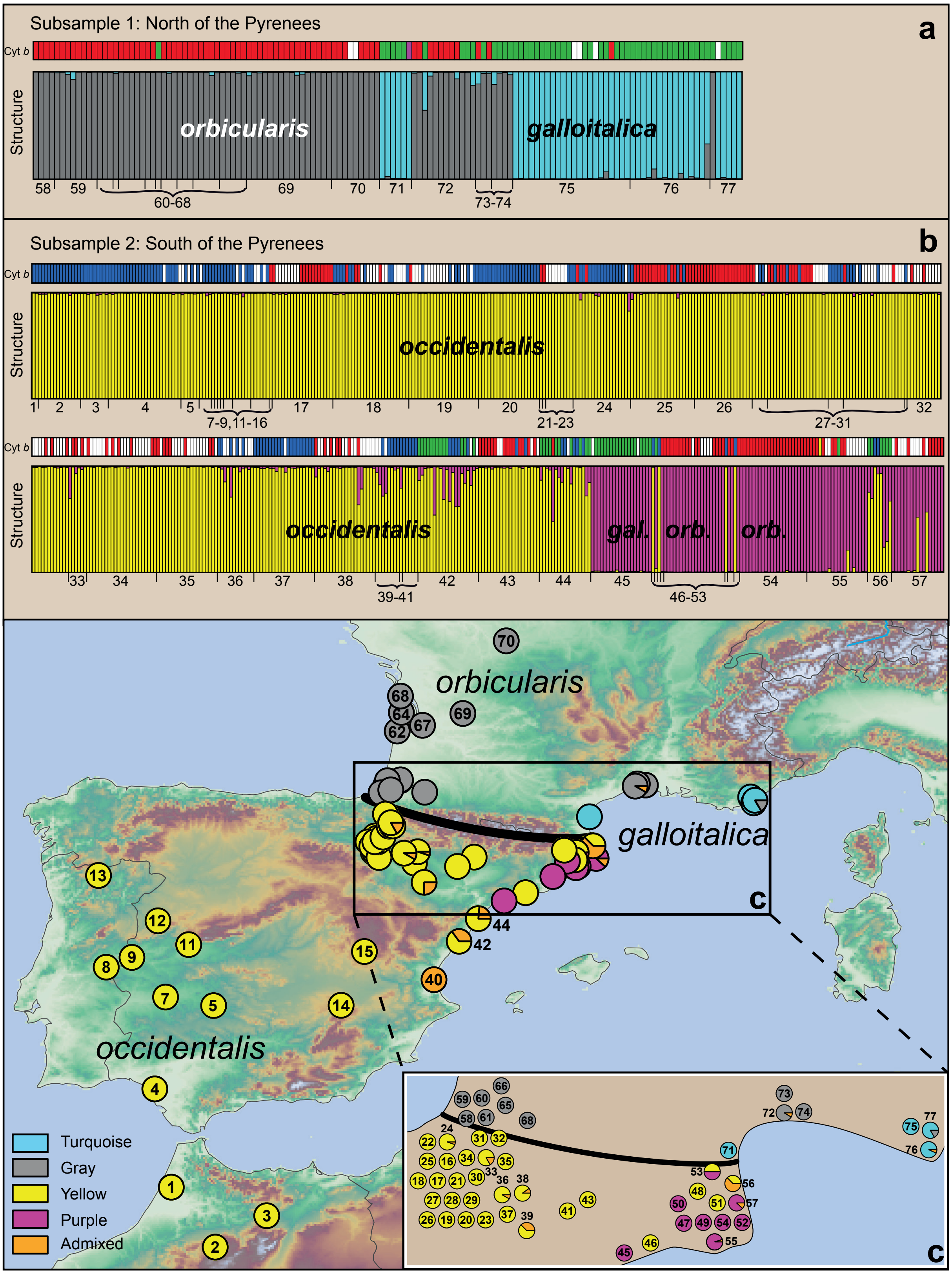

Fig. S6. Genotypic structuring for the two subsamples **(a)** north and **(b)** south of the Pyrenees. The black line in the map represents the Pyrenees as divide between the two subsamples. **(c)** The densely sampled Pyrenees region is shown in detail with arbitrarily spread sampling sites. For further explanation, see Figure 4. Map was created using ARCGIS 10.2 (http://www.esri.com/arcgis) and ADOBE ILLUSTRATOR CS6 (http://www.adobe.com/products/illustrator.html).

Atlantic range: *E. o. occidentalis* x *E. o. orbicularis*

Microsatellites

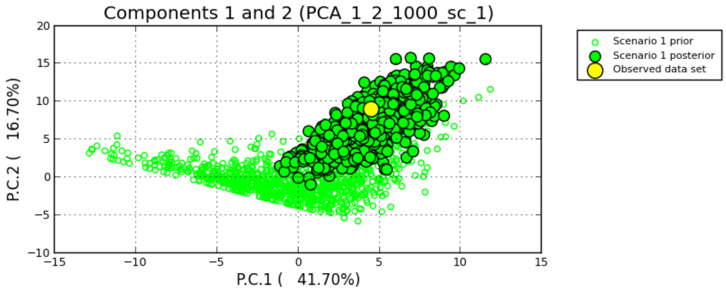

mtDNA

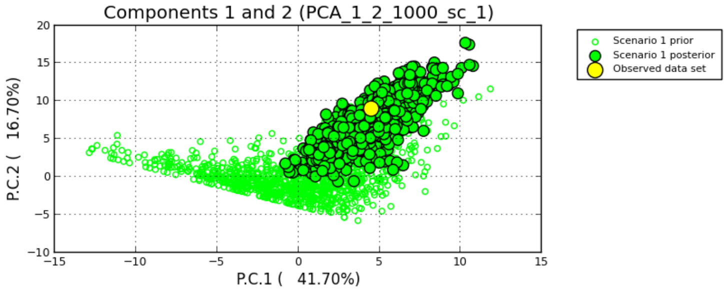

Mediterranean range: *E. o. occidentalis x E. o. galloitalica* x *E. o. orbicularis*

Microsatellites

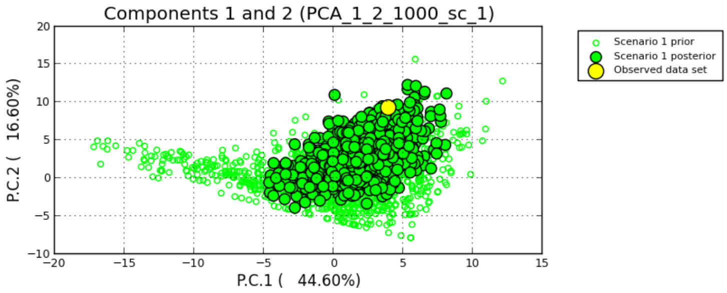

mtDNA

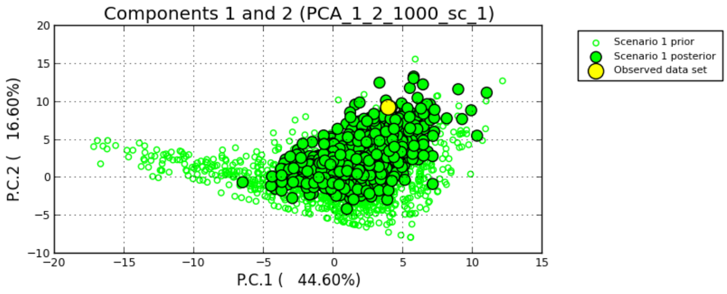

Figure S7. Principal Component Analysis (PCA) plots to perform model checking in DIYABC for the contact between *E. o. occidentalis* and *E. o. orbicularis* (Atlantic range) and for the contact between *E. o. occidentalis*, *E. o. galloitalica* and *E. o. orbicularis* (Mediterranean range), respectively. The large yellow dots represent where the observed data fits within a representative set of 1,000 simulated datasets (small dots) and the posterior predictive distributions (larger green dots).

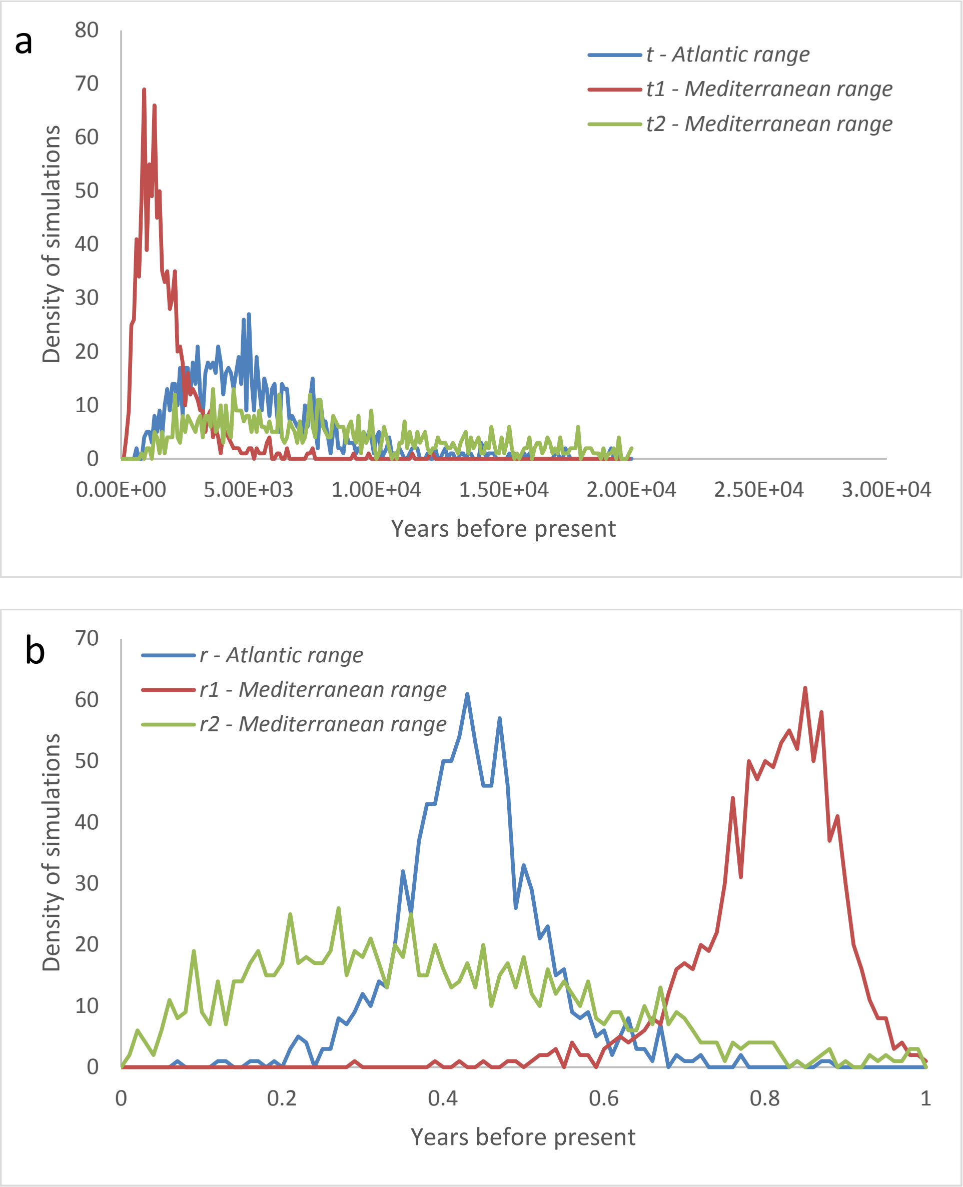

Figure S8. Demographic inferences with DIYABC of the secondary contact between pond turtle lineages. **(a)** Time before present (BP) when the secondary contact established; **(b)** rate of admixture between the lineages.

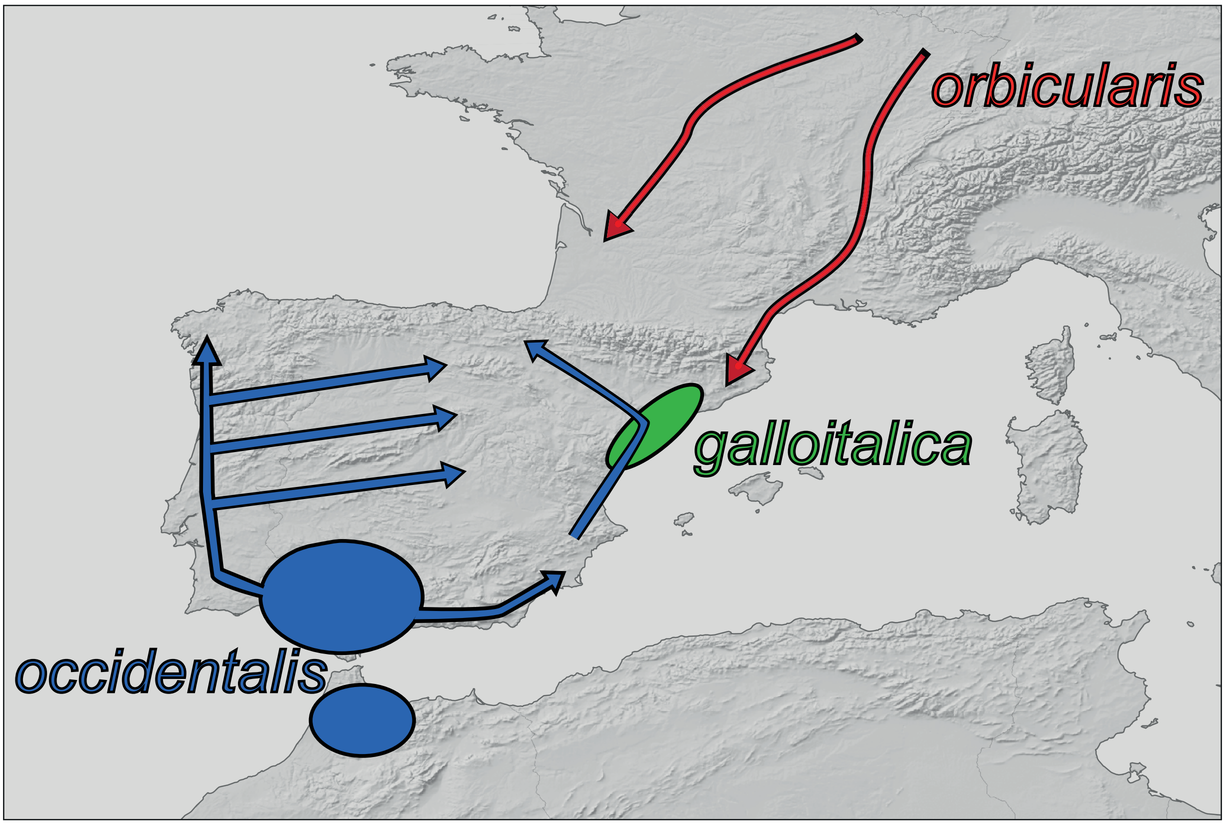

Figure S9. Putative glacial refuges and Holocene range shifts of *Emys orbicularis orbicularis*, *E. o. galloitalica* and *E. o. occidentalis* (schematic). The glacial refuge of *E. o. orbicularis* was in the Balkan Peninsula from whence it spread northwards and westwards and reached western France and northern Spain (Lenk *et al*., 1999; Sommer *et al*., 2009). Map was created using ARCGIS 10.2 (http://www.esri.com/arcgis) and ADOBE ILLUSTRATOR CS6 (http://www.adobe.com/products/illustrator.html).

Table S1. Studied samples of *Emys orbicularis* , their geographical provenance and genetic identity.

#	MTD-T	Country	Locality	Sampling Site	Haplotype Cyt *b*	*K* =2		*K* =2 (subset north)		*K* =2 (subset south)		N	E
						Red	Blue	Grey	Turqouis	Yellow	Purple		
1	6420	Morocco	Kenitra	1	VIc	0.00	1.00	n/a	n/a	1.00	0.00	34.85	-6.07
2	6421	Morocco	Kenitra	1	VIc	0.00	1.00	n/a	n/a	1.00	0.00	34.85	-6.07
3	5103	Morocco	Middle Atlas	2	VIc	0.01	0.99	n/a	n/a	0.99	0.01	33.55	-5.11
4	5104	Morocco	Middle Atlas	2	VIc	0.01	0.99	n/a	n/a	0.99	0.01	33.55	-5.11
5	5105	Morocco	Middle Atlas	2	VIc	0.01	0.99	n/a	n/a	0.99	0.01	33.55	-5.11
6	6422	Morocco	Middle Atlas	2	VIc	0.01	1.00	n/a	n/a	1.00	0.00	33.55	-5.11
7	5367	Morocco	Middle Atlas	2	VIc	0.00	1.00	n/a	n/a	1.00	0.00	33.55	-5.11
8	9084	Morocco	Middle Atlas	2	VIi	0.01	0.99	n/a	n/a	0.99	0.01	33.55	-5.11
9	9085	Morocco	Middle Atlas	2	VIc	0.04	0.96	n/a	n/a	0.99	0.01	33.55	-5.11
10	9086	Morocco	Middle Atlas	2	VIc	0.00	1.00	n/a	n/a	1.00	0.00	33.55	-5.11
11	9087	Morocco	Middle Atlas	2	VIc	0.01	0.99	n/a	n/a	0.99	0.01	33.55	-5.11
12	9088	Morocco	Middle Atlas	2	VIc	0.00	1.00	n/a	n/a	1.00	0.00	33.55	-5.11
13	5873	Morocco	Middle Atlas	2	VIc	0.04	0.96	n/a	n/a	0.97	0.03	33.55	-5.11
14	5874	Morocco	Middle Atlas	2	VIc	0.01	1.00	n/a	n/a	1.00	0.00	33.55	-5.11
15	5875	Morocco	Middle Atlas	2	VIc	0.00	1.00	n/a	n/a	1.00	0.00	33.55	-5.11
16	5876	Morocco	Middle Atlas	2	VIc	0.09	0.91	n/a	n/a	0.99	0.01	33.55	-5.11
17	7306	Morocco	Rif	3	VIf	0.01	0.99	n/a	n/a	0.99	0.01	34.23	-3.99
18	9079	Morocco	Rif	3	VIf	0.00	1.00	n/a	n/a	1.00	0.00	34.23	-3.99
19	9080	Morocco	Rif	3	VIf	0.02	0.98	n/a	n/a	0.99	0.01	34.23	-3.99
20	9081	Morocco	Rif	3	VIf	0.01	0.99	n/a	n/a	1.00	0.00	34.23	-3.99
21	9082	Morocco	Rif	3	VIf	0.00	1.00	n/a	n/a	1.00	0.00	34.23	-3.99
22	9083	Morocco	Rif	3	VIf	0.04	0.96	n/a	n/a	0.98	0.02	34.23	-3.99
23	2456	Morocco	Rif	3	VIf	0.01	0.99	n/a	n/a	0.99	0.01	34.23	-3.99
24	2749	Morocco	Rif	3	VIf	0.01	0.99	n/a	n/a	1.00	0.00	34.23	-3.99
25	3816	Morocco	Rif	3	VIn	0.02	0.98	n/a	n/a	0.99	0.01	34.23	-3.99
26	9756	Spain	Andalucia: Huelva: Doñana National Park	4	VIa	0.01	0.99	n/a	n/a	0.99	0.01	36.99	-6.42
27	9757	Spain	Andalucia: Huelva: Doñana National Park	4	VIa	0.02	0.98	n/a	n/a	0.99	0.01	36.99	-6.42
28	9758	Spain	Andalucia: Huelva: Doñana National Park	4	VIa	0.00	1.00	n/a	n/a	1.00	0.00	36.99	-6.42
29	9759	Spain	Andalucia: Huelva: Doñana National Park	4	VIp	0.00	1.00	n/a	n/a	1.00	0.00	36.99	-6.42
30	9760	Spain	Andalucia: Huelva: Doñana National Park	4	VIa	0.00	1.00	n/a	n/a	1.00	0.00	36.99	-6.42
31	9761	Spain	Andalucia: Huelva: Doñana National Park	4	VIa	0.00	1.00	n/a	n/a	1.00	0.00	36.99	-6.42
32	9762	Spain	Andalucia: Huelva: Doñana National Park	4	VIa	0.01	0.99	n/a	n/a	0.99	0.01	36.99	-6.42
33	9763	Spain	Andalucia: Huelva: Doñana National Park	4	VIa	0.00	1.00	n/a	n/a	1.00	0.00	36.99	-6.42
34	9764	Spain	Andalucia: Huelva: Doñana National Park	4	VIa	0.00	1.00	n/a	n/a	1.00	0.00	36.99	-6.42
35	9765	Spain	Andalucia: Huelva: Doñana National Park	4	VIa	0.00	1.00	n/a	n/a	1.00	0.00	36.99	-6.42
36	9766	Spain	Andalucia: Huelva: Doñana National Park	4	VIa	0.01	0.99	n/a	n/a	1.00	0.01	36.99	-6.42
37	9767	Spain	Andalucia: Huelva: Doñana National Park	4	VIa	0.00	1.00	n/a	n/a	1.00	0.00	36.99	-6.42
38	9768	Spain	Andalucia: Huelva: Doñana National Park	4	VIa	0.00	1.00	n/a	n/a	1.00	0.00	36.99	-6.42
39	9769	Spain	Andalucia: Huelva: Doñana National Park	4	VIa	0.00	1.00	n/a	n/a	1.00	0.00	36.99	-6.42

#	MTD-T	Country	Locality	Sampling Site	Haplotype Cyt *b*	*K* =2		*K* =2 (subset north)		*K* =2 (subset south)		N	E
■ Table 1 continued						Red	Blue	Grey	Turqouis	Yellow	Purple		
40	9770	Spain	Andalucia: Huelva: Doñana National Park	4	VIa	0.00	1.00	n/a	n/a	1.00	0.00	36.99	-6.42
41	9771	Spain	Andalucia: Huelva: Doñana National Park	4	VIa	0.01	1.00	n/a	n/a	1.00	0.01	36.99	-6.42
42	9772	Spain	Andalucia: Huelva: Doñana National Park	4	VIa	0.01	0.99	n/a	n/a	0.99	0.01	36.99	-6.42
43	9773	Spain	Andalucia: Huelva: Doñana National Park	4	VIa	0.01	0.99	n/a	n/a	0.99	0.01	36.99	-6.42
44	9774	Spain	Andalucia: Huelva: Doñana National Park	4	n/a	0.00	1.00	n/a	n/a	1.00	0.00	36.99	-6.42
45	9775	Spain	Andalucia: Huelva: Doñana National Park	4	VIa	0.01	0.99	n/a	n/a	0.99	0.01	36.99	-6.42
46	9776	Spain	Andalucia: Huelva: Doñana National Park	4	VIa	0.01	1.00	n/a	n/a	1.00	0.01	36.99	-6.42
47	9777	Spain	Andalucia: Huelva: Doñana National Park	4	VIa	0.01	0.99	n/a	n/a	0.99	0.01	36.99	-6.42
48	9778	Spain	Andalucia: Huelva: Doñana National Park	4	VIa	0.00	1.00	n/a	n/a	1.00	0.00	36.99	-6.42
49	9779	Spain	Andalucia: Huelva: Doñana National Park	4	n/a	0.01	0.99	n/a	n/a	1.00	0.00	36.99	-6.42
50	15345	Spain	Extremadura: Zarza-Capilla	5	n/a	0.01	0.99	n/a	n/a	0.98	0.02	38.80	-5.14
51	15346	Spain	Extremadura: Zarza-Capilla	5	VIa	0.00	1.00	n/a	n/a	1.00	0.00	38.80	-5.14
52	15347	Spain	Extremadura: Zarza-Capilla	5	n/a	0.01	0.99	n/a	n/a	1.00	0.00	38.80	-5.14
53	15348	Spain	Extremadura: Zarza-Capilla	5	VIa	0.00	1.00	n/a	n/a	1.00	0.00	38.80	-5.14
54	15349	Spain	Extremadura: Zarza-Capilla	5	VIb	n/a	n/a	n/a	n/a	n/a	n/a	38.80	-5.14
55	15350	Spain	Extremadura: Zarza-Capilla	5	n/a	0.14	0.86	n/a	n/a	0.99	0.01	38.80	-5.14
56	15351	Spain	Extremadura: Zarza-Capilla	5	VId	0.01	1.00	n/a	n/a	1.00	0.00	38.80	-5.14
57	15358	Spain	Extremadura: Hornachos	6	VIa	n/a	n/a	n/a	n/a	n/a	n/a	38.55	-6.07
58	15354	Spain	Extremadura: Embalse de Cornalvo	7	n/a	0.01	0.99	n/a	n/a	0.99	0.01	38.99	-6.19
59	15355	Spain	Extremadura: Embalse de Cornalvo	7	VId	0.00	1.00	n/a	n/a	1.00	0.00	38.99	-6.19
60	15356	Spain	Extremadura: Embalse de Cornalvo	7	VId	0.02	0.98	n/a	n/a	0.97	0.03	38.99	-6.19
61	15357	Spain	Extremadura: Embalse de Cornalvo	7	VId	0.02	0.98	n/a	n/a	0.99	0.01	38.99	-6.19
62	7317	Spain	Cáceres: Tajo River Basin: Cedillo	8	VId	0.01	0.99	n/a	n/a	0.99	0.01	39.64	-7.48
63	7316	Spain	Cáceres: Erjas River Basin: Alcántara	9	VId	0.00	1.00	n/a	n/a	1.00	0.00	39.85	-6.92
64	15359	Spain	Extremadura: Parque Nacional de Monfragüe	10	VId	n/a	n/a	n/a	n/a	n/a	n/a	39.81	-5.94
65	15352	Spain	Extremadura: Embalse de Cornalvo	11	VIa	n/a	n/a	n/a	n/a	n/a	n/a	40.13	-5.69
66	15353	Spain	Extremadura: Embalse de Cornalvo	11	VIa	0.00	1.00	n/a	n/a	1.00	0.00	40.13	-5.69
67	3016	Spain	Castilla y León: Salamanca: Tenebron: Laguna Grande	12	VId	0.01	0.99	n/a	n/a	0.99	0.01	40.64	-6.36
68	3253	Portugal	Vila Real: Tâmega River	13	VIa	0.00	1.00	n/a	n/a	1.00	0.00	41.63	-7.65
69	3254	Portugal	Vila Real: Tâmega River	13	VIa	0.01	1.00	n/a	n/a	1.00	0.01	41.63	-7.65
70	3256	Portugal	Vila Real: Tâmega River	13	VIa	0.01	0.99	n/a	n/a	1.00	0.00	41.63	-7.65
71	15337	Spain	Castilla-La Mancha: Albacete: Laguna Ojos de Villaverde	14	VIa	0.01	0.99	n/a	n/a	0.99	0.01	38.81	-2.37
72	15338	Spain	Castilla-La Mancha: Albacete: Laguna Ojos de Villaverde	14	n/a	0.01	0.99	n/a	n/a	0.99	0.01	38.81	-2.37
73	15339	Spain	Castilla-La Mancha: Albacete: Laguna Ojos de Villaverde	14	VIa	0.01	1.00	n/a	n/a	1.00	0.00	38.81	-2.37
74	15340	Spain	Castilla-La Mancha: Albacete: Laguna Ojos de Villaverde	14	n/a	0.09	0.92	n/a	n/a	0.96	0.04	38.81	-2.37
75	15342	Spain	Castilla-La Mancha: Albacete: Laguna Ojos de Villaverde	14	VIa	0.00	1.00	n/a	n/a	1.00	0.00	38.81	-2.37
76	15343	Spain	Castilla-La Mancha: Albacete: Laguna Ojos de Villaverde	14	VIa	0.01	0.99	n/a	n/a	0.99	0.01	38.81	-2.37
77	15331	Spain	Castilla-La Mancha: Cuenca: Lagunas de Cañada del Hoyo	15	VIa	0.00	1.00	n/a	n/a	1.00	0.00	39.98	-1.86
78	15332	Spain	Castilla-La Mancha: Cuenca: Lagunas de Cañada del Hoyo	15	n/a	0.01	0.99	n/a	n/a	1.00	0.00	39.98	-1.86
79	15333	Spain	Castilla-La Mancha: Cuenca: Lagunas de Cañada del Hoyo	15	n/a	0.01	1.00	n/a	n/a	1.00	0.00	39.98	-1.86
80	15334	Spain	Castilla-La Mancha: Cuenca: Lagunas de Cañada del Hoyo	15	VIa	0.00	1.00	n/a	n/a	1.00	0.00	39.98	-1.86

#	MTD-T	Country	Locality	Sampling Site	Haplotype Cyt *b*	*K* =2		*K* =2 (subset north)		*K* =2 (subset south)		N	E
**■ Table 1 continued**						**Red**	**Blue**	**Grey**	**Turqouis**	**Yellow**	**Purple**		
81	15335	Spain	Castilla-La Mancha: Cuenca: Lagunas de Cañada del Hoyo	15	n/a	0.00	1.00	n/a	n/a	1.00	0.00	39.98	-1.86
82	15336	Spain	Castilla-La Mancha: Cuenca: Lagunas de Cañada del Hoyo	15	VIa	0.00	1.00	n/a	n/a	1.00	0.00	39.98	-1.86
83	7323	Spain	Navarra: Ribera del Arga Aragón: Peralta: Soto de La Muga	16	IIa	0.01	0.99	n/a	n/a	0.99	0.01	42.36	-1.79
84	4333	Spain	Navarra: Ribera Arga-Aragón: Villafranca: Badina Escudera	17	IIa	0.02	0.98	n/a	n/a	0.99	0.01	42.27	-1.70
85	7324	Spain	Navarra: Ribera Arga-Aragón: Villafranca: Badina Escudera	17	IIa	n/a	n/a	n/a	n/a	n/a	n/a	42.27	-1.70
86	3001	Spain	Navarra: Ribera Arga-Aragón: Villafranca: Badina Escudera	17	n/a	0.00	1.00	n/a	n/a	1.00	0.00	42.27	-1.70
87	3002	Spain	Navarra: Ribera Arga-Aragón: Villafranca: Badina Escudera	17	n/a	0.02	0.98	n/a	n/a	0.99	0.01	42.27	-1.70
88	3003	Spain	Navarra: Ribera Arga-Aragón: Villafranca: Badina Escudera	17	n/a	n/a	n/a	n/a	n/a	n/a	n/a	42.27	-1.70
89	3004	Spain	Navarra: Ribera Arga-Aragón: Villafranca: Badina Escudera	17	n/a	0.01	1.00	n/a	n/a	1.00	0.01	42.27	-1.70
90	3006	Spain	Navarra: Ribera Arga-Aragón: Villafranca: Badina Escudera	17	n/a	0.01	0.99	n/a	n/a	0.99	0.01	42.27	-1.70
91	3009	Spain	Navarra: Ribera Arga-Aragón: Villafranca: Badina Escudera	17	n/a	0.01	0.99	n/a	n/a	0.99	0.01	42.27	-1.70
92	3013	Spain	Navarra: Ribera Arga-Aragón: Villafranca: Badina Escudera	17	n/a	0.01	1.00	n/a	n/a	1.00	0.00	42.27	-1.70
93	3014	Spain	Navarra: Ribera Arga-Aragón: Villafranca: Badina Escudera	17	n/a	0.01	0.99	n/a	n/a	1.00	0.00	42.27	-1.70
94	3015	Spain	Navarra: Ribera Arga-Aragón: Villafranca: Badina Escudera	17	n/a	0.01	0.99	n/a	n/a	0.99	0.01	42.27	-1.70
95	3606	Spain	Navarra: Ribera Arga-Aragón: Villafranca: Badina Escudera	17	IIa	0.02	0.98	n/a	n/a	0.99	0.01	42.27	-1.70
96	3607	Spain	Navarra: Ribera Arga-Aragón: Villafranca: Badina Escudera	17	IIa	0.01	1.00	n/a	n/a	1.00	0.01	42.27	-1.70
97	3608	Spain	Navarra: Ribera Arga-Aragón: Villafranca: Badina Escudera	17	IIa	n/a	n/a	n/a	n/a	n/a	n/a	42.27	-1.70
98	3609	Spain	Navarra: Ribera Arga-Aragón: Villafranca: Badina Escudera	17	IIa	n/a	n/a	n/a	n/a	n/a	n/a	42.27	-1.70
99	3610	Spain	Navarra: Ribera Arga-Aragón: Villafranca: Badina Escudera	17	IIa	0.01	1.00	n/a	n/a	1.00	0.00	42.27	-1.70
100	3611	Spain	Navarra: Ribera Arga-Aragón: Villafranca: Badina Escudera	17	IIa	0.01	0.99	n/a	n/a	0.99	0.01	42.27	-1.70
101	3612	Spain	Navarra: Ribera Arga-Aragón: Villafranca: Badina Escudera	17	IIa	0.01	1.00	n/a	n/a	1.00	0.00	42.27	-1.70
102	3613	Spain	Navarra: Ribera Arga-Aragón: Villafranca: Badina Escudera	17	IIa	0.00	1.00	n/a	n/a	1.00	0.00	42.27	-1.70
103	3614	Spain	Navarra: Ribera Arga-Aragón: Villafranca: Badina Escudera	17	IIa	n/a	n/a	n/a	n/a	n/a	n/a	42.27	-1.70
104	3615	Spain	Navarra: Ribera Arga-Aragón: Villafranca: Badina Escudera	17	IIa	0.01	0.99	n/a	n/a	0.99	0.01	42.27	-1.70
105	3616	Spain	Navarra: Ribera Arga-Aragón: Villafranca: Badina Escudera	17	IIa	0.00	1.00	n/a	n/a	1.00	0.00	42.27	-1.70
106	3617	Spain	Navarra: Ribera Arga-Aragón: Villafranca: Badina Escudera	17	IIa	n/a	n/a	n/a	n/a	n/a	n/a	42.27	-1.70
107	3618	Spain	Navarra: Ribera Arga-Aragón: Villafranca: Badina Escudera	17	IIa	0.01	0.99	n/a	n/a	0.99	0.01	42.27	-1.70
108	3619	Spain	Navarra: Ribera Arga-Aragón: Villafranca: Badina Escudera	17	IIa	n/a	n/a	n/a	n/a	n/a	n/a	42.27	-1.70
109	3620	Spain	Navarra: Ribera Arga-Aragón: Villafranca: Badina Escudera	17	IIa	0.00	1.00	n/a	n/a	1.00	0.00	42.27	-1.70
110	3621	Spain	Navarra: Ribera Arga-Aragón: Villafranca: Badina Escudera	17	IIa	0.00	1.00	n/a	n/a	1.00	0.00	42.27	-1.70
111	3622	Spain	Navarra: Ribera Arga-Aragón: Villafranca: Badina Escudera	17	IIa	n/a	n/a	n/a	n/a	n/a	n/a	42.27	-1.70
112	4335	Spain	Navarra: Ribera del Arga-Aragón: Caparroso	18	VIa	0.01	0.99	n/a	n/a	0.99	0.01	42.34	-1.69
113	4336	Spain	Navarra: Ribera del Arga-Aragón: Caparroso	18	VIa	0.01	0.99	n/a	n/a	0.99	0.01	42.34	-1.69
114	4337	Spain	Navarra: Ribera del Arga-Aragón: Caparroso	18	VIa	0.01	0.99	n/a	n/a	0.99	0.01	42.34	-1.69
115	4338	Spain	Navarra: Ribera del Arga-Aragón: Caparroso	18	VIa	0.01	0.99	n/a	n/a	0.98	0.02	42.34	-1.69
116	4339	Spain	Navarra: Ribera del Arga-Aragón: Caparroso	18	IIa	0.01	0.99	n/a	n/a	0.99	0.01	42.34	-1.69
117	4340	Spain	Navarra: Ribera del Arga-Aragón: Caparroso	18	VIa	0.01	1.00	n/a	n/a	1.00	0.01	42.34	-1.69
118	4341	Spain	Navarra: Ribera del Arga-Aragón: Caparroso	18	VIa	0.01	0.99	n/a	n/a	0.99	0.01	42.34	-1.69
119	4342	Spain	Navarra: Ribera del Arga-Aragón: Caparroso	18	IIa	0.00	1.00	n/a	n/a	1.00	0.00	42.34	-1.69
120	15194	Spain	Navarra: Ribera del Arga-Aragón: Caparroso	18	IIa	0.00	1.00	n/a	n/a	1.00	0.00	42.34	-1.69
121	15195	Spain	Navarra: Ribera del Arga-Aragón: Caparroso	18	n/a	0.01	0.99	n/a	n/a	0.99	0.01	42.34	-1.69

#	MTD-T	Country	Locality	Sampling Site	Haplotype Cyt *b*	*K* =2		*K* =2 (subset north)		*K* =2 (subset south)		N	E
■ Table 1 continued						Red	Blue	Grey	Turqouis	Yellow	Purple		
122	15196	Spain	Navarra: Ribera del Arga-Aragón: Caparroso	18	VIa	0.01	0.99	n/a	n/a	1.00	0.01	42.34	-1.69
123	15197	Spain	Navarra: Ribera del Arga-Aragón: Caparroso	18	n/a	0.01	0.99	n/a	n/a	1.00	0.00	42.34	-1.69
124	15198	Spain	Navarra: Ribera del Arga-Aragón: Caparroso	18	n/a	0.01	0.99	n/a	n/a	1.00	0.01	42.34	-1.69
125	15199	Spain	Navarra: Ribera del Arga-Aragón: Caparroso	18	n/a	0.01	0.99	n/a	n/a	0.99	0.01	42.34	-1.69
126	15200	Spain	Navarra: Ribera del Arga-Aragón: Caparroso	18	n/a	0.01	0.99	n/a	n/a	1.00	0.00	42.34	-1.69
127	15201	Spain	Navarra: Ribera del Arga-Aragón: Caparroso	18	IIa	0.00	1.00	n/a	n/a	1.00	0.00	42.34	-1.69
128	15202	Spain	Navarra: Ribera del Arga-Aragón: Caparroso	18	n/a	0.00	1.00	n/a	n/a	1.00	0.00	42.34	-1.69
129	15203	Spain	Navarra: Ribera del Arga-Aragón: Caparroso	18	n/a	0.00	1.00	n/a	n/a	1.00	0.00	42.34	-1.69
130	15204	Spain	Navarra: Ribera del Arga-Aragón: Caparroso	18	VIa	0.01	0.99	n/a	n/a	0.99	0.01	42.34	-1.69
131	15205	Spain	Navarra: Ribera del Arga-Aragón: Caparroso	18	VIa	0.01	1.00	n/a	n/a	1.00	0.00	42.34	-1.69
132	15206	Spain	Navarra: Ribera del Arga-Aragón: Caparroso	18	n/a	0.00	1.00	n/a	n/a	1.00	0.00	42.34	-1.69
133	15207	Spain	Navarra: Ribera del Arga-Aragón: Caparroso	18	n/a	0.01	0.99	n/a	n/a	0.99	0.01	42.34	-1.69
134	15208	Spain	Navarra: Ribera del Arga-Aragón: Caparroso	18	n/a	0.00	1.00	n/a	n/a	1.00	0.00	42.34	-1.69
135	15209	Spain	Navarra: Ribera del Arga-Aragón: Caparroso	18	n/a	0.01	1.00	n/a	n/a	1.00	0.00	42.34	-1.69
136	15210	Spain	Navarra: Ribera del Arga-Aragón: Caparroso	18	VIa	0.01	0.99	n/a	n/a	0.99	0.01	42.34	-1.69
137	7374	Spain	Navarra: Ebro River Basin: Tudela: Barranco Valdelafuente	19	IIa	0.00	1.00	n/a	n/a	1.00	0.00	42.14	-1.67
138	7375	Spain	Navarra: Ebro River Basin: Tudela: Barranco Valdelafuente	19	n/a	0.00	1.00	n/a	n/a	1.00	0.00	42.14	-1.67
139	7376	Spain	Navarra: Ebro River Basin: Tudela: Barranco Valdelafuente	19	n/a	0.00	1.00	n/a	n/a	1.00	0.00	42.14	-1.67
140	7377	Spain	Navarra: Ebro River Basin: Tudela: Barranco Valdelafuente	19	n/a	0.01	0.99	n/a	n/a	1.00	0.00	42.14	-1.67
141	7378	Spain	Navarra: Ebro River Basin: Tudela: Barranco Valdelafuente	19	VIa	0.00	1.00	n/a	n/a	1.00	0.00	42.14	-1.67
142	7379	Spain	Navarra: Ebro River Basin: Tudela: Barranco Valdelafuente	19	n/a	0.00	1.00	n/a	n/a	1.00	0.01	42.14	-1.67
143	7380	Spain	Navarra: Ebro River Basin: Tudela: Barranco Valdelafuente	19	n/a	0.00	1.00	n/a	n/a	1.00	0.00	42.14	-1.67
144	7381	Spain	Navarra: Ebro River Basin: Tudela: Barranco Valdelafuente	19	n/a	0.01	1.00	n/a	n/a	1.00	0.00	42.14	-1.67
145	7382	Spain	Navarra: Ebro River Basin: Tudela: Barranco Valdelafuente	19	n/a	0.01	1.00	n/a	n/a	1.00	0.01	42.14	-1.67
146	7383	Spain	Navarra: Ebro River Basin: Tudela: Barranco Valdelafuente	19	n/a	0.00	1.00	n/a	n/a	1.00	0.00	42.14	-1.67
147	7384	Spain	Navarra: Ebro River Basin: Tudela: Barranco Valdelafuente	19	n/a	0.00	1.00	n/a	n/a	1.00	0.00	42.14	-1.67
148	7385	Spain	Navarra: Ebro River Basin: Tudela: Barranco Valdelafuente	19	n/a	0.00	1.00	n/a	n/a	1.00	0.00	42.14	-1.67
149	7386	Spain	Navarra: Ebro River Basin: Tudela: Barranco Valdelafuente	19	n/a	0.00	1.00	n/a	n/a	1.00	0.00	42.14	-1.67
150	7387	Spain	Navarra: Ebro River Basin: Tudela: Barranco Valdelafuente	19	VIa	0.00	1.00	n/a	n/a	1.00	0.00	42.14	-1.67
151	7388	Spain	Navarra: Ebro River Basin: Tudela: Barranco Valdelafuente	19	n/a	0.00	1.00	n/a	n/a	1.00	0.00	42.14	-1.67
152	7389	Spain	Navarra: Ebro River Basin: Tudela: Barranco Valdelafuente	19	VIa	0.00	1.00	n/a	n/a	1.00	0.00	42.14	-1.67
153	7390	Spain	Navarra: Ebro River Basin: Tudela: Barranco Valdelafuente	19	VIa	0.00	1.00	n/a	n/a	1.00	0.00	42.14	-1.67
154	7391	Spain	Navarra: Ebro River Basin: Tudela: Barranco Valdelafuente	19	n/a	0.00	1.00	n/a	n/a	1.00	0.00	42.14	-1.67
155	7392	Spain	Navarra: Ebro River Basin: Tudela: Barranco Valdelafuente	19	n/a	0.01	0.99	n/a	n/a	1.00	0.01	42.14	-1.67
156	7393	Spain	Navarra: Ebro River Basin: Tudela: Barranco Valdelafuente	19	VIa	0.01	1.00	n/a	n/a	1.00	0.00	42.14	-1.67
157	15310	Spain	Navarra: Ebro River Basin: Tudela: Barranco Valdelafuente	19	n/a	0.00	1.00	n/a	n/a	1.00	0.00	42.14	-1.67
158	15311	Spain	Navarra: Ebro River Basin: Tudela: Barranco Valdelafuente	19	VIa	0.00	1.00	n/a	n/a	1.00	0.00	42.14	-1.67
159	15312	Spain	Navarra: Ebro River Basin: Tudela: Barranco Valdelafuente	19	VIa	0.00	1.00	n/a	n/a	1.00	0.00	42.14	-1.67
160	7432	Spain	Navarra: Tudela: Soto Ramalete	20	VIa	0.00	1.00	n/a	n/a	1.00	0.00	42.16	-1.65
161	7433	Spain	Navarra: Tudela: Soto Ramalete	20	VIa	0.01	0.99	n/a	n/a	0.99	0.01	42.16	-1.65
162	7434	Spain	Navarra: Tudela: Soto Ramalete	20	VIa	0.01	1.00	n/a	n/a	1.00	0.00	42.16	-1.65

#	MTD-T	Country	Locality	Sampling Site	Haplotype Cyt *b*	*K* =2		*K* =2 (subset north)		*K* =2 (subset south)		N	E
■ Table 1 continued						Red	Blue	Grey	Turqouis	Yellow	Purple		
163	7435	Spain	Navarra: Tudela: Soto Ramalete	20	VIa	0.00	1.00	n/a	n/a	1.00	0.00	42.16	-1.65
164	7436	Spain	Navarra: Tudela: Soto Ramalete	20	VIa	0.00	1.00	n/a	n/a	1.00	0.00	42.16	-1.65
165	7437	Spain	Navarra: Tudela: Soto Ramalete	20	VIa	0.00	1.00	n/a	n/a	1.00	0.00	42.16	-1.65
166	7438	Spain	Navarra: Tudela: Soto Ramalete	20	VIa	0.00	1.00	n/a	n/a	1.00	0.00	42.16	-1.65
167	7439	Spain	Navarra: Tudela: Soto Ramalete	20	VIa	0.00	1.00	n/a	n/a	1.00	0.00	42.16	-1.65
168	7440	Spain	Navarra: Tudela: Soto Ramalete	20	VIa	0.01	0.99	n/a	n/a	0.99	0.01	42.16	-1.65
169	7441	Spain	Navarra: Tudela: Soto Ramalete	20	VIa	0.00	1.00	n/a	n/a	1.00	0.00	42.16	-1.65
170	7442	Spain	Navarra: Tudela: Soto Ramalete	20	VIa	0.00	1.00	n/a	n/a	1.00	0.00	42.16	-1.65
171	7443	Spain	Navarra: Tudela: Soto Ramalete	20	VIa	0.01	1.00	n/a	n/a	1.00	0.01	42.16	-1.65
172	7444	Spain	Navarra: Tudela: Soto Ramalete	20	VIa	0.00	1.00	n/a	n/a	1.00	0.00	42.16	-1.65
173	7445	Spain	Navarra: Tudela: Soto Ramalete	20	VIa	0.01	1.00	n/a	n/a	1.00	0.00	42.16	-1.65
174	7446	Spain	Navarra: Tudela: Soto Ramalete	20	VIa	0.00	1.00	n/a	n/a	1.00	0.00	42.16	-1.65
175	7447	Spain	Navarra: Tudela: Soto Ramalete	20	VIa	0.00	1.00	n/a	n/a	1.00	0.00	42.16	-1.65
176	7448	Spain	Navarra: Tudela: Soto Ramalete	20	VIa	0.01	1.00	n/a	n/a	1.00	0.01	42.16	-1.65
177	7449	Spain	Navarra: Tudela: Soto Ramalete	20	VIa	0.00	1.00	n/a	n/a	1.00	0.00	42.16	-1.65
178	7450	Spain	Navarra: Tudela: Soto Ramalete	20	VIa	0.00	1.00	n/a	n/a	1.00	0.00	42.16	-1.65
179	7451	Spain	Navarra: Tudela: Soto Ramalete	20	VIa	0.01	1.00	n/a	n/a	1.00	0.00	42.16	-1.65
180	4343	Spain	Navarra: Ribera Arga-Aragón: Traibuenas	21	IIa	0.01	0.99	n/a	n/a	0.99	0.01	42.37	-1.64
181	7341	Spain	Navarra: Cidacos River Basin: Olite	22	IIa	0.01	0.99	n/a	n/a	0.99	0.01	42.49	-1.63
182	7365	Spain	Navarra: Tudela: Balsas de Tamariz	23	n/a	0.00	1.00	n/a	n/a	1.00	0.00	42.13	-1.62
183	7366	Spain	Navarra: Tudela: Balsas de Tamariz	23	n/a	0.00	1.00	n/a	n/a	1.00	0.01	42.13	-1.62
184	7367	Spain	Navarra: Tudela: Balsas de Tamariz	23	n/a	0.00	1.00	n/a	n/a	1.00	0.00	42.13	-1.62
185	7368	Spain	Navarra: Tudela: Balsas de Tamariz	23	n/a	0.00	1.00	n/a	n/a	1.00	0.00	42.13	-1.62
186	7369	Spain	Navarra: Tudela: Balsas de Tamariz	23	n/a	0.00	1.00	n/a	n/a	1.00	0.00	42.13	-1.62
187	7370	Spain	Navarra: Tudela: Balsas de Tamariz	23	n/a	0.01	0.99	n/a	n/a	1.00	0.01	42.13	-1.62
188	7371	Spain	Navarra: Tudela: Balsas de Tamariz	23	n/a	0.00	1.00	n/a	n/a	1.00	0.00	42.13	-1.62
189	7372	Spain	Navarra: Tudela: Balsas de Tamariz	23	VIa	0.01	0.99	n/a	n/a	0.99	0.01	42.13	-1.62
190	7373	Spain	Navarra: Tudela: Balsas de Tamariz	23	VIa	0.01	0.99	n/a	n/a	0.99	0.01	42.13	-1.62
191	7342	Spain	Navarra: Comarca de Tafalla: Pitillas: Barranco Pozo Pastor	24	VIa	0.01	0.99	n/a	n/a	0.99	0.01	42.44	-1.59
192	7343	Spain	Navarra: Comarca de Tafalla: Pitillas: Barranco Pozo Pastor	24	IIa	0.01	0.99	n/a	n/a	1.00	0.01	42.44	-1.59
193	7344	Spain	Navarra: Comarca de Tafalla: Pitillas: Barranco Pozo Pastor	24	VIa	0.05	0.95	n/a	n/a	0.93	0.07	42.44	-1.59
194	7345	Spain	Navarra: Comarca de Tafalla: Pitillas: Barranco Pozo Pastor	24	VIa	0.01	0.99	n/a	n/a	0.99	0.01	42.44	-1.59
195	7346	Spain	Navarra: Comarca de Tafalla: Pitillas: Barranco Pozo Pastor	24	IIa	0.00	1.00	n/a	n/a	1.00	0.00	42.44	-1.59
196	7347	Spain	Navarra: Comarca de Tafalla: Pitillas: Barranco Pozo Pastor	24	VIa	0.00	1.00	n/a	n/a	1.00	0.00	42.44	-1.59
197	3624	Spain	Navarra: Comarca de Tafalla: Pitillas: Barranco Pozo Pastor	24	VIa	0.01	0.99	n/a	n/a	0.99	0.01	42.44	-1.59
198	3625	Spain	Navarra: Comarca de Tafalla: Pitillas: Barranco Pozo Pastor	24	VIa	0.02	0.98	n/a	n/a	0.98	0.02	42.44	-1.59
199	3626	Spain	Navarra: Comarca de Tafalla: Pitillas: Barranco Pozo Pastor	24	VIa	0.04	0.96	n/a	n/a	0.97	0.03	42.44	-1.59
200	3627	Spain	Navarra: Comarca de Tafalla: Pitillas: Barranco Pozo Pastor	24	VIa	0.01	1.00	n/a	n/a	1.00	0.00	42.44	-1.59
201	3628	Spain	Navarra: Comarca de Tafalla: Pitillas: Barranco Pozo Pastor	24	VIa	0.01	0.99	n/a	n/a	0.99	0.01	42.44	-1.59
202	3629	Spain	Navarra: Comarca de Tafalla: Pitillas: Barranco Pozo Pastor	24	VIa	0.01	0.99	n/a	n/a	0.99	0.01	42.44	-1.59
203	3630	Spain	Navarra: Comarca de Tafalla: Pitillas: Barranco Pozo Pastor	24	VIa	0.01	0.99	n/a	n/a	0.99	0.01	42.44	-1.59

#	MTD-T	Country	Locality	Sampling Site	Haplotype Cyt *b*	*K* =2		*K* =2 (subset north)		*K* =2 (subset south)		N	E
■ Table 1 continued						Red	Blue	Grey	Turqouis	Yellow	Purple		
204	3631	Spain	Navarra: Comarca de Tafalla: Pitillas: Barranco Pozo Pastor	24	VIa	0.01	0.99	n/a	n/a	1.00	0.01	42.44	-1.59
205	3632	Spain	Navarra: Comarca de Tafalla: Pitillas: Barranco Pozo Pastor	24	VIa	0.01	1.00	n/a	n/a	1.00	0.01	42.44	-1.59
206	3633	Spain	Navarra: Comarca de Tafalla: Pitillas: Barranco Pozo Pastor	24	VIa	0.01	0.99	n/a	n/a	0.99	0.01	42.44	-1.59
207	3634	Spain	Navarra: Pitillas: Laguna de Pitillas	24	VIa	0.01	0.99	n/a	n/a	0.99	0.01	42.44	-1.59
208	15104	Spain	Navarra: Comarca de Tafalla: Pitillas: Barranco Pozo Pastor	24	n/a	0.01	0.99	n/a	n/a	1.00	0.00	42.44	-1.59
209	15105	Spain	Navarra: Comarca de Tafalla: Pitillas: Barranco Pozo Pastor	24	VIa	0.18	0.82	n/a	n/a	0.83	0.17	42.44	-1.59
210	7325	Spain	Navarra: Ribera del Arga-Aragón: Rada: Laguna de Rada	25	VIa	0.14	0.86	n/a	n/a	0.94	0.06	42.30	-1.58
211	7326	Spain	Navarra: Ribera del Arga-Aragón: Rada: Laguna de Rada	25	IIa	0.01	0.99	n/a	n/a	0.99	0.01	42.30	-1.58
212	7327	Spain	Navarra: Ribera del Arga-Aragón: Rada: Laguna de Rada	25	IIa	0.01	1.00	n/a	n/a	1.00	0.00	42.30	-1.58
213	7328	Spain	Navarra: Ribera del Arga-Aragón: Rada: Laguna de Rada	25	IIa	0.04	0.96	n/a	n/a	0.99	0.01	42.30	-1.58
214	7329	Spain	Navarra: Ribera del Arga-Aragón: Rada: Laguna de Rada	25	IIa	0.01	0.99	n/a	n/a	1.00	0.00	42.30	-1.58
215	7330	Spain	Navarra: Ribera del Arga-Aragón: Rada: Laguna de Rada	25	IIa	0.01	0.99	n/a	n/a	1.00	0.00	42.30	-1.58
216	7331	Spain	Navarra: Ribera del Arga-Aragón: Rada: Laguna de Rada	25	IIa	0.00	1.00	n/a	n/a	1.00	0.00	42.30	-1.58
217	7332	Spain	Navarra: Ribera del Arga-Aragón: Rada: Laguna de Rada	25	IIa	0.01	0.99	n/a	n/a	0.99	0.01	42.30	-1.58
218	7333	Spain	Navarra: Ribera del Arga-Aragón: Rada: Laguna de Rada	25	IIa	0.00	1.00	n/a	n/a	1.00	0.00	42.30	-1.58
219	7334	Spain	Navarra: Ribera del Arga-Aragón: Rada: Laguna de Rada	25	IIa	0.01	0.99	n/a	n/a	1.00	0.01	42.30	-1.58
220	7335	Spain	Navarra: Ribera del Arga-Aragón: Rada: Laguna de Rada	25	IIa	0.01	0.99	n/a	n/a	1.00	0.00	42.30	-1.58
221	7336	Spain	Navarra: Ribera del Arga-Aragón: Rada: Laguna de Rada	25	IIa	0.01	0.99	n/a	n/a	1.00	0.00	42.30	-1.58
222	7337	Spain	Navarra: Ribera del Arga-Aragón: Rada: Laguna de Rada	25	VIa	0.01	0.99	n/a	n/a	0.99	0.01	42.30	-1.58
223	7338	Spain	Navarra: Ribera del Arga-Aragón: Rada: Laguna de Rada	25	IIa	0.01	1.00	n/a	n/a	1.00	0.00	42.30	-1.58
224	7339	Spain	Navarra: Ribera del Arga-Aragón: Rada: Laguna de Rada	25	IIa	0.00	1.00	n/a	n/a	1.00	0.00	42.30	-1.58
225	3635	Spain	Navarra: Ribera del Arga-Aragón: Rada: Laguna de Rada	25	VIa	0.10	0.91	n/a	n/a	0.95	0.05	42.30	-1.58
226	3636	Spain	Navarra: Ribera del Arga-Aragón: Rada: Laguna de Rada	25	IIa	0.01	0.99	n/a	n/a	0.99	0.01	42.30	-1.58
227	3637	Spain	Navarra: Ribera del Arga-Aragón: Rada: Laguna de Rada	25	VIa	0.01	0.99	n/a	n/a	0.99	0.01	42.30	-1.58
228	3638	Spain	Navarra: Ribera del Arga-Aragón: Rada: Laguna de Rada	25	IIa	0.01	0.99	n/a	n/a	0.99	0.01	42.30	-1.58
229	3639	Spain	Navarra: Ribera del Arga-Aragón: Rada: Laguna de Rada	25	IIa	0.01	0.99	n/a	n/a	1.00	0.01	42.30	-1.58
230	3640	Spain	Navarra: near Rada-Bardenas Reales (unexact origin)	25	IIa	0.01	0.99	n/a	n/a	1.00	0.01	42.30	-1.58
231	7412	Spain	Navarra: La Ribera: Bardenas Reales Natural Park	26	IIa	0.01	0.99	n/a	n/a	1.00	0.01	42.28	-1.58
232	7413	Spain	Navarra: La Ribera: Bardenas Reales Natural Park	26	IIa	0.01	1.00	n/a	n/a	0.99	0.01	42.28	-1.58
233	7414	Spain	Navarra: La Ribera: Bardenas Reales Natural Park	26	IIa	0.00	1.00	n/a	n/a	1.00	0.00	42.28	-1.58
234	7415	Spain	Navarra: La Ribera: Bardenas Reales Natural Park	26	IIa	0.01	0.99	n/a	n/a	1.00	0.01	42.28	-1.58
235	7416	Spain	Navarra: La Ribera: Bardenas Reales Natural Park	26	IIa	0.00	1.00	n/a	n/a	1.00	0.00	42.28	-1.58
236	7417	Spain	Navarra: La Ribera: Bardenas Reales Natural Park	26	IIa	0.01	0.99	n/a	n/a	0.99	0.01	42.28	-1.58
237	7418	Spain	Navarra: La Ribera: Bardenas Reales Natural Park	26	IIa	0.00	1.00	n/a	n/a	1.00	0.00	42.28	-1.58
238	7419	Spain	Navarra: La Ribera: Bardenas Reales Natural Park	26	IIa	0.00	1.00	n/a	n/a	1.00	0.00	42.28	-1.58
239	7420	Spain	Navarra: La Ribera: Bardenas Reales Natural Park	26	IIa	0.00	1.00	n/a	n/a	1.00	0.00	42.28	-1.58
240	7422	Spain	Navarra: La Ribera: Bardenas Reales Natural Park	26	IIa	0.00	1.00	n/a	n/a	1.00	0.00	42.28	-1.58
241	7423	Spain	Navarra: La Ribera: Bardenas Reales Natural Park	26	IIa	0.00	1.00	n/a	n/a	1.00	0.00	42.28	-1.58
242	7424	Spain	Navarra: La Ribera: Bardenas Reales Natural Park	26	IIa	0.00	1.00	n/a	n/a	1.00	0.00	42.28	-1.58
243	7425	Spain	Navarra: La Ribera: Bardenas Reales Natural Park	26	IIa	0.01	0.99	n/a	n/a	0.99	0.01	42.28	-1.58
244	7426	Spain	Navarra: La Ribera: Bardenas Reales Natural Park	26	IIa	0.01	1.00	n/a	n/a	1.00	0.00	42.28	-1.58

#	MTD-T	Country	Locality	Sampling Site	Haplotype Cyt *b*	*K* =2		*K* =2 (subset north)		*K* =2 (subset south)		N	E
■ Table 1 continued						Red	Blue	Grey	Turqouis	Yellow	Purple		
245	7427	Spain	Navarra: La Ribera: Bardenas Reales Natural Park	26	IIa	0.00	1.00	n/a	n/a	1.00	0.00	42.28	-1.58
246	7428	Spain	Navarra: La Ribera: Bardenas Reales Natural Park	26	IIa	0.00	1.00	n/a	n/a	1.00	0.00	42.28	-1.58
247	7429	Spain	Navarra: La Ribera: Bardenas Reales Natural Park	26	IIa	0.01	0.99	n/a	n/a	1.00	0.01	42.28	-1.58
248	7430	Spain	Navarra: La Ribera: Bardenas Reales Natural Park	26	IIa	0.00	1.00	n/a	n/a	1.00	0.00	42.28	-1.58
249	7431	Spain	Navarra: La Ribera: Bardenas Reales Natural Park	26	IIa	0.00	1.00	n/a	n/a	1.00	0.00	42.28	-1.58
250	4349	Spain	Navarra: Ribera del Arga-Aragón: Rada. Soto Recueja	27	IIa	0.01	0.99	n/a	n/a	1.00	0.01	42.32	-1.57
251	4350	Spain	Navarra: Ribera del Arga-Aragón: Rada. Soto Recueja	27	n/a	0.00	1.00	n/a	n/a	1.00	0.00	42.32	-1.57
252	4351	Spain	Navarra: Ribera del Arga-Aragón: Rada. Soto Recueja	27	VIa	0.01	0.99	n/a	n/a	0.99	0.01	42.32	-1.57
253	4352	Spain	Navarra: Ribera del Arga-Aragón: Rada. Soto Recueja	27	VIa	0.09	0.91	n/a	n/a	0.98	0.02	42.32	-1.57
254	4353	Spain	Navarra: Ribera del Arga-Aragón: Rada. Soto Recueja	27	n/a	0.01	0.99	n/a	n/a	1.00	0.00	42.32	-1.57
255	4359	Spain	Navarra: Ribera Arga-Aragón: Santacara	28	IIa	0.01	0.99	n/a	n/a	0.99	0.01	42.37	-1.56
256	4360	Spain	Navarra: Ribera Arga-Aragón: Santacara	28	IIa	0.01	0.99	n/a	n/a	0.99	0.01	42.37	-1.56
257	4361	Spain	Navarra: Ribera Arga-Aragón: Santacara	28	VIa	0.01	0.99	n/a	n/a	1.00	0.01	42.37	-1.56
258	4362	Spain	Navarra: Ribera Arga-Aragón: Santacara	28	IIa	0.00	1.00	n/a	n/a	1.00	0.00	42.37	-1.56
259	4364	Spain	Navarra: Ribera Arga-Aragón: Santacara	28	IIa	0.01	0.99	n/a	n/a	0.99	0.01	42.37	-1.56
260	4365	Spain	Navarra: Ribera Arga-Aragón: Santacara	28	IIa	0.00	1.00	n/a	n/a	1.00	0.00	42.37	-1.56
261	4366	Spain	Navarra: Ribera Arga-Aragón: Santacara	28	IIa	n/a	n/a	n/a	n/a	n/a	n/a	42.37	-1.56
262	4367	Spain	Navarra: Ribera Arga-Aragón: Santacara	28	VIa	n/a	n/a	n/a	n/a	n/a	n/a	42.37	-1.56
263	4370	Spain	Navarra: Ribera Arga-Aragón: Santacara	28	IIa	n/a	n/a	n/a	n/a	n/a	n/a	42.37	-1.56
264	4371	Spain	Navarra: Ribera Arga-Aragón: Santacara	28	VIa	0.02	0.98	n/a	n/a	0.98	0.02	42.37	-1.56
265	4372	Spain	Navarra: Ribera Arga-Aragón: Santacara	28	IIa	0.02	0.98	n/a	n/a	0.99	0.01	42.37	-1.56
266	4373	Spain	Navarra: Ribera Arga-Aragón: Santacara	28	IIa	0.01	0.99	n/a	n/a	1.00	0.01	42.37	-1.56
267	4374	Spain	Navarra: Ribera Arga-Aragón: Santacara	28	IIa	n/a	n/a	n/a	n/a	n/a	n/a	42.37	-1.56
268	4375	Spain	Navarra: Ribera Arga-Aragón: Santacara	28	IIa	n/a	n/a	n/a	n/a	n/a	n/a	42.37	-1.56
269	4376	Spain	Navarra: Ribera Arga-Aragón: Santacara	28	IIa	0.01	0.99	n/a	n/a	1.00	0.00	42.37	-1.56
270	4377	Spain	Navarra: Ribera Arga-Aragón: Santacara	28	VIa	0.00	1.00	n/a	n/a	1.00	0.00	42.37	-1.56
271	4378	Spain	Navarra: Ribera Arga-Aragón: Santacara	28	IIa	0.00	1.00	n/a	n/a	1.00	0.00	42.37	-1.56
272	4379	Spain	Navarra: Ribera Arga-Aragón: Santacara	28	IIa	n/a	n/a	n/a	n/a	n/a	n/a	42.37	-1.56
273	4380	Spain	Navarra: Ribera Arga-Aragón: Santacara	28	IIa	0.00	1.00	n/a	n/a	1.00	0.00	42.37	-1.56
274	4381	Spain	Navarra: Ribera Arga-Aragón: Santacara	28	IIa	0.01	0.99	n/a	n/a	1.00	0.01	42.37	-1.56
275	4382	Spain	Navarra: Ribera Arga-Aragón: Santacara	28	IIa	0.00	1.00	n/a	n/a	1.00	0.00	42.37	-1.56
276	4383	Spain	Navarra: Ribera Arga-Aragón: Santacara	28	IIa	n/a	n/a	n/a	n/a	n/a	n/a	42.37	-1.56
277	4384	Spain	Navarra: Ribera Arga-Aragón: Santacara	28	VIa	n/a	n/a	n/a	n/a	n/a	n/a	42.37	-1.56
278	4385	Spain	Navarra: Ribera Arga-Aragón: Santacara	28	n/a	0.01	0.99	n/a	n/a	1.00	0.01	42.37	-1.56
279	4387	Spain	Navarra: Ribera Arga-Aragón: Santacara	28	n/a	0.01	0.99	n/a	n/a	1.00	0.00	42.37	-1.56
280	4388	Spain	Navarra: Ribera Arga-Aragón: Santacara	28	n/a	0.01	1.00	n/a	n/a	1.00	0.00	42.37	-1.56
281	4390	Spain	Navarra: Ribera Arga-Aragón: Santacara	28	n/a	0.02	0.98	n/a	n/a	0.99	0.01	42.37	-1.56
282	4391	Spain	Navarra: Ribera Arga-Aragón: Santacara	28	n/a	0.01	1.00	n/a	n/a	1.00	0.01	42.37	-1.56
283	7307	Spain	Navarra: Comarca de Tudela: Cabanillas: La Catremana	29	VIa	0.01	1.00	n/a	n/a	1.00	0.00	42.02	-1.55
284	7311	Spain	Navarra: Comarca de Tudela: Cabanillas: La Catremana	29	VIa	0.00	1.00	n/a	n/a	1.00	0.00	42.02	-1.55
285	7312	Spain	Navarra: Comarca de Tudela: Cabanillas: La Catremana	29	VIa	0.01	1.00	n/a	n/a	1.00	0.01	42.02	-1.55

#	MTD-T	Country	Locality	Sampling Site	Haplotype Cyt *b*	*K* =2		*K* =2 (subset north)		*K* =2 (subset south)		N	E
■ Table 1 continued						Red	Blue	Grey	Turqouis	Yellow	Purple		
286	7314	Spain	Navarra: Comarca de Tudela: Cabanillas: La Catremana	29	VIa	0.01	1.00	n/a	n/a	1.00	0.00	42.02	-1.55
287	7315	Spain	Navarra: Comarca de Tudela: Cabanillas: La Catremana	29	VIa	0.00	1.00	n/a	n/a	1.00	0.00	42.02	-1.55
288	4344	Spain	Navarra: Comarca de Tafalla: Ujue	30	IIa	0.01	0.99	n/a	n/a	0.99	0.01	42.47	-1.46
289	4345	Spain	Navarra: Comarca de Tafalla: Ujue	30	VIa	0.01	1.00	n/a	n/a	1.00	0.01	42.47	-1.46
290	4346	Spain	Navarra: Comarca de Tafalla: Ujue	30	IIa	n/a	n/a	n/a	n/a	n/a	n/a	42.47	-1.46
291	4347	Spain	Navarra: Comarca de Tafalla: Ujue	30	VIa	0.01	1.00	n/a	n/a	1.00	0.00	42.47	-1.46
292	4348	Spain	Navarra: Comarca de Tafalla: Ujue	30	VIa	0.05	0.95	n/a	n/a	0.97	0.03	42.47	-1.46
293	15213	Spain	Navarra: Comarca de Tafalla: Ujue	30	n/a	0.01	0.99	n/a	n/a	0.99	0.01	42.47	-1.46
294	15214	Spain	Navarra: Comarca de Tafalla: Ujue	30	IIa	n/a	n/a	n/a	n/a	n/a	n/a	42.47	-1.46
295	15215	Spain	Navarra: Comarca de Tafalla: Ujue	30	VIa	0.01	0.99	n/a	n/a	0.99	0.01	42.47	-1.46
296	15217	Spain	Navarra: Comarca de Tafalla: Ujue	30	n/a	0.01	0.99	n/a	n/a	0.99	0.01	42.47	-1.46
297	15218	Spain	Navarra: Comarca de Tafalla: Ujue	30	n/a	0.01	1.00	n/a	n/a	1.00	0.00	42.47	-1.46
298	15220	Spain	Navarra: Comarca de Tafalla: Ujue	30	n/a	0.01	1.00	n/a	n/a	1.00	0.00	42.47	-1.46
299	15221	Spain	Navarra: Comarca de Tafalla: Ujue	30	n/a	0.05	0.96	n/a	n/a	0.98	0.02	42.47	-1.46
300	15222	Spain	Navarra: Comarca de Tafalla: Ujue	30	n/a	0.01	0.99	n/a	n/a	1.00	0.00	42.47	-1.46
301	15223	Spain	Navarra: Comarca de Tafalla: Ujue	30	VIa	n/a	n/a	n/a	n/a	n/a	n/a	42.47	-1.46
302	15226	Spain	Navarra: Comarca de Tafalla: Ujue	30	VIa	0.01	0.99	n/a	n/a	0.99	0.01	42.47	-1.46
303	15227	Spain	Navarra: Comarca de Tafalla: Ujue	30	n/a	0.01	1.00	n/a	n/a	1.00	0.00	42.47	-1.46
304	15229	Spain	Navarra: Comarca de Tafalla: Ujue	30	n/a	0.01	0.99	n/a	n/a	0.99	0.01	42.47	-1.46
305	15230	Spain	Navarra: Comarca de Tafalla: Ujue	30	n/a	0.00	1.00	n/a	n/a	1.00	0.00	42.47	-1.46
306	15231	Spain	Navarra: Comarca de Tafalla: Ujue	30	n/a	0.01	0.99	n/a	n/a	0.99	0.01	42.47	-1.46
307	15232	Spain	Navarra: Comarca de Tafalla: Ujue	30	IIa	0.01	0.99	n/a	n/a	0.99	0.01	42.47	-1.46
308	15233	Spain	Navarra: Comarca de Tafalla: Ujue	30	n/a	0.01	0.99	n/a	n/a	0.99	0.01	42.47	-1.46
309	15234	Spain	Navarra: Comarca de Tafalla: Ujue	30	n/a	0.00	1.00	n/a	n/a	1.00	0.00	42.47	-1.46
310	15235	Spain	Navarra: Comarca de Tafalla: Ujue	30	n/a	0.01	0.99	n/a	n/a	1.00	0.01	42.47	-1.46
311	7340	Spain	Navarra: Erro River Basin, Mezkiritz	31	VIa	0.03	0.97	n/a	n/a	0.99	0.01	42.97	-1.41
312	14951	Spain	Navarra: Irati River	32	n/a	0.01	0.99	n/a	n/a	1.00	0.01	42.71	-1.33
313	14952	Spain	Navarra: Irati River	32	IIa	0.01	0.99	n/a	n/a	0.99	0.01	42.71	-1.33
314	14953	Spain	Navarra: Irati River	32	n/a	0.01	0.99	n/a	n/a	1.00	0.00	42.71	-1.33
315	14954	Spain	Navarra: Irati River	32	n/a	0.01	0.99	n/a	n/a	0.99	0.01	42.71	-1.33
316	14955	Spain	Navarra: Irati River	32	n/a	0.01	0.99	n/a	n/a	0.98	0.02	42.71	-1.33
317	14956	Spain	Navarra: Irati River	32	IIa	0.01	0.99	n/a	n/a	1.00	0.00	42.71	-1.33
318	14957	Spain	Navarra: Irati River	32	n/a	0.00	1.00	n/a	n/a	1.00	0.01	42.71	-1.33
319	14958	Spain	Navarra: Irati River	32	n/a	0.00	1.00	n/a	n/a	1.00	0.01	42.71	-1.33
320	14959	Spain	Navarra: Irati River	32	n/a	0.01	0.99	n/a	n/a	0.99	0.01	42.71	-1.33
321	14960	Spain	Navarra: Irati River	32	n/a	0.00	1.00	n/a	n/a	1.00	0.00	42.71	-1.33
322	14961	Spain	Navarra: Irati River	32	n/a	0.02	0.99	n/a	n/a	0.99	0.01	42.71	-1.33
323	14962	Spain	Navarra: Irati River	32	n/a	0.01	1.00	n/a	n/a	0.99	0.01	42.71	-1.33
324	14963	Spain	Navarra: Irati River	32	n/a	0.01	0.99	n/a	n/a	0.99	0.01	42.71	-1.33
325	14964	Spain	Navarra: Irati River	32	n/a	0.01	0.99	n/a	n/a	1.00	0.00	42.71	-1.33
326	14965	Spain	Navarra: Irati River	32	n/a	0.01	0.99	n/a	n/a	0.99	0.01	42.71	-1.33

#	MTD-T	Country	Locality	Sampling Site	Haplotype Cyt *b*	*K* =2		*K* =2 (subset north)		*K* =2 (subset south)		N	E
■ Table 1 continued						Red	Blue	Grey	Turqouis	Yellow	Purple		
327	14966	Spain	Navarra: Irati River	32	IIa	0.02	0.98	n/a	n/a	0.99	0.01	42.71	-1.33
328	14967	Spain	Navarra: Irati River	32	n/a	0.00	1.00	n/a	n/a	1.00	0.00	42.71	-1.33
329	14968	Spain	Navarra: Irati River	32	IIa	0.01	1.00	n/a	n/a	1.00	0.00	42.71	-1.33
330	14969	Spain	Navarra: Irati River	32	n/a	0.01	0.99	n/a	n/a	0.99	0.01	42.71	-1.33
331	14970	Spain	Navarra: Irati River	32	n/a	0.01	0.99	n/a	n/a	0.99	0.01	42.71	-1.33
332	14971	Spain	Navarra: Irati River	32	n/a	0.01	0.99	n/a	n/a	0.99	0.01	42.71	-1.33
333	14972	Spain	Navarra: Irati River	32	n/a	0.01	1.00	n/a	n/a	1.00	0.01	42.71	-1.33
334	14973	Spain	Navarra: Irati River	32	IIa	0.01	0.99	n/a	n/a	0.99	0.01	42.71	-1.33
335	15106	Spain	Navarra: Sastoya	33	n/a	0.76	0.24	n/a	n/a	0.70	0.31	42.78	-1.29
336	15107	Spain	Navarra: Sastoya	33	IIa	0.01	0.99	n/a	n/a	1.00	0.01	42.78	-1.29
337	15108	Spain	Navarra: Sastoya	33	IIa	0.12	0.88	n/a	n/a	0.93	0.07	42.78	-1.29
338	15109	Spain	Navarra: Sastoya	33	n/a	0.29	0.71	n/a	n/a	0.99	0.01	42.78	-1.29
339	15110	Spain	Navarra: Sastoya	33	IIa	0.03	0.97	n/a	n/a	0.97	0.03	42.78	-1.29
340	15111	Spain	Navarra: Sastoya	33	n/a	0.01	1.00	n/a	n/a	1.00	0.01	42.78	-1.29
341	14974	Spain	Navarra: Barranco Arielz	34	IIa	0.01	1.00	n/a	n/a	1.00	0.00	42.71	-1.26
342	14975	Spain	Navarra: Barranco Arielz	34	n/a	0.01	0.99	n/a	n/a	1.00	0.01	42.71	-1.26
343	14976	Spain	Navarra: Barranco Arielz	34	n/a	0.01	0.99	n/a	n/a	0.99	0.01	42.71	-1.26
344	14977	Spain	Navarra: Barranco Arielz	34	n/a	0.01	0.99	n/a	n/a	1.00	0.00	42.71	-1.26
345	14978	Spain	Navarra: Barranco Arielz	34	n/a	0.01	0.99	n/a	n/a	1.00	0.00	42.71	-1.26
346	14979	Spain	Navarra: Barranco Arielz	34	n/a	0.02	0.99	n/a	n/a	0.99	0.01	42.71	-1.26
347	14980	Spain	Navarra: Barranco Arielz	34	IIa	0.01	0.99	n/a	n/a	0.99	0.01	42.71	-1.26
348	14981	Spain	Navarra: Barranco Arielz	34	n/a	0.01	0.99	n/a	n/a	1.00	0.01	42.71	-1.26
349	14982	Spain	Navarra: Barranco Arielz	34	IIa	0.01	0.99	n/a	n/a	1.00	0.00	42.71	-1.26
350	14983	Spain	Navarra: Barranco Arielz	34	n/a	0.03	0.97	n/a	n/a	0.99	0.01	42.71	-1.26
351	14984	Spain	Navarra: Barranco Arielz	34	IIa	0.02	0.98	n/a	n/a	1.00	0.01	42.71	-1.26
352	14985	Spain	Navarra: Barranco Arielz	34	n/a	0.01	0.99	n/a	n/a	1.00	0.00	42.71	-1.26
353	14986	Spain	Navarra: Barranco Arielz	34	n/a	0.00	1.00	n/a	n/a	1.00	0.00	42.71	-1.26
354	14987	Spain	Navarra: Barranco Arielz	34	n/a	0.06	0.94	n/a	n/a	0.99	0.02	42.71	-1.26
355	14988	Spain	Navarra: Barranco Arielz	34	IIa	0.01	0.99	n/a	n/a	1.00	0.01	42.71	-1.26
356	14989	Spain	Navarra: Barranco Arielz	34	n/a	0.00	1.00	n/a	n/a	1.00	0.00	42.71	-1.26
357	14990	Spain	Navarra: Barranco Arielz	34	n/a	0.01	0.99	n/a	n/a	1.00	0.00	42.71	-1.26
358	14991	Spain	Navarra: Barranco Arielz	34	n/a	0.01	0.99	n/a	n/a	0.99	0.01	42.71	-1.26
359	14992	Spain	Navarra: Barranco Arielz	34	n/a	0.02	0.98	n/a	n/a	0.99	0.01	42.71	-1.26
360	14993	Spain	Navarra: Barranco Arielz	34	n/a	0.01	0.99	n/a	n/a	1.00	0.00	42.71	-1.26
361	14994	Spain	Navarra: Barranco Arielz	34	n/a	0.01	0.99	n/a	n/a	1.00	0.00	42.71	-1.26
362	14995	Spain	Navarra: Barranco Arielz	34	IIa	0.01	0.99	n/a	n/a	1.00	0.00	42.71	-1.26
363	14996	Spain	Navarra: Barranco Arielz	34	IIa	0.01	0.99	n/a	n/a	0.99	0.01	42.71	-1.26
364	15031	Spain	Navarra: Berroya: Barranco de Peña Blanca	35	IIa	0.01	0.99	n/a	n/a	1.00	0.00	42.71	-1.24
365	15032	Spain	Navarra: Berroya: Barranco de Peña Blanca	35	n/a	0.01	0.99	n/a	n/a	1.00	0.00	42.71	-1.24
366	15034	Spain	Navarra: Berroya: Barranco de Peña Blanca	35	IIa	0.01	0.99	n/a	n/a	1.00	0.00	42.71	-1.24
367	15036	Spain	Navarra: Berroya: Barranco de Peña Blanca	35	IIa	0.01	0.99	n/a	n/a	1.00	0.00	42.71	-1.24

#	MTD-T	Country	Locality	Sampling Site	Haplotype Cyt *b*	*K* =2		*K* =2 (subset north)		*K* =2 (subset south)		N	E
■ Table 1 continued						Red	Blue	Grey	Turqouis	Yellow	Purple		
368	15037	Spain	Navarra: Berroya: Barranco de Peña Blanca	35	IIa	0.00	1.00	n/a	n/a	1.00	0.00	42.71	-1.24
369	15039	Spain	Navarra: Berroya: Barranco de Peña Blanca	35	n/a	0.01	0.99	n/a	n/a	1.00	0.00	42.71	-1.24
370	15040	Spain	Navarra: Berroya: Barranco de Peña Blanca	35	n/a	0.01	1.00	n/a	n/a	1.00	0.00	42.71	-1.24
371	15042	Spain	Navarra: Berroya: Barranco de Peña Blanca	35	n/a	0.00	1.00	n/a	n/a	1.00	0.00	42.71	-1.24
372	15043	Spain	Navarra: Berroya: Barranco de Peña Blanca	35	n/a	0.00	1.00	n/a	n/a	1.00	0.00	42.71	-1.24
373	15044	Spain	Navarra: Berroya: Barranco de Peña Blanca	35	IIa	0.01	1.00	n/a	n/a	1.00	0.00	42.71	-1.24
374	15048	Spain	Navarra: Berroya: Barranco de Peña Blanca	35	n/a	0.01	0.99	n/a	n/a	0.99	0.01	42.71	-1.24
375	15049	Spain	Navarra: Berroya: Barranco de Peña Blanca	35	n/a	0.00	1.00	n/a	n/a	1.00	0.00	42.71	-1.24
376	15050	Spain	Navarra: Berroya: Barranco de Peña Blanca	35	n/a	0.00	1.00	n/a	n/a	1.00	0.00	42.71	-1.24
377	15052	Spain	Navarra: Berroya: Barranco de Peña Blanca	35	n/a	0.00	1.00	n/a	n/a	1.00	0.00	42.71	-1.24
378	15053	Spain	Navarra: Berroya: Barranco de Peña Blanca	35	n/a	0.01	0.99	n/a	n/a	1.00	0.00	42.71	-1.24
379	15054	Spain	Navarra: Berroya: Barranco de Peña Blanca	35	IIa	n/a	n/a	n/a	n/a	n/a	n/a	42.71	-1.24
380	15055	Spain	Navarra: Berroya: Barranco de Peña Blanca	35	n/a	0.01	0.99	n/a	n/a	0.99	0.01	42.71	-1.24
381	15056	Spain	Navarra: Berroya: Barranco de Peña Blanca	35	n/a	0.01	1.00	n/a	n/a	1.00	0.00	42.71	-1.24
382	15060	Spain	Navarra: Berroya: Barranco de Peña Blanca	35	IIa	0.01	0.99	n/a	n/a	1.00	0.00	42.71	-1.24
383	15061	Spain	Navarra: Berroya: Barranco de Peña Blanca	35	IIa	0.01	0.99	n/a	n/a	0.99	0.01	42.71	-1.24
384	15062	Spain	Navarra: Berroya: Barranco de Peña Blanca	35	IIa	n/a	n/a	n/a	n/a	n/a	n/a	42.71	-1.24
385	15064	Spain	Navarra: Berroya: Barranco de Peña Blanca	35	n/a	0.00	1.00	n/a	n/a	1.00	0.00	42.71	-1.24
386	15113	Spain	Aragón: Zaragoza: Erla: Val de Santía	36	VIa	0.01	0.99	n/a	n/a	0.99	0.01	42.12	-1.00
387	15114	Spain	Aragón: Zaragoza: Erla: Val de Santía	36	VIa	0.02	0.98	n/a	n/a	0.98	0.02	42.12	-1.00
388	15115	Spain	Aragón: Zaragoza: Erla: Val de Santía	36	n/a	0.08	0.92	n/a	n/a	0.85	0.15	42.12	-1.00
389	15116	Spain	Aragón: Zaragoza: Erla: Val de Santía	36	n/a	0.02	0.98	n/a	n/a	0.99	0.01	42.12	-1.00
390	15117	Spain	Aragón: Zaragoza: Erla: Val de Santía	36	VIa	0.28	0.73	n/a	n/a	0.71	0.29	42.12	-1.00
391	15118	Spain	Aragón: Zaragoza: Erla: Val de Santía	36	n/a	0.01	0.99	n/a	n/a	0.99	0.01	42.12	-1.00
392	15119	Spain	Aragón: Zaragoza: Erla: Val de Santía	36	VIa	0.01	0.99	n/a	n/a	1.00	0.01	42.12	-1.00
393	15120	Spain	Aragón: Zaragoza: Erla: Val de Santía	36	n/a	0.04	0.96	n/a	n/a	0.95	0.05	42.12	-1.00
394	15121	Spain	Aragón: Zaragoza: Erla: Val de Santía	36	VIa	0.02	0.98	n/a	n/a	0.98	0.02	42.12	-1.00
395	15122	Spain	Aragón: Zaragoza: Erla: Val de Santía	36	n/a	0.01	0.99	n/a	n/a	0.99	0.01	42.12	-1.00
396	15123	Spain	Aragón: Zaragoza: Erla: Val de Santía	36	n/a	0.02	0.98	n/a	n/a	0.97	0.03	42.12	-1.00
397	15124	Spain	Aragón: Zaragoza: Erla: Val de Santía	36	n/a	0.01	0.99	n/a	n/a	0.99	0.01	42.12	-1.00
398	7348	Spain	Zaragoza: Gallego River Basin: Zuera (Salinas)	37	VIa	0.02	0.98	n/a	n/a	0.99	0.01	41.90	-0.77
399	7349	Spain	Zaragoza: Gallego River Basin: Zuera (Salinas)	37	VIa	0.02	0.98	n/a	n/a	0.99	0.01	41.90	-0.77
400	7350	Spain	Zaragoza: Gallego River Basin: Zuera (Salinas)	37	VIa	0.03	0.97	n/a	n/a	0.98	0.02	41.90	-0.77
401	7351	Spain	Zaragoza: Gallego River Basin: Zuera (Salinas)	37	VIa	0.02	0.98	n/a	n/a	0.98	0.02	41.90	-0.77
402	7352	Spain	Zaragoza: Gallego River Basin: Zuera (Salinas)	37	VIa	0.01	0.99	n/a	n/a	0.99	0.01	41.90	-0.77
403	7353	Spain	Zaragoza: Gallego River Basin: Zuera (Salinas)	37	VIa	0.03	0.98	n/a	n/a	0.98	0.02	41.90	-0.77
404	7354	Spain	Zaragoza: Gallego River Basin: Zuera (Salinas)	37	VIa	0.02	0.98	n/a	n/a	0.98	0.02	41.90	-0.77
405	7355	Spain	Zaragoza: Gallego River Basin: Zuera (Salinas)	37	VIa	0.06	0.94	n/a	n/a	0.95	0.05	41.90	-0.77
406	7356	Spain	Zaragoza: Gallego River Basin: Zuera (Salinas)	37	VIa	0.01	0.99	n/a	n/a	1.00	0.01	41.90	-0.77
407	7357	Spain	Zaragoza: Gallego River Basin: Zuera (Salinas)	37	VIa	0.01	0.99	n/a	n/a	0.99	0.01	41.90	-0.77
408	7358	Spain	Zaragoza: Gallego River Basin: Zuera (Salinas)	37	n/a	0.06	0.94	n/a	n/a	0.95	0.05	41.90	-0.77

#	MTD-T	Country	Locality	Sampling Site	Haplotype Cyt *b*	*K* =2		*K* =2 (subset north)		*K* =2 (subset south)		N	E
■ Table 1 continued						Red	Blue	Grey	Turqouis	Yellow	Purple		
409	7359	Spain	Zaragoza: Gallego River Basin: Zuera (Salinas)	37	VIa	0.01	1.00	n/a	n/a	0.99	0.01	41.90	-0.77
410	7360	Spain	Zaragoza: Gallego River Basin: Zuera (Salinas)	37	VIa	0.02	0.98	n/a	n/a	0.99	0.02	41.90	-0.77
411	7361	Spain	Zaragoza: Gallego River Basin: Zuera (Salinas)	37	VIa	0.02	0.99	n/a	n/a	0.98	0.02	41.90	-0.77
412	7362	Spain	Zaragoza: Gallego River Basin: Zuera (Salinas)	37	VIa	0.01	0.99	n/a	n/a	0.99	0.01	41.90	-0.77
413	7363	Spain	Zaragoza: Gallego River Basin: Zuera (Salinas)	37	VIa	0.01	0.99	n/a	n/a	0.99	0.01	41.90	-0.77
414	7364	Spain	Zaragoza: Gallego River Basin: Zuera (Salinas)	37	VIa	0.02	0.98	n/a	n/a	0.99	0.01	41.90	-0.77
415	15125	Spain	Zaragoza: Gallego River Basin: Zuera (Salinas)	37	VIa	0.01	0.99	n/a	n/a	1.00	0.01	41.90	-0.77
416	15126	Spain	Zaragoza: Gallego River Basin: Zuera (Salinas)	37	VIa	0.01	0.99	n/a	n/a	0.99	0.01	41.90	-0.77
417	15127	Spain	Zaragoza: Gallego River Basin: Zuera (Salinas)	37	VIa	0.02	0.98	n/a	n/a	0.99	0.01	41.90	-0.77
418	14997	Spain	Aragón: Huesca: Sotonera: Le Bassin D'Alboré	38	IIa	0.04	0.96	n/a	n/a	0.90	0.10	42.15	-0.70
419	14999	Spain	Aragón: Huesca: Sotonera: Le Bassin D'Alboré	38	n/a	0.01	0.99	n/a	n/a	0.98	0.02	42.15	-0.70
420	15001	Spain	Aragón: Huesca: Sotonera: Le Bassin D'Alboré	38	n/a	0.01	0.99	n/a	n/a	1.00	0.01	42.15	-0.70
421	15002	Spain	Aragón: Huesca: Sotonera: Le Bassin D'Alboré	38	IIa	0.01	0.99	n/a	n/a	0.98	0.02	42.15	-0.70
422	15004	Spain	Aragón: Huesca: Sotonera: Le Bassin D'Alboré	38	n/a	0.05	0.95	n/a	n/a	0.95	0.05	42.15	-0.70
423	15005	Spain	Aragón: Huesca: Sotonera: Le Bassin D'Alboré	38	IIa	0.01	0.99	n/a	n/a	0.99	0.01	42.15	-0.70
424	15006	Spain	Aragón: Huesca: Sotonera: Le Bassin D'Alboré	38	n/a	0.01	0.99	n/a	n/a	0.99	0.01	42.15	-0.70
425	15007	Spain	Aragón: Huesca: Sotonera: Le Bassin D'Alboré	38	IIa	n/a	n/a	n/a	n/a	n/a	n/a	42.15	-0.70
426	15009	Spain	Aragón: Huesca: Sotonera: Le Bassin D'Alboré	38	IIa	0.01	0.99	n/a	n/a	0.99	0.01	42.15	-0.70
427	15012	Spain	Aragón: Huesca: Sotonera: Le Bassin D'Alboré	38	IIa	0.07	0.93	n/a	n/a	0.98	0.02	42.15	-0.70
428	15014	Spain	Aragón: Huesca: Sotonera: Le Bassin D'Alboré	38	n/a	0.01	0.99	n/a	n/a	0.99	0.01	42.15	-0.70
429	15015	Spain	Aragón: Huesca: Sotonera: Le Bassin D'Alboré	38	n/a	0.01	0.99	n/a	n/a	0.99	0.01	42.15	-0.70
430	15017	Spain	Aragón: Huesca: Sotonera: Le Bassin D'Alboré	38	IIa	0.01	0.99	n/a	n/a	0.99	0.01	42.15	-0.70
431	15018	Spain	Aragón: Huesca: Sotonera: Le Bassin D'Alboré	38	n/a	0.01	1.00	n/a	n/a	0.99	0.01	42.15	-0.70
432	15019	Spain	Aragón: Huesca: Sotonera: Le Bassin D'Alboré	38	n/a	0.01	0.99	n/a	n/a	0.99	0.01	42.15	-0.70
433	15020	Spain	Aragón: Huesca: Sotonera: Le Bassin D'Alboré	38	IIa	0.51	0.49	n/a	n/a	0.64	0.36	42.15	-0.70
434	15021	Spain	Aragón: Huesca: Sotonera: Le Bassin D'Alboré	38	n/a	0.20	0.81	n/a	n/a	0.79	0.21	42.15	-0.70
435	15022	Spain	Aragón: Huesca: Sotonera: Le Bassin D'Alboré	38	n/a	0.01	0.99	n/a	n/a	0.99	0.01	42.15	-0.70
436	15023	Spain	Aragón: Huesca: Sotonera: Le Bassin D'Alboré	38	IIa	n/a	n/a	n/a	n/a	n/a	n/a	42.15	-0.70
437	15026	Spain	Aragón: Huesca: Sotonera: Le Bassin D'Alboré	38	n/a	0.02	0.98	n/a	n/a	0.99	0.01	42.15	-0.70
438	15027	Spain	Aragón: Huesca: Sotonera: Le Bassin D'Alboré	38	n/a	0.01	0.99	n/a	n/a	0.99	0.01	42.15	-0.70
439	15029	Spain	Aragón: Huesca: Sotonera: Le Bassin D'Alboré	38	n/a	0.01	0.99	n/a	n/a	0.98	0.02	42.15	-0.70
440	14934	Spain	Zaragoza: Pina de Ebro	39	n/a	0.07	0.93	n/a	n/a	0.94	0.06	41.48	-0.56
441	14935	Spain	Zaragoza: Pina de Ebro	39	n/a	0.26	0.74	n/a	n/a	0.83	0.17	41.48	-0.56
442	14936	Spain	Zaragoza: Pina de Ebro	39	VIa	0.23	0.78	n/a	n/a	0.72	0.28	41.48	-0.56
443	14937	Spain	Zaragoza: Pina de Ebro	39	n/a	0.41	0.59	n/a	n/a	0.75	0.25	41.48	-0.56
444	14938	Spain	Zaragoza: Pina de Ebro	39	VIa	0.01	0.99	n/a	n/a	0.99	0.01	41.48	-0.56
445	14939	Spain	Zaragoza: Pina de Ebro	39	VIa	0.06	0.94	n/a	n/a	0.99	0.01	41.48	-0.56
446	14940	Spain	Zaragoza: Pina de Ebro	39	VIa	0.07	0.94	n/a	n/a	0.97	0.03	41.48	-0.56
447	14941	Spain	Zaragoza: Pina de Ebro	39	VIa	0.03	0.97	n/a	n/a	0.98	0.02	41.48	-0.56
448	8043	Spain	Valencia: Valencia: Tancat de la Pipa	40	VIa	0.42	0.58	n/a	n/a	0.80	0.21	39.37	-0.35

#	MTD-T	Country	Locality	Sampling Site	Haplotype Cyt *b*	*K* =2		*K* =2 (subset north)		*K* =2 (subset south)		N	E
■ Table 1 continued						Red	Blue	Grey	Turqouis	Yellow	Purple		
449	7318	Spain	Huesca: Cinca Medio: Binaced: Barranco de la Mora (Cinca River Basin)	41	VIa	0.01	0.99	n/a	n/a	1.00	0.01	41.83	0.16
450	7319	Spain	Huesca: Cinca Medio: Binaced: Barranco de la Mora (Cinca River Basin)	41	VIa	0.01	0.99	n/a	n/a	0.99	0.01	41.83	0.16
451	7320	Spain	Huesca: Cinca Medio: Binaced: Barranco de la Mora (Cinca River Basin)	41	VIa	0.02	0.98	n/a	n/a	0.99	0.01	41.83	0.16
452	7321	Spain	Huesca: Cinca Medio: Binaced: Barranco de la Mora (Cinca River Basin)	41	VIa	0.03	0.97	n/a	n/a	0.99	0.01	41.83	0.16
453	7322	Spain	Huesca: Cinca Medio: Binaced: Barranco de la Mora (Cinca River Basin)	41	VIa	0.29	0.71	n/a	n/a	0.99	0.01	41.83	0.16
454	8061	Spain	Valencia: Castelló: Plana Alta: Torreblanca: Marjales de Torreblanca	42	Va	0.13	0.87	n/a	n/a	0.87	0.13	40.19	0.20
455	8062	Spain	Valencia: Castelló: Plana Alta: Torreblanca: Marjales de Torreblanca	42	Va	0.10	0.90	n/a	n/a	0.92	0.08	40.19	0.20
456	8063	Spain	Valencia: Castelló: Plana Alta: Torreblanca: Marjales de Torreblanca	42	Va	0.02	0.98	n/a	n/a	0.98	0.02	40.19	0.20
457	8064	Spain	Valencia: Castelló: Plana Alta: Torreblanca: Marjales de Torreblanca	42	Va	0.01	0.99	n/a	n/a	0.99	0.01	40.19	0.20
458	8065	Spain	Valencia: Castelló: Plana Alta: Torreblanca: Marjales de Torreblanca	42	Va	0.01	0.99	n/a	n/a	0.99	0.01	40.19	0.20
459	8066	Spain	Valencia: Castelló: Plana Alta: Torreblanca: Marjales de Torreblanca	42	Va	0.44	0.56	n/a	n/a	0.54	0.46	40.19	0.20
460	8068	Spain	Valencia: Castelló: Plana Alta: Torreblanca: Marjales de Torreblanca	42	Va	0.01	0.99	n/a	n/a	1.00	0.01	40.19	0.20
461	8072	Spain	Valencia: Castelló: Plana Alta: Torreblanca: Marjales de Torreblanca	42	Va	0.01	0.99	n/a	n/a	0.98	0.02	40.19	0.20
462	8073	Spain	Valencia: Castelló: Plana Alta: Torreblanca: Marjales de Torreblanca	42	Va	0.35	0.65	n/a	n/a	0.69	0.31	40.19	0.20
463	8075	Spain	Valencia: Castelló: Plana Alta: Torreblanca: Marjales de Torreblanca	42	Va	0.04	0.96	n/a	n/a	0.97	0.03	40.19	0.20
464	8077	Spain	Valencia: Castelló: Plana Alta: Torreblanca: Marjales de Torreblanca	42	VIa	0.29	0.71	n/a	n/a	0.67	0.33	40.19	0.20
465	8078	Spain	Valencia: Castelló: Plana Alta: Torreblanca: Marjales de Torreblanca	42	VIa	0.04	0.97	n/a	n/a	0.94	0.06	40.19	0.20
466	8079	Spain	Valencia: Castelló: Plana Alta: Torreblanca: Marjales de Torreblanca	42	VIa	0.43	0.57	n/a	n/a	0.62	0.38	40.19	0.20
467	8080	Spain	Valencia: Castelló: Plana Alta: Torreblanca: Marjales de Torreblanca	42	VIa	0.40	0.60	n/a	n/a	0.62	0.38	40.19	0.20
468	8081	Spain	Valencia: Castelló: Plana Alta: Torreblanca: Marjales de Torreblanca	42	Va	0.04	0.96	n/a	n/a	0.93	0.07	40.19	0.20

#	MTD-T	Country	Locality	Sampling Site	Haplotype Cyt *b*	*K* =2		*K* =2 (subset north)		*K* =2 (subset south)		N	E
■ Table 1 continued						Red	Blue	Grey	Turqouis	Yellow	Purple		
469	8082	Spain	Valencia: Castelló: Plana Alta: Torreblanca: Marjales de Torreblanca	42	Va	0.12	0.88	n/a	n/a	0.84	0.16	40.19	0.20
470	8083	Spain	Valencia: Castelló: Plana Alta: Torreblanca: Marjales de Torreblanca	42	VIa	0.10	0.90	n/a	n/a	0.96	0.04	40.19	0.20
471	8084	Spain	Valencia: Castelló: Plana Alta: Torreblanca: Marjales de Torreblanca	42	n/a	0.12	0.88	n/a	n/a	0.82	0.18	40.19	0.20
472	8085	Spain	Valencia: Castelló: Plana Alta: Torreblanca: Marjales de Torreblanca	42	Va	0.04	0.96	n/a	n/a	0.96	0.04	40.19	0.20
473	8086	Spain	Valencia: Castelló: Plana Alta: Torreblanca: Marjales de Torreblanca	42	n/a	0.01	0.99	n/a	n/a	0.98	0.02	40.19	0.20
474	7486	Spain	Aragón: Huesca: Ribagorza Oriental: Estaña (Lakes)	43	IIa	0.05	0.95	n/a	n/a	0.99	0.01	42.03	0.53
475	7487	Spain	Aragón: Huesca: Ribagorza Oriental: Estaña (Lakes)	43	IIa	0.04	0.96	n/a	n/a	0.99	0.01	42.03	0.53
476	7488	Spain	Aragón: Huesca: Ribagorza Oriental: Estaña (Lakes)	43	IIa	0.03	0.97	n/a	n/a	0.99	0.01	42.03	0.53
477	7489	Spain	Aragón: Huesca: Ribagorza Oriental: Estaña (Lakes)	43	IIa	n/a	n/a	n/a	n/a	n/a	n/a	42.03	0.53
478	7490	Spain	Aragón: Huesca: Ribagorza Oriental: Estaña (Lakes)	43	IIa	0.03	0.98	n/a	n/a	0.99	0.01	42.03	0.53
479	7491	Spain	Aragón: Huesca: Ribagorza Oriental: Estaña (Lakes)	43	IIa	0.08	0.92	n/a	n/a	0.98	0.02	42.03	0.53
480	7492	Spain	Aragón: Huesca: Ribagorza Oriental: Estaña (Lakes)	43	IIa	n/a	n/a	n/a	n/a	n/a	n/a	42.03	0.53
481	7493	Spain	Aragón: Huesca: Ribagorza Oriental: Estaña (Lakes)	43	IIa	n/a	n/a	n/a	n/a	n/a	n/a	42.03	0.53
482	7494	Spain	Aragón: Huesca: Ribagorza Oriental: Estaña (Lakes)	43	IIa	n/a	n/a	n/a	n/a	n/a	n/a	42.03	0.53
483	7495	Spain	Aragón: Huesca: Ribagorza Oriental: Estaña (Lakes)	43	IIa	n/a	n/a	n/a	n/a	n/a	n/a	42.03	0.53
484	7496	Spain	Aragón: Huesca: Ribagorza Oriental: Estaña (Lakes)	43	IIa	0.02	0.98	n/a	n/a	0.99	0.01	42.03	0.53
485	10632	Spain	Aragón: Huesca: Ribagorza Oriental: Estaña (Lakes)	43	n/a	0.03	0.98	n/a	n/a	0.99	0.01	42.03	0.53
486	10982	Spain	Aragón: Huesca: Ribagorza Oriental: Estaña (Lakes)	43	IIa	n/a	n/a	n/a	n/a	n/a	n/a	42.03	0.53
487	10983	Spain	Aragón: Huesca: Ribagorza Oriental: Estaña (Lakes)	43	n/a	0.03	0.97	n/a	n/a	0.99	0.01	42.03	0.53
488	10984	Spain	Aragón: Huesca: Ribagorza Oriental: Estaña (Lakes)	43	IIa	0.05	0.95	n/a	n/a	0.98	0.02	42.03	0.53
489	10986	Spain	Aragón: Huesca: Ribagorza Oriental: Estaña (Lakes)	43	IIa	0.03	0.98	n/a	n/a	0.99	0.01	42.03	0.53
490	10987	Spain	Aragón: Huesca: Ribagorza Oriental: Estaña (Lakes)	43	IIa	n/a	n/a	n/a	n/a	n/a	n/a	42.03	0.53
491	10988	Spain	Aragón: Huesca: Ribagorza Oriental: Estaña (Lakes)	43	IIa	n/a	n/a	n/a	n/a	n/a	n/a	42.03	0.53
492	10989	Spain	Aragón: Huesca: Ribagorza Oriental: Estaña (Lakes)	43	IIa	n/a	n/a	n/a	n/a	n/a	n/a	42.03	0.53
493	10990	Spain	Aragón: Huesca: Ribagorza Oriental: Estaña (Lakes)	43	IIa	0.05	0.95	n/a	n/a	0.98	0.02	42.03	0.53
494	10991	Spain	Aragón: Huesca: Ribagorza Oriental: Estaña (Lakes)	43	IIa	0.04	0.96	n/a	n/a	1.00	0.00	42.03	0.53
495	8036	Spain	Aragón: Huesca: Ribagorza Oriental: Estaña (Lakes)	43	VId	0.00	1.00	n/a	n/a	1.00	0.00	42.03	0.53
496	8039	Spain	Aragón: Huesca: Ribagorza Oriental: Estaña (Lakes)	43	IIa	0.01	0.99	n/a	n/a	0.99	0.01	42.03	0.53
497	8041	Spain	Aragón: Huesca: Ribagorza Oriental: Estaña (Lakes)	43	IIa	n/a	n/a	n/a	n/a	n/a	n/a	42.03	0.53
498	8042	Spain	Aragón: Huesca: Ribagorza Oriental: Estaña (Lakes)	43	IIa	0.38	0.62	n/a	n/a	0.98	0.02	42.03	0.53
499	15160	Spain	Aragón: Huesca: Ribagorza Oriental: Estaña (Lakes)	43	IIa	n/a	n/a	n/a	n/a	n/a	n/a	42.03	0.53
500	15161	Spain	Aragón: Huesca: Ribagorza Oriental: Estaña (Lakes)	43	IIa	n/a	n/a	n/a	n/a	n/a	n/a	42.03	0.53
501	15168	Spain	Aragón: Huesca: Ribagorza Oriental: Estaña (Lakes)	43	VId	0.00	1.00	n/a	n/a	1.00	0.00	42.03	0.53
502	15170	Spain	Aragón: Huesca: Ribagorza Oriental: Estaña (Lakes)	43	IIa	0.11	0.89	n/a	n/a	0.99	0.01	42.03	0.53
503	15171	Spain	Aragón: Huesca: Ribagorza Oriental: Estaña (Lakes)	43	n/a	0.01	0.99	n/a	n/a	1.00	0.00	42.03	0.53
504	15175	Spain	Aragón: Huesca: Ribagorza Oriental: Estaña (Lakes)	43	IIa	0.01	0.99	n/a	n/a	1.00	0.01	42.03	0.53

#	MTD-T	Country	Locality	Sampling Site	Haplotype Cyt *b*	*K* =2		*K* =2 (subset north)		*K* =2 (subset south)		N	E
■ Table 1 continued						Red	Blue	Grey	Turqouis	Yellow	Purple		
505	15176	Spain	Aragón: Huesca: Ribagorza Oriental: Estaña (Lakes)	43	n/a	0.04	0.96	n/a	n/a	0.99	0.01	42.03	0.53
506	8016	Spain	Catalonia: Tarragona: Ebro Delta	44	Va	0.46	0.54	n/a	n/a	0.87	0.13	40.65	0.65
507	8017	Spain	Catalonia: Tarragona: Ebro Delta	44	Va	0.01	0.99	n/a	n/a	1.00	0.00	40.65	0.65
508	8018	Spain	Catalonia: Tarragona: Ebro Delta	44	Va	0.06	0.94	n/a	n/a	0.98	0.02	40.65	0.65
509	8019	Spain	Catalonia: Tarragona: Ebro Delta	44	Va	0.04	0.97	n/a	n/a	0.97	0.03	40.65	0.65
510	8020	Spain	Catalonia: Tarragona: Ebro Delta	44	IIa	0.98	0.02	n/a	n/a	0.48	0.52	40.65	0.65
511	8021	Spain	Catalonia: Tarragona: Ebro Delta	44	Va	0.02	0.98	n/a	n/a	0.98	0.03	40.65	0.65
512	8022	Spain	Catalonia: Tarragona: Ebro Delta	44	Va	0.15	0.86	n/a	n/a	0.89	0.11	40.65	0.65
513	8023	Spain	Catalonia: Tarragona: Ebro Delta	44	VIa	0.25	0.75	n/a	n/a	0.85	0.15	40.65	0.65
514	8024	Spain	Catalonia: Tarragona: Ebro Delta	44	VIa	0.00	1.00	n/a	n/a	1.00	0.00	40.65	0.65
515	8025	Spain	Catalonia: Tarragona: Ebro Delta	44	n/a	0.01	0.99	n/a	n/a	0.98	0.02	40.65	0.65
516	8026	Spain	Catalonia: Tarragona: Ebro Delta	44	Va	0.19	0.81	n/a	n/a	0.84	0.17	40.65	0.65
517	8028	Spain	Catalonia: Tarragona: Ebro Delta	44	VId	0.00	1.00	n/a	n/a	1.00	0.00	40.65	0.65
518	8029	Spain	Catalonia: Tarragona: Ebro Delta	44	VIa	0.00	1.00	n/a	n/a	1.00	0.01	40.65	0.65
519	8030	Spain	Catalonia: Tarragona: Ebro Delta	44	Va	0.04	0.96	n/a	n/a	0.99	0.01	40.65	0.65
520	8031	Spain	Catalonia: Tarragona: Ebro Delta	44	VId	0.00	1.00	n/a	n/a	1.00	0.00	40.65	0.65
521	8032	Spain	Catalonia: Tarragona: Ebro Delta	44	Va	0.18	0.82	n/a	n/a	0.72	0.28	40.65	0.65
522	8033	Spain	Catalonia: Tarragona: Ebro Delta	44	Va	0.16	0.84	n/a	n/a	0.85	0.15	40.65	0.65
523	10297	Spain	Catalonia: Tarragona: Tarragones: Vila-Seca: Sequia Major	45	n/a	0.99	0.01	n/a	n/a	0.01	0.99	41.08	1.18
524	10298	Spain	Catalonia: Tarragona: Tarragones: Vila-Seca: Sequia Major	45	Va	0.99	0.01	n/a	n/a	0.01	0.99	41.08	1.18
525	10299	Spain	Catalonia: Tarragona: Tarragones: Vila-Seca: Sequia Major	45	Va	0.99	0.01	n/a	n/a	0.01	0.99	41.08	1.18
526	10300	Spain	Catalonia: Tarragona: Tarragones: Vila-Seca: Sequia Major	45	Va	1.00	0.00	n/a	n/a	0.00	1.00	41.08	1.18
527	10301	Spain	Catalonia: Tarragona: Tarragones: Vila-Seca: Sequia Major	45	Va	1.00	0.00	n/a	n/a	0.00	1.00	41.08	1.18
528	10302	Spain	Catalonia: Tarragona: Tarragones: Vila-Seca: Sequia Major	45	Va	1.00	0.00	n/a	n/a	0.00	1.00	41.08	1.18
529	10303	Spain	Catalonia: Tarragona: Tarragones: Vila-Seca: Sequia Major	45	Va	0.99	0.01	n/a	n/a	0.01	0.99	41.08	1.18
530	10304	Spain	Catalonia: Tarragona: Tarragones: Vila-Seca: Sequia Major	45	Va	0.99	0.01	n/a	n/a	0.01	0.99	41.08	1.18
531	10305	Spain	Catalonia: Tarragona: Tarragones: Vila-Seca: Sequia Major	45	Va	1.00	0.01	n/a	n/a	0.02	0.98	41.08	1.18
532	10306	Spain	Catalonia: Tarragona: Tarragones: Vila-Seca: Sequia Major	45	Va	1.00	0.01	n/a	n/a	0.00	1.00	41.08	1.18
533	10307	Spain	Catalonia: Tarragona: Tarragones: Vila-Seca: Sequia Major	45	Va	1.00	0.00	n/a	n/a	0.00	1.00	41.08	1.18
534	10308	Spain	Catalonia: Tarragona: Tarragones: Vila-Seca: Sequia Major	45	Va	1.00	0.00	n/a	n/a	0.01	0.99	41.08	1.18
535	10309	Spain	Catalonia: Tarragona: Tarragones: Vila-Seca: Sequia Major	45	Va	0.99	0.01	n/a	n/a	0.01	0.99	41.08	1.18
536	10310	Spain	Catalonia: Tarragona: Tarragones: Vila-Seca: Sequia Major	45	Va	1.00	0.01	n/a	n/a	0.01	1.00	41.08	1.18
537	10311	Spain	Catalonia: Tarragona: Tarragones: Vila-Seca: Sequia Major	45	Va	0.99	0.01	n/a	n/a	0.01	0.99	41.08	1.18
538	8053	Spain	Catalonia: Tarragona: Tarragones: Vila-Seca: Sequia Major	45	n/a	0.99	0.01	n/a	n/a	0.01	0.99	41.08	1.18
539	8054	Spain	Catalonia: Tarragona: Tarragones: Vila-Seca: Sequia Major	45	Va	0.99	0.01	n/a	n/a	0.01	0.99	41.08	1.18
540	8056	Spain	Catalonia: Tarragona: Tarragones: Vila-Seca: Sequia Major	45	Va	0.99	0.01	n/a	n/a	0.01	1.00	41.08	1.18
541	8057	Spain	Catalonia: Tarragona: Tarragones: Vila-Seca: Sequia Major	45	Va	1.00	0.00	n/a	n/a	0.00	1.00	41.08	1.18
542	8058	Spain	Catalonia: Tarragona: Tarragones: Vila-Seca: Sequia Major	45	Va	1.00	0.00	n/a	n/a	0.00	1.00	41.08	1.18
543	5867	Spain	Catalonia: Barcelona: Alt Penedès: Castellet i La Gornal: Foix Reservoir	46	VIa	0.01	0.99	n/a	n/a	0.99	0.01	41.26	1.65
544	5690	Spain	Catalonia: Barcelona: Valles Oriental: Llica d'Amunt	47	Va	0.99	0.01	n/a	n/a	0.04	0.96	41.61	2.24

#	MTD-T	Country	Locality	Sampling Site	Haplotype Cyt *b*	*K* =2		*K* =2 (subset north)		*K* =2 (subset south)		N	E
■ Table 1 continued						Red	Blue	Grey	Turqouis	Yellow	Purple		
545	11050	Spain	Catalonia: Girona: La Garrotxa: Olot	48	VId	0.00	1.00	n/a	n/a	1.00	0.00	42.18	2.49
546	5693	Spain	Catalonia: Girona: La Selva: Osor	49	IIa	1.00	0.00	n/a	n/a	0.01	1.00	41.95	2.56
547	10219	Spain	Catalonia: Girona: La Selva: Riudarenes	50	IIa	1.00	0.00	n/a	n/a	0.00	1.00	41.84	2.68
548	10220	Spain	Catalonia: Girona: La Selva: Riudarenes	50	IIa	n/a	n/a	n/a	n/a	n/a	n/a	41.84	2.68
549	10221	Spain	Catalonia: Girona: La Selva: Riudarenes	50	IIa	1.00	0.00	n/a	n/a	0.00	1.00	41.84	2.68
550	10222	Spain	Catalonia: Girona: La Selva: Riudarenes	50	IIa	1.00	0.00	n/a	n/a	0.00	1.00	41.84	2.68
551	10223	Spain	Catalonia: Girona: La Selva: Riudarenes	50	IIa	n/a	n/a	n/a	n/a	n/a	n/a	41.84	2.68
552	10225	Spain	Catalonia: Girona: La Selva: Riudarenes	50	IIa	n/a	n/a	n/a	n/a	n/a	n/a	41.84	2.68
553	10226	Spain	Catalonia: Girona: La Selva: Riudarenes	50	IIa	n/a	n/a	n/a	n/a	n/a	n/a	41.84	2.68
554	10227	Spain	Catalonia: Girona: La Selva: Riudarenes	50	IIa	1.00	0.00	n/a	n/a	0.00	1.00	41.84	2.68
555	10228	Spain	Catalonia: Girona: La Selva: Riudarenes	50	IIa	1.00	0.00	n/a	n/a	0.00	1.00	41.84	2.68
556	10229	Spain	Catalonia: Girona: La Selva: Riudarenes	50	IIa	n/a	n/a	n/a	n/a	n/a	n/a	41.84	2.68
557	10230	Spain	Catalonia: Girona: La Selva: Riudarenes	50	IIa	1.00	0.00	n/a	n/a	0.00	1.00	41.84	2.68
558	10231	Spain	Catalonia: Girona: La Selva: Riudarenes	50	IIa	n/a	n/a	n/a	n/a	n/a	n/a	41.84	2.68
559	10232	Spain	Catalonia: Girona: La Selva: Riudarenes	50	IIa	n/a	n/a	n/a	n/a	n/a	n/a	41.84	2.68
560	10233	Spain	Catalonia: Girona: La Selva: Riudarenes	50	IIa	n/a	n/a	n/a	n/a	n/a	n/a	41.84	2.68
561	10234	Spain	Catalonia: Girona: La Selva: Riudarenes	50	IIa	n/a	n/a	n/a	n/a	n/a	n/a	41.84	2.68
562	10235	Spain	Catalonia: Girona: La Selva: Riudarenes	50	IIa	1.00	0.00	n/a	n/a	0.00	1.00	41.84	2.68
563	10236	Spain	Catalonia: Girona: La Selva: Riudarenes	50	IIa	n/a	n/a	n/a	n/a	n/a	n/a	41.84	2.68
564	10237	Spain	Catalonia: Girona: La Selva: Riudarenes	50	IIa	n/a	n/a	n/a	n/a	n/a	n/a	41.84	2.68
565	10238	Spain	Catalonia: Girona: La Selva: Riudarenes	50	IIa	n/a	n/a	n/a	n/a	n/a	n/a	41.84	2.68
566	10239	Spain	Catalonia: Girona: La Selva: Riudarenes	50	IIa	1.00	0.00	n/a	n/a	0.00	1.00	41.84	2.68
567	10240	Spain	Catalonia: Girona: La Selva: Riudarenes	50	IIa	n/a	n/a	n/a	n/a	n/a	n/a	41.84	2.68
568	10241	Spain	Catalonia: Girona: La Selva: Riudarenes	50	IIa	n/a	n/a	n/a	n/a	n/a	n/a	41.84	2.68
569	10242	Spain	Catalonia: Girona: La Selva: Riudarenes	50	IIa	1.00	0.00	n/a	n/a	0.00	1.00	41.84	2.68
570	10243	Spain	Catalonia: Girona: La Selva: Riudarenes	50	IIa	n/a	n/a	n/a	n/a	n/a	n/a	41.84	2.68
571	10244	Spain	Catalonia: Girona: La Selva: Riudarenes	50	IIa	n/a	n/a	n/a	n/a	n/a	n/a	41.84	2.68
572	10245	Spain	Catalonia: Girona: La Selva: Riudarenes	50	IIa	n/a	n/a	n/a	n/a	n/a	n/a	41.84	2.68
573	10246	Spain	Catalonia: Girona: La Selva: Riudarenes	50	IIa	n/a	n/a	n/a	n/a	n/a	n/a	41.84	2.68
574	10247	Spain	Catalonia: Girona: La Selva: Riudarenes	50	n/a	1.00	0.00	n/a	n/a	0.00	1.00	41.84	2.68
575	10248	Spain	Catalonia: Girona: La Selva: Riudarenes	50	IIa	n/a	n/a	n/a	n/a	n/a	n/a	41.84	2.68
576	10249	Spain	Catalonia: Girona: La Selva: Riudarenes	50	IIa	1.00	0.00	n/a	n/a	0.00	1.00	41.84	2.68
577	10250	Spain	Catalonia: Girona: La Selva: Riudarenes	50	IIa	1.00	0.00	n/a	n/a	0.00	1.00	41.84	2.68
578	10251	Spain	Catalonia: Girona: La Selva: Riudarenes	50	IIa	n/a	n/a	n/a	n/a	n/a	n/a	41.84	2.68
579	10252	Spain	Catalonia: Girona: La Selva: Riudarenes	50	IIa	n/a	n/a	n/a	n/a	n/a	n/a	41.84	2.68
580	10253	Spain	Catalonia: Girona: La Selva: Riudarenes	50	IIa	n/a	n/a	n/a	n/a	n/a	n/a	41.84	2.68
581	10254	Spain	Catalonia: Girona: La Selva: Riudarenes	50	IIa	n/a	n/a	n/a	n/a	n/a	n/a	41.84	2.68
582	10255	Spain	Catalonia: Girona: La Selva: Riudarenes	50	IIa	n/a	n/a	n/a	n/a	n/a	n/a	41.84	2.68
583	10256	Spain	Catalonia: Girona: La Selva: Riudarenes	50	IIa	n/a	n/a	n/a	n/a	n/a	n/a	41.84	2.68
584	10257	Spain	Catalonia: Girona: La Selva: Riudarenes	50	Va	n/a	n/a	n/a	n/a	n/a	n/a	41.84	2.68
585	10258	Spain	Catalonia: Girona: La Selva: Riudarenes	50	IIa	n/a	n/a	n/a	n/a	n/a	n/a	41.84	2.68

#	MTD-T	Country	Locality	Sampling Site	Haplotype Cyt *b*	*K* =2		*K* =2 (subset north)		*K* =2 (subset south)		N	E
■ Table 1 continued						Red	Blue	Grey	Turqouis	Yellow	Purple		
586	10259	Spain	Catalonia: Girona: La Selva: Riudarenes	50	IIa	n/a	n/a	n/a	n/a	n/a	n/a	41.84	2.68
587	10260	Spain	Catalonia: Girona: La Selva: Riudarenes	50	IIa	n/a	n/a	n/a	n/a	n/a	n/a	41.84	2.68
588	10261	Spain	Catalonia: Girona: La Selva: Riudarenes	50	IIa	n/a	n/a	n/a	n/a	n/a	n/a	41.84	2.68
589	10262	Spain	Catalonia: Girona: La Selva: Riudarenes	50	IIa	n/a	n/a	n/a	n/a	n/a	n/a	41.84	2.68
590	10263	Spain	Catalonia: Girona: La Selva: Riudarenes	50	IIa	n/a	n/a	n/a	n/a	n/a	n/a	41.84	2.68
591	10264	Spain	Catalonia: Girona: La Selva: Riudarenes	50	IIa	n/a	n/a	n/a	n/a	n/a	n/a	41.84	2.68
592	10265	Spain	Catalonia: Girona: La Selva: Riudarenes	50	IIa	n/a	n/a	n/a	n/a	n/a	n/a	41.84	2.68
593	10266	Spain	Catalonia: Girona: La Selva: Riudarenes	50	IIa	n/a	n/a	n/a	n/a	n/a	n/a	41.84	2.68
594	10267	Spain	Catalonia: Girona: La Selva: Riudarenes	50	IIa	n/a	n/a	n/a	n/a	n/a	n/a	41.84	2.68
595	10269	Spain	Catalonia: Girona: La Selva: Riudarenes	50	n/a	1.00	0.00	n/a	n/a	0.00	1.00	41.84	2.68
596	10271	Spain	Catalonia: Girona: La Selva: Riudarenes	50	n/a	1.00	0.00	n/a	n/a	0.00	1.00	41.84	2.68
597	10273	Spain	Catalonia: Girona: La Selva: Riudarenes	50	n/a	1.00	0.00	n/a	n/a	0.00	1.00	41.84	2.68
598	10277	Spain	Catalonia: Girona: La Selva: Riudarenes	50	n/a	1.00	0.00	n/a	n/a	0.00	1.00	41.84	2.68
599	10218	Spain	Catalonia: Girona: La Selva: Riudarenes	50	IIa	n/a	n/a	n/a	n/a	n/a	n/a	41.84	2.68
600	10285	Spain	Catalonia: Girona: La Selva: Riudarenes	50	IIa	n/a	n/a	n/a	n/a	n/a	n/a	41.84	2.68
601	10286	Spain	Catalonia: Girona: La Selva: Riudarenes	50	IIa	n/a	n/a	n/a	n/a	n/a	n/a	41.84	2.68
602	10287	Spain	Catalonia: Girona: La Selva: Riudarenes	50	IIa	n/a	n/a	n/a	n/a	n/a	n/a	41.84	2.68
603	10288	Spain	Catalonia: Girona: La Selva: Riudarenes	50	IIa	n/a	n/a	n/a	n/a	n/a	n/a	41.84	2.68
604	10289	Spain	Catalonia: Girona: La Selva: Riudarenes	50	IIa	n/a	n/a	n/a	n/a	n/a	n/a	41.84	2.68
605	10290	Spain	Catalonia: Girona: La Selva: Riudarenes	50	IIa	n/a	n/a	n/a	n/a	n/a	n/a	41.84	2.68
606	10291	Spain	Catalonia: Girona: La Selva: Riudarenes	50	IIa	n/a	n/a	n/a	n/a	n/a	n/a	41.84	2.68
607	10292	Spain	Catalonia: Girona: La Selva: Riudarenes	50	IIa	1.00	0.00	n/a	n/a	0.00	1.00	41.84	2.68
608	10293	Spain	Catalonia: Girona: La Selva: Riudarenes	50	IIa	1.00	0.00	n/a	n/a	0.00	1.00	41.84	2.68
609	10295	Spain	Catalonia: Girona: La Selva: Riudarenes	50	IIa	n/a	n/a	n/a	n/a	n/a	n/a	41.84	2.68
610	10296	Spain	Catalonia: Girona: La Selva: Riudarenes	50	IIa	1.00	0.00	n/a	n/a	0.00	1.00	41.84	2.68
611	10313	Spain	Catalonia: Girona: La Selva: Riudarenes	50	IIa	1.00	0.00	n/a	n/a	0.00	1.00	41.84	2.68
612	1581	Spain	Girona: Ter River	51	VIa	0.01	0.99	n/a	n/a	1.00	0.01	41.97	2.72
613	10312	Spain	Catalonia: Girona: La Selva: Vilobí d'Onyar	52	IIa	1.00	0.01	n/a	n/a	0.01	0.99	41.89	2.74
614	5380	Spain	Catalonia: Girona: La Selva: Vilobí d'Onyar	52	IIa	1.00	0.00	n/a	n/a	0.01	1.00	41.89	2.74
615	8485	Spain	Catalonia: Girona: Pla de l'Estany: Banyoles (Lake)	53	VIa	0.00	1.00	n/a	n/a	1.00	0.00	42.13	2.75
616	8487	Spain	Catalonia: Girona: Pla de l'Estany: Banyoles (Lake)	53	IIa	0.99	0.01	n/a	n/a	0.01	0.99	42.13	2.75
617	7497	Spain	Catalonia: Girona: La Selva: Sils	54	IIa	1.00	0.00	n/a	n/a	0.00	1.00	41.80	2.74
618	7498	Spain	Catalonia: Girona: La Selva: Sils	54	IIa	1.00	0.00	n/a	n/a	0.00	1.00	41.80	2.74
619	7499	Spain	Catalonia: Girona: La Selva: Sils	54	IIa	1.00	0.00	n/a	n/a	0.00	1.00	41.80	2.74
620	7500	Spain	Catalonia: Girona: La Selva: Sils	54	IIa	1.00	0.00	n/a	n/a	0.00	1.00	41.80	2.74
621	7501	Spain	Catalonia: Girona: La Selva: Sils	54	IIa	1.00	0.01	n/a	n/a	0.00	1.00	41.80	2.74
622	7502	Spain	Catalonia: Girona: La Selva: Sils	54	IIa	1.00	0.00	n/a	n/a	0.00	1.00	41.80	2.74
623	7503	Spain	Catalonia: Girona: La Selva: Sils	54	IIa	1.00	0.00	n/a	n/a	0.00	1.00	41.80	2.74
624	7504	Spain	Catalonia: Girona: La Selva: Sils	54	IIa	1.00	0.00	n/a	n/a	0.00	1.00	41.80	2.74
625	8002	Spain	Catalonia: Girona: La Selva: Sils	54	IIa	1.00	0.00	n/a	n/a	0.00	1.00	41.80	2.74
626	8003	Spain	Catalonia: Girona: La Selva: Sils	54	IIa	1.00	0.00	n/a	n/a	0.00	1.00	41.80	2.74

#	MTD-T	Country	Locality	Sampling Site	Haplotype Cyt *b*	*K* =2		*K* =2 (subset north)		*K* =2 (subset south)		N	E
■ **Table 1 continued**						**Red**	**Blue**	**Grey**	**Turqouis**	**Yellow**	**Purple**		
627	8004	Spain	Catalonia: Girona: La Selva: Sils	54	IIa	1.00	0.00	n/a	n/a	0.00	1.00	41.80	2.74
628	8005	Spain	Catalonia: Girona: La Selva: Sils	54	IIa	1.00	0.00	n/a	n/a	0.00	1.00	41.80	2.74
629	8006	Spain	Catalonia: Girona: La Selva: Sils	54	IIa	1.00	0.00	n/a	n/a	0.00	1.00	41.80	2.74
630	8007	Spain	Catalonia: Girona: La Selva: Sils	54	IIa	1.00	0.00	n/a	n/a	0.00	1.00	41.80	2.74
631	8008	Spain	Catalonia: Girona: La Selva: Sils	54	IIa	1.00	0.00	n/a	n/a	0.00	1.00	41.80	2.74
632	8009	Spain	Catalonia: Girona: La Selva: Sils	54	IIa	1.00	0.00	n/a	n/a	0.02	0.98	41.80	2.74
633	8010	Spain	Catalonia: Girona: La Selva: Sils	54	IIa	1.00	0.00	n/a	n/a	0.01	1.00	41.80	2.74
634	8011	Spain	Catalonia: Girona: La Selva: Sils	54	IIa	1.00	0.00	n/a	n/a	0.00	1.00	41.80	2.74
635	8012	Spain	Catalonia: Girona: La Selva: Sils	54	IIa	1.00	0.00	n/a	n/a	0.00	1.00	41.80	2.74
636	8013	Spain	Catalonia: Girona: La Selva: Sils	54	IIa	1.00	0.00	n/a	n/a	0.00	1.00	41.80	2.74
637	8014	Spain	Catalonia: Girona: La Selva: Sils	54	IIa	1.00	0.00	n/a	n/a	0.01	0.99	41.80	2.74
638	8015	Spain	Catalonia: Girona: La Selva: Sils	54	IIa	1.00	0.01	n/a	n/a	0.00	1.00	41.80	2.74
639	10170	Spain	Catalonia: Girona: La Selva: Caldes de Malavella: Riera de Santa Maria (Depuradora)	55	IIa	1.00	0.00	n/a	n/a	0.00	1.00	41.83	2.79
640	10171	Spain	Catalonia: Girona: La Selva: Caldes de Malavella: Riera de Santa Maria (Depuradora)	55	IIa	1.00	0.00	n/a	n/a	0.00	1.00	41.83	2.79
641	10172	Spain	Catalonia: Girona: La Selva: Caldes de Malavella: Riera de Santa Maria (Depuradora)	55	IIa	1.00	0.00	n/a	n/a	0.00	1.00	41.83	2.79
642	10174	Spain	Catalonia: Girona: La Selva: Caldes de Malavella: Riera de Santa Maria (Depuradora)	55	IIa	1.00	0.00	n/a	n/a	0.00	1.00	41.83	2.79
643	10175	Spain	Catalonia: Girona: La Selva: Caldes de Malavella: Riera de Santa Maria (Depuradora)	55	Ib	1.00	0.00	n/a	n/a	0.00	1.00	41.83	2.79
644	10176	Spain	Catalonia: Girona: La Selva: Caldes de Malavella: Riera de Santa Maria (Depuradora)	55	IIa	n/a	n/a	n/a	n/a	n/a	n/a	41.83	2.79
645	10177	Spain	Catalonia: Girona: La Selva: Caldes de Malavella: Riera de Santa Maria (Depuradora)	55	IIa	1.00	0.00	n/a	n/a	0.00	1.00	41.83	2.79
646	10178	Spain	Catalonia: Girona: La Selva: Caldes de Malavella: Riera de Santa Maria (Depuradora)	55	IIa	n/a	n/a	n/a	n/a	n/a	n/a	41.83	2.79
647	10179	Spain	Catalonia: Girona: La Selva: Caldes de Malavella: Riera de Santa Maria (Depuradora)	55	n/a	1.00	0.00	n/a	n/a	0.01	0.99	41.83	2.79
648	10180	Spain	Catalonia: Girona: La Selva: Caldes de Malavella: Riera de Santa Maria (Depuradora)	55	IIa	n/a	n/a	n/a	n/a	n/a	n/a	41.83	2.79
649	10181	Spain	Catalonia: Girona: La Selva: Caldes de Malavella: Riera de Santa Maria (Depuradora)	55	Va	n/a	n/a	n/a	n/a	n/a	n/a	41.83	2.79
650	10182	Spain	Catalonia: Girona: La Selva: Caldes de Malavella: Riera de Santa Maria (Depuradora)	55	n/a	0.97	0.03	n/a	n/a	0.03	0.97	41.83	2.79
651	10183	Spain	Catalonia: Girona: La Selva: Caldes de Malavella: Riera de Santa Maria (Depuradora)	55	IIa	n/a	n/a	n/a	n/a	n/a	n/a	41.83	2.79
652	10184	Spain	Catalonia: Girona: La Selva: Caldes de Malavella: Riera de Santa Maria (Depuradora)	55	IIa	n/a	n/a	n/a	n/a	n/a	n/a	41.83	2.79

#	MTD-T	Country	Locality	Sampling Site	Haplotype Cyt *b*	*K* =2		*K* =2 (subset north)		*K* =2 (subset south)		N	E
■ Table 1 continued						Red	Blue	Grey	Turqouis	Yellow	Purple		
653	10185	Spain	Catalonia: Girona: La Selva: Caldes de Malavella: Riera de Santa Maria (Depuradora)	55	IIa	1.00	0.00	n/a	n/a	0.00	1.00	41.83	2.79
654	10186	Spain	Catalonia: Girona: La Selva: Caldes de Malavella: Riera de Santa Maria (Depuradora)	55	IIa	1.00	0.00	n/a	n/a	0.00	1.00	41.83	2.79
655	10187	Spain	Catalonia: Girona: La Selva: Caldes de Malavella: Riera de Santa Maria (Depuradora)	55	IIa	n/a	n/a	n/a	n/a	n/a	n/a	41.83	2.79
656	10188	Spain	Catalonia: Girona: La Selva: Caldes de Malavella: Riera de Santa Maria (Depuradora)	55	Va	1.00	0.00	n/a	n/a	0.00	1.00	41.83	2.79
657	10189	Spain	Catalonia: Girona: La Selva: Caldes de Malavella: Riera de Santa Maria (Depuradora)	55	IIa	n/a	n/a	n/a	n/a	n/a	n/a	41.83	2.79
658	10190	Spain	Catalonia: Girona: La Selva: Caldes de Malavella: Riera de Santa Maria (Depuradora)	55	IIa	1.00	0.00	n/a	n/a	0.00	1.00	41.83	2.79
659	10192	Spain	Catalonia: Girona: La Selva: Caldes de Malavella: Riera de Santa Maria (Depuradora)	55	IIa	n/a	n/a	n/a	n/a	n/a	n/a	41.83	2.79
660	10193	Spain	Catalonia: Girona: La Selva: Caldes de Malavella: Riera de Santa Maria (Depuradora)	55	IIa	n/a	n/a	n/a	n/a	n/a	n/a	41.83	2.79
661	10194	Spain	Catalonia: Girona: La Selva: Caldes de Malavella: Riera de Santa Maria (Depuradora)	55	IIa	1.00	0.00	n/a	n/a	0.00	1.00	41.83	2.79
662	10195	Spain	Catalonia: Girona: La Selva: Caldes de Malavella: Riera de Santa Maria (Depuradora)	55	IIa	n/a	n/a	n/a	n/a	n/a	n/a	41.83	2.79
663	10196	Spain	Catalonia: Girona: La Selva: Caldes de Malavella: Riera de Santa Maria (Depuradora)	55	n/a	0.99	0.01	n/a	n/a	0.21	0.79	41.83	2.79
664	10198	Spain	Catalonia: Girona: La Selva: Caldes de Malavella: Riera de Santa Maria (Depuradora)	55	n/a	1.00	0.00	n/a	n/a	0.00	1.00	41.83	2.79
665	10201	Spain	Catalonia: Girona: La Selva: Caldes de Malavella: Riera de Santa Maria (Depuradora)	55	n/a	0.99	0.01	n/a	n/a	0.04	0.96	41.83	2.79
666	10206	Spain	Catalonia: Girona: La Selva: Caldes de Malavella: Riera de Santa Maria (Depuradora)	55	n/a	1.00	0.00	n/a	n/a	0.00	1.00	41.83	2.79
667	10209	Spain	Catalonia: Girona: La Selva: Caldes de Malavella: Riera de Santa Maria (Depuradora)	55	n/a	1.00	0.00	n/a	n/a	0.00	1.00	41.83	2.79
668	10215	Spain	Catalonia: Girona: La Selva: Caldes de Malavella: Riera de Santa Maria (Depuradora)	55	n/a	1.00	0.01	n/a	n/a	0.01	0.99	41.83	2.79
669	10217	Spain	Catalonia: Girona: La Selva: Caldes de Malavella: Riera de Santa Maria (Depuradora)	55	n/a	1.00	0.00	n/a	n/a	0.00	1.00	41.83	2.79
670	8486	Spain	Catalonia: Girona: Alt Empordà: Aiguamolls Natural Park	56	Va	0.19	0.81	n/a	n/a	0.76	0.24	42.28	3.12
671	8488	Spain	Catalonia: Girona: Alt Empordà: Aiguamolls Natural Park	56	Va	0.26	0.75	n/a	n/a	0.70	0.30	42.28	3.12
672	5691	Spain	Catalonia: Girona: Alt Empordà: Aiguamolls Natural Park	56	VIa	0.01	1.00	n/a	n/a	1.00	0.01	42.28	3.12
673	5692	Spain	Catalonia: Girona: Alt Empordà: Aiguamolls Natural Park	56	VIb	0.05	0.95	n/a	n/a	0.93	0.07	42.28	3.12
674	11051	Spain	Catalonia: Girona: Alt Empordà: Aiguamolls Natural Park	56	Va	0.02	0.98	n/a	n/a	0.93	0.07	42.28	3.12
675	1803	Spain	Catalonia: Girona: Alt Empordà: Aiguamolls Natural Park	56	Va	0.93	0.07	n/a	n/a	0.24	0.76	42.28	3.12
676	1804	Spain	Catalonia: Girona: Alt Empordà: Aiguamolls Natural Park	56	Va	0.99	0.01	n/a	n/a	0.29	0.71	42.28	3.12

#	MTD-T	Country	Locality	Sampling Site	Haplotype Cyt *b*	*K* =2		*K* =2 (subset north)		*K* =2 (subset south)		N	E
■ Table 1 continued						Red	Blue	Grey	Turqouis	Yellow	Purple		
677	1805	Spain	Catalonia: Girona: Alt Empordà: Aiguamolls Natural Park	56	Va	0.63	0.37	n/a	n/a	0.67	0.33	42.28	3.12
678	10159	Spain	Catalonia: Girona: Baix Ter	57	n/a	0.99	0.01	n/a	n/a	0.01	0.99	42.00	3.18
679	10160	Spain	Catalonia: Girona: Baix Ter	57	IIa	1.00	0.00	n/a	n/a	0.00	1.00	42.00	3.18
680	10161	Spain	Catalonia: Girona: Baix Ter	57	n/a	1.00	0.01	n/a	n/a	0.00	1.00	42.00	3.18
681	10162	Spain	Catalonia: Girona: Baix Ter	57	n/a	0.99	0.01	n/a	n/a	0.01	0.99	42.00	3.18
682	10163	Spain	Catalonia: Girona: Baix Ter	57	n/a	1.00	0.01	n/a	n/a	0.00	1.00	42.00	3.18
683	10164	Spain	Catalonia: Girona: Baix Ter	57	IIa	1.00	0.00	n/a	n/a	0.01	0.99	42.00	3.18
684	10165	Spain	Catalonia: Girona: Baix Ter	57	n/a	1.00	0.00	n/a	n/a	0.00	1.00	42.00	3.18
685	10166	Spain	Catalonia: Girona: Baix Ter	57	n/a	0.99	0.01	n/a	n/a	0.02	0.99	42.00	3.18
686	10167	Spain	Catalonia: Girona: Baix Ter	57	n/a	0.63	0.37	n/a	n/a	0.52	0.48	42.00	3.18
687	10168	Spain	Catalonia: Girona: Baix Ter	57	n/a	1.00	0.01	n/a	n/a	0.00	1.00	42.00	3.18
688	10169	Spain	Catalonia: Girona: Baix Ter	57	IIa	0.99	0.01	n/a	n/a	0.01	0.99	42.00	3.18
689	8045	Spain	Catalonia: Girona: Baix Ter	57	n/a	0.61	0.40	n/a	n/a	0.57	0.43	42.00	3.18
690	8046	Spain	Catalonia: Girona: Baix Ter	57	IIa	0.99	0.01	n/a	n/a	0.01	0.99	42.00	3.18
691	8048	Spain	Catalonia: Girona: Baix Ter	57	IIa	1.00	0.00	n/a	n/a	0.00	1.00	42.00	3.18
692	8049	Spain	Catalonia: Girona: Baix Ter	57	IIa	1.00	0.01	n/a	n/a	0.00	1.00	42.00	3.18
693	8050	Spain	Catalonia: Girona: Baix Ter	57	IIa	1.00	0.00	n/a	n/a	0.00	1.00	42.00	3.18
694	8051	Spain	Catalonia: Girona: Baix Ter	57	IIa	0.99	0.01	n/a	n/a	0.01	0.99	42.00	3.18
695	2602	France	Pyrenees-Atlantiques: Bassussari: Xurrumilatx	58	IIa	0.99	0.01	0.998	0.002	n/a	n/a	43.43	-1.50
696	1610	France	Pyrenees-Atlantiques: Bassussari: Xurrumilatx	58	IIa	0.98	0.02	0.996	0.004	n/a	n/a	43.43	-1.50
697	1611	France	Pyrenees-Atlantiques: Bassussari: Xurrumilatx	58	IIa	0.99	0.01	0.997	0.003	n/a	n/a	43.43	-1.50
698	1612	France	Pyrenees-Atlantiques: Bassussari: Xurrumilatx	58	IIa	0.98	0.02	0.996	0.004	n/a	n/a	43.43	-1.50
699	2615	France	Landes: Labenne: Marais d'Orx	59	IIa	1.00	0.00	0.99	0.01	n/a	n/a	43.60	-1.40
700	2616	France	Landes: Labenne: Marais d'Orx	59	IIa	1.00	0.01	0.991	0.009	n/a	n/a	43.60	-1.40
701	2617	France	Landes: Labenne: Marais d'Orx	59	IIa	0.95	0.05	0.976	0.024	n/a	n/a	43.60	-1.40
702	2618	France	Landes: Labenne: Marais d'Orx	59	IIa	0.80	0.21	0.93	0.07	n/a	n/a	43.60	-1.40
703	2619	France	Landes: Labenne: Marais d'Orx	59	IIa	0.99	0.01	0.996	0.004	n/a	n/a	43.60	-1.40
704	2620	France	Landes: Labenne: Marais d'Orx	59	IIa	1.00	0.00	0.998	0.002	n/a	n/a	43.60	-1.40
705	2621	France	Landes: Labenne: Marais d'Orx	59	IIa	0.99	0.01	0.992	0.008	n/a	n/a	43.60	-1.40
706	2622	France	Landes: Labenne: Marais d'Orx	59	IIa	0.41	0.60	0.997	0.003	n/a	n/a	43.60	-1.40
707	2623	France	Landes: Seignosse: Étang Noir	60	IIa	1.00	0.00	0.997	0.003	n/a	n/a	43.70	-1.36
708	2624	France	Landes: Seignosse: Étang Noir	60	IIa	1.00	0.01	0.991	0.009	n/a	n/a	43.70	-1.36
709	2625	France	Landes: Seignosse: Étang Noir	60	IIa	1.00	0.00	0.994	0.006	n/a	n/a	43.70	-1.36
710	2597	France	Pyrenees-Atlantiques: Urt: Barthes de Munho	61	IIa	0.99	0.01	0.986	0.014	n/a	n/a	43.50	-1.29
711	2632	France	Gironde: Arès: Domaine de Saint Brice	62	IIa	1.00	0.00	0.996	0.004	n/a	n/a	44.78	-1.15
712	2633	France	Gironde: Arès: Domaine de Saint Brice	62	IIa	0.99	0.01	0.992	0.008	n/a	n/a	44.78	-1.15
713	2634	France	Gironde: Arès: Domaine de Saint Brice	62	IIa	0.99	0.01	0.993	0.007	n/a	n/a	44.78	-1.15
714	2635	France	Gironde: Arès: Domaine de Saint Brice	62	IIa	0.99	0.01	0.994	0.006	n/a	n/a	44.78	-1.15
715	2636	France	Gironde: Arès: Domaine de Saint Brice	62	IIa	0.99	0.01	0.995	0.005	n/a	n/a	44.78	-1.15
716	2638	France	Gironde: Le Verdon-sur-Mer: Marais du Logis	63	IIa	0.87	0.13	0.994	0.006	n/a	n/a	45.54	-1.08
717	2639	France	Gironde: Le Verdon-sur-Mer: Marais du Logis	63	IIa	0.99	0.01	0.994	0.006	n/a	n/a	45.54	-1.08

#	MTD-T	Country	Locality	Sampling Site	Haplotype Cyt *b*	*K* =2		*K* =2 (subset north)		*K* =2 (subset south)		N	E
■ Table 1 continued						Red	Blue	Grey	Turquois	Yellow	Purple		
718	1511	France	Gironde: Hourtin	64	Va	0.99	0.01	0.996	0.004	n/a	n/a	45.18	-1.06
719	2626	France	Landes: Dax: Bois de Boulogne	65	IIa	1.00	0.00	0.995	0.005	n/a	n/a	43.71	-1.08
720	2627	France	Landes: Dax: Bois de Boulogne	65	IIa	1.00	0.01	0.981	0.019	n/a	n/a	43.71	-1.08
721	2628	France	Landes: Dax: Bois de Boulogne	65	IIa	0.99	0.01	0.997	0.003	n/a	n/a	43.71	-1.08
722	2629	France	Landes: Dax: Barthes de Dax	66	IIa	0.99	0.01	0.997	0.003	n/a	n/a	43.70	-1.08
723	2630	France	Landes: Dax: Barthes de Dax	66	IIa	0.99	0.01	0.997	0.003	n/a	n/a	43.70	-1.08
724	2631	France	Landes: Dax: Barthes de Dax	66	IIa	0.99	0.01	0.994	0.006	n/a	n/a	43.70	-1.08
725	2610	France	Gironde: Bruges: Marais de Bruges	67	IIa	1.00	0.00	0.995	0.005	n/a	n/a	44.90	-0.60
726	2611	France	Gironde: Bruges: Marais de Bruges	67	IIa	0.99	0.01	0.996	0.004	n/a	n/a	44.90	-0.60
727	2612	France	Gironde: Bruges: Marais de Bruges	67	IIa	0.99	0.01	0.991	0.009	n/a	n/a	44.90	-0.60
728	2613	France	Gironde: Bruges: Marais de Bruges	67	IIa	1.00	0.00	0.964	0.036	n/a	n/a	44.90	-0.60
729	2614	France	Gironde: Bruges: Marais de Bruges	67	IIa	1.00	0.00	0.99	0.01	n/a	n/a	44.90	-0.60
730	1622	France	Pyrénées-Atlantiques: Vicinity of Doazon	68	IIa	1.00	0.00	0.992	0.008	n/a	n/a	43.44	-0.53
731	1623	France	Pyrénées-Atlantiques: Vicinity of Doazon	68	IIh	1.00	0.00	0.996	0.004	n/a	n/a	43.44	-0.53
732	1624	France	Pyrénées-Atlantiques: Vicinity of Doazon	68	IIh	1.00	0.00	0.996	0.004	n/a	n/a	43.44	-0.53
733	1625	France	Pyrénées-Atlantiques: Vicinity of Doazon	68	IIa	0.88	0.12	0.987	0.013	n/a	n/a	43.44	-0.53
734	1626	France	Pyrénées-Atlantiques: Vicinity of Doazon	68	IIa	1.00	0.00	0.993	0.007	n/a	n/a	43.44	-0.53
735	1513	France	Dordogne: La Jemaye: Étang de La Jemaye	69	IIa	0.99	0.01	0.962	0.038	n/a	n/a	45.16	0.28
736	1514	France	Dordogne: La Jemaye: Étang de La Jemaye	69	IIa	0.99	0.01	0.994	0.006	n/a	n/a	45.16	0.28
737	1515	France	Dordogne: La Jemaye: Étang de La Jemaye	69	IIa	0.99	0.01	0.988	0.012	n/a	n/a	45.16	0.28
738	1516	France	Dordogne: La Jemaye: Étang de La Jemaye	69	IIa	0.99	0.01	0.991	0.009	n/a	n/a	45.16	0.28
739	1517	France	Dordogne: La Jemaye: Étang de La Jemaye	69	IIa	1.00	0.00	0.994	0.006	n/a	n/a	45.16	0.28
740	1518	France	Dordogne: La Jemaye: Étang de La Jemaye	69	IIa	0.99	0.01	0.994	0.006	n/a	n/a	45.16	0.28
741	1519	France	Dordogne: La Jemaye: Étang de La Jemaye	69	IIa	1.00	0.00	0.994	0.006	n/a	n/a	45.16	0.28
742	1520	France	Dordogne: La Jemaye: Étang de La Jemaye	69	IIa	0.99	0.01	0.997	0.003	n/a	n/a	45.16	0.28
743	1521	France	Dordogne: La Jemaye: Étang de La Jemaye	69	IIa	1.00	0.01	0.992	0.008	n/a	n/a	45.16	0.28
744	1601	France	Dordogne: La Jemaye: Étang de La Jemaye	69	IIa	1.00	0.00	0.968	0.032	n/a	n/a	45.16	0.28
745	1602	France	Dordogne: La Jemaye: Étang de La Jemaye	69	IIa	1.00	0.01	0.978	0.022	n/a	n/a	45.16	0.28
746	1603	France	Dordogne: La Jemaye: Étang de La Jemaye	69	IIa	1.00	0.00	0.993	0.007	n/a	n/a	45.16	0.28
747	1604	France	Dordogne: La Jemaye: Étang de La Jemaye	69	IIa	1.00	0.00	0.99	0.01	n/a	n/a	45.16	0.28
748	1605	France	Dordogne: La Jemaye: Étang de La Jemaye	69	IIa	0.99	0.01	0.996	0.004	n/a	n/a	45.16	0.28
749	1606	France	Dordogne: La Jemaye: Étang de La Jemaye	69	IIa	1.00	0.00	0.993	0.007	n/a	n/a	45.16	0.28
750	1607	France	Dordogne: La Jemaye: Étang de La Jemaye	69	IIa	1.00	0.00	0.993	0.007	n/a	n/a	45.16	0.28
751	7683	France	Indre: Brenne	70	IIa	0.99	0.01	0.998	0.002	n/a	n/a	46.74	1.23
752	7684	France	Indre: Brenne	70	IIa	0.98	0.02	0.997	0.003	n/a	n/a	46.74	1.23
753	7685	France	Indre: Brenne	70	IIa	0.99	0.01	0.997	0.003	n/a	n/a	46.74	1.23
754	11162	France	Indre: Brenne	70	n/a	0.99	0.01	0.998	0.002	n/a	n/a	46.74	1.23
755	11163	France	Indre: Brenne	70	n/a	0.99	0.01	0.997	0.003	n/a	n/a	46.74	1.23
756	7686	France	Indre: Brenne	70	IIa	1.00	0.01	0.997	0.003	n/a	n/a	46.74	1.23
757	7687	France	Indre: Brenne	70	IIa	1.00	0.01	0.997	0.003	n/a	n/a	46.74	1.23
758	7688	France	Indre: Brenne	70	IIa	0.99	0.01	0.997	0.003	n/a	n/a	46.74	1.23

#	MTD-T	Country	Locality	Sampling Site	Haplotype Cyt *b*	*K* =2		*K* =2 (subset north)		*K* =2 (subset south)		N	E
■ **Table 1 continued**						**Red**	**Blue**	**Grey**	**Turquois**	**Yellow**	**Purple**		
759	7689	France	Indre: Brenne	70	IIa	0.99	0.01	0.997	0.003	n/a	n/a	46.74	1.23
760	2502	France	Aude: Leucate	71	Va	1.00	0.00	0.004	0.996	n/a	n/a	42.91	3.04
761	2496	France	Aude: Leucate	71	Va	1.00	0.00	0.024	0.976	n/a	n/a	42.91	3.04
762	2497	France	Aude: Leucate	71	Va	1.00	0.01	0.007	0.993	n/a	n/a	42.91	3.04
763	2498	France	Aude: Leucate	71	Va	1.00	0.00	0.005	0.995	n/a	n/a	42.91	3.04
764	2500	France	Aude: Leucate	71	Va	0.90	0.10	0.008	0.992	n/a	n/a	42.91	3.04
765	2501	France	Aude: Leucate	71	IVb	1.00	0.00	0.003	0.997	n/a	n/a	42.91	3.04
766	2342	France	Hérault: Étang d'Or	72	IIa	1.00	0.00	0.987	0.013	n/a	n/a	43.58	4.03
767	2343	France	Hérault: Étang d'Or	72	IIa	1.00	0.00	0.995	0.005	n/a	n/a	43.58	4.03
768	2344	France	Hérault: Étang d'Or	72	Va	0.99	0.01	0.642	0.358	n/a	n/a	43.58	4.03
769	2345	France	Hérault: Étang d'Or	72	IIa	1.00	0.00	0.96	0.04	n/a	n/a	43.58	4.03
770	2346	France	Hérault: Étang d'Or	72	IIa	0.99	0.01	0.992	0.008	n/a	n/a	43.58	4.03
771	2347	France	Hérault: Étang d'Or	72	IIa	1.00	0.00	0.996	0.004	n/a	n/a	43.58	4.03
772	2483	France	Hérault: Étang d'Or	72	IIa	0.99	0.01	0.991	0.009	n/a	n/a	43.58	4.03
773	2484	France	Hérault: Étang d'Or	72	IIa	1.00	0.00	0.995	0.005	n/a	n/a	43.58	4.03
774	2485	France	Hérault: Étang d'Or	72	IIa	1.00	0.00	0.933	0.067	n/a	n/a	43.58	4.03
775	2482	France	Hérault: Étang d'Or	72	Va	0.99	0.01	0.994	0.006	n/a	n/a	43.58	4.03
776	2480	France	Hérault: Étang d'Or	72	Va	0.99	0.01	0.991	0.009	n/a	n/a	43.58	4.03
777	2481	France	Hérault: Étang d'Or	72	Va	0.99	0.01	0.874	0.126	n/a	n/a	43.58	4.03
778	2486	France	Hérault: Candillargues: Le Bérange	73	IIa	1.00	0.00	0.886	0.114	n/a	n/a	43.63	4.08
779	2488	France	Hérault: Candillargues: Le Bérange	73	Va	1.00	0.00	0.985	0.015	n/a	n/a	43.63	4.08
780	2487	France	Hérault: Candillargues: Le Bérange	73	IIa	1.00	0.01	0.981	0.019	n/a	n/a	43.63	4.08
781	2503	France	Gard: Le Cailar: Réserve de Mahistre	74	Va	0.99	0.01	0.882	0.118	n/a	n/a	43.60	4.26
782	2504	France	Gard: Le Cailar: Réserve de Mahistre	74	Va	1.00	0.01	0.987	0.013	n/a	n/a	43.60	4.26
783	2505	France	Gard: Le Cailar: Réserve de Mahistre	74	Va	0.99	0.01	0.993	0.007	n/a	n/a	43.60	4.26
784	2506	France	Gard: Le Cailar: Réserve de Mahistre	74	Va	0.97	0.03	0.972	0.028	n/a	n/a	43.60	4.26
785	335	France	Var: Plan-de-la-Tour	75	Va	1.00	0.00	0.004	0.996	n/a	n/a	43.34	6.55
786	336	France	Var: Plan-de-la-Tour	75	Va	0.99	0.01	0.006	0.994	n/a	n/a	43.34	6.55
787	337	France	Var: Plan-de-la-Tour	75	Va	1.00	0.00	0.003	0.997	n/a	n/a	43.34	6.55
788	338	France	Var: Plan-de-la-Tour	75	Va	0.99	0.01	0.004	0.996	n/a	n/a	43.34	6.55
789	339	France	Var: Plan-de-la-Tour	75	Va	0.99	0.01	0.003	0.997	n/a	n/a	43.34	6.55
790	340	France	Var: Plan-de-la-Tour	75	Va	1.00	0.00	0.003	0.997	n/a	n/a	43.34	6.55
791	341	France	Var: Plan-de-la-Tour	75	Va	1.00	0.00	0.003	0.997	n/a	n/a	43.34	6.55
792	342	France	Var: Plan-de-la-Tour	75	Va	1.00	0.00	0.004	0.996	n/a	n/a	43.34	6.55
793	343	France	Var: Plan-de-la-Tour	75	Va	0.99	0.01	0.003	0.997	n/a	n/a	43.34	6.55
794	344	France	Var: Plan-de-la-Tour	75	Va	1.00	0.00	0.004	0.996	n/a	n/a	43.34	6.55
795	345	France	Var: Plan-de-la-Tour	75	Va	0.97	0.03	0.003	0.997	n/a	n/a	43.34	6.55
796	346	France	Var: Plan-de-la-Tour	75	n/a	0.99	0.01	0.003	0.997	n/a	n/a	43.34	6.55
797	347	France	Var: Plan-de-la-Tour	75	n/a	0.99	0.01	0.004	0.996	n/a	n/a	43.34	6.55
798	348	France	Var: Plan-de-la-Tour	75	Va	0.96	0.04	0.004	0.996	n/a	n/a	43.34	6.55
799	349	France	Var: Plan-de-la-Tour	75	Va	1.00	0.00	0.006	0.994	n/a	n/a	43.34	6.55

#	MTD-T	Country	Locality	Sampling Site	Haplotype Cyt *b*	*K* =2		*K* =2 (subset north)		*K* =2 (subset south)		N	E
■ Table 1 continued						Red	Blue	Grey	Turqouis	Yellow	Purple		
800	350	France	Var: Plan-de-la-Tour	75	n/a	0.97	0.03	0.004	0.996	n/a	n/a	43.34	6.55
801	351	France	Var: Plan-de-la-Tour	75	Va	0.94	0.06	0.012	0.988	n/a	n/a	43.34	6.55
802	352	France	Var: Plan-de-la-Tour	75	Va	0.74	0.26	0.068	0.932	n/a	n/a	43.34	6.55
803	353	France	Var: Plan-de-la-Tour	75	IIa	0.99	0.01	0.006	0.994	n/a	n/a	43.34	6.55
804	354	France	Var: Plan-de-la-Tour	75	Va	1.00	0.01	0.002	0.998	n/a	n/a	43.34	6.55
805	355	France	Var: Plan-de-la-Tour	75	Va	0.99	0.01	0.004	0.996	n/a	n/a	43.34	6.55
806	356	France	Var: Plan-de-la-Tour	75	Va	0.97	0.03	0.005	0.995	n/a	n/a	43.34	6.55
807	7700	France	Var: Ramatuelle	76	Va	1.00	0.00	0.003	0.997	n/a	n/a	43.21	6.61
808	7701	France	Var: Ramatuelle	76	Va	1.00	0.00	0.004	0.996	n/a	n/a	43.21	6.61
809	7703	France	Var: Ramatuelle	76	Va	0.99	0.01	0.009	0.991	n/a	n/a	43.21	6.61
810	7704	France	Var: Ramatuelle	76	Va	0.99	0.01	0.013	0.987	n/a	n/a	43.21	6.61
811	7705	France	Var: Ramatuelle	76	Va	0.98	0.02	0.095	0.905	n/a	n/a	43.21	6.61
812	7707	France	Var: Ramatuelle	76	Va	1.00	0.00	0.01	0.99	n/a	n/a	43.21	6.61
813	7708	France	Var: Ramatuelle	76	Va	0.96	0.04	0.013	0.987	n/a	n/a	43.21	6.61
814	7709	France	Var: Ramatuelle	76	Va	0.99	0.01	0.005	0.995	n/a	n/a	43.21	6.61
815	7710	France	Var: Ramatuelle	76	Va	1.00	0.00	0.004	0.996	n/a	n/a	43.21	6.61
816	7711	France	Var: Ramatuelle	76	Va	1.00	0.00	0.051	0.949	n/a	n/a	43.21	6.61
817	7712	France	Var: Ramatuelle	76	Va	1.00	0.00	0.004	0.996	n/a	n/a	43.21	6.61
818	7713	France	Var: Ramatuelle	76	Va	0.99	0.01	0.027	0.973	n/a	n/a	43.21	6.61
819	7714	France	Var: Ramatuelle	76	Va	0.98	0.02	0.011	0.989	n/a	n/a	43.21	6.61
820	7715	France	Var: Ramatuelle	76	Va	1.00	0.00	0.006	0.994	n/a	n/a	43.21	6.61
821	7716	France	Var: Ramatuelle	76	Va	0.85	0.15	0.328	0.672	n/a	n/a	43.21	6.61
822	7717	France	Var: St. Tropez	77	Va	1.00	0.00	0.994	0.006	n/a	n/a	43.27	6.64
823	7718	France	Var: St. Tropez	77	n/a	1.00	0.01	0.006	0.994	n/a	n/a	43.27	6.64
824	7719	France	Var: St. Tropez	77	Va	0.98	0.02	0.015	0.985	n/a	n/a	43.27	6.64
825	7720	France	Var: St. Tropez	77	Va	0.97	0.03	0.005	0.995	n/a	n/a	43.27	6.64
826	7721	France	Var: St. Tropez	77	Va	0.99	0.01	0.005	0.995	n/a	n/a	43.27	6.64
827	7722	France	Var: St. Tropez	77	Va	1.00	0.00	0.004	0.996	n/a	n/a	43.27	6.64

Table S2. Microsatellite loci, multiplex set, allele size ranges and number of alleles of the individual loci. For primer sequences, see references. Forward primers were fluorescent-labelled.

**Locus**	**Reference**	**Multiplex set**	**Fluorescent label**	**Allele size range (bp)**	**Number of alleles**
msEo21	Pedall *et al.* (2009)	I	ATTO 565	157–197	12
msEo29	Pedall *et al.* (2009)	I	6-FAM	298–336	17
msEo41	Pedall *et al.* (2009)	I	HEX	66–186	26
GmuD107	King & Julian (2004)	I	6-FAM	176–260	14
msEo2	Pedall *et al.* (2009)	II	ATTO 565	104–152	14
GmuD16	King & Julian (2004)	II	HEX	228–312	22
GmuD55	King & Julian (2004)	II	6-FAM	172–298	20
GmuD88	King & Julian (2004)	II	ATTO 565	183–253	16
Emys4	Ciofi *et al.* (2009)	III	ATTO 565	95–149	15
Emys5	Ciofi *et al.* (2009)	III	6-FAM	94–138	22
Emys11	Ciofi *et al.* (2009)	III	HEX	84–132	19
Emys1	Ciofi *et al.* (2009)	IV	6-FAM	112–176	26
Emys6	Ciofi *et al.* (2009)	IV	ATTO 550	129–181	22
Emys7	Ciofi *et al.* (2009)	IV	ATTO 565	106–156	19
Emys8	Ciofi *et al.* (2009)	IV	HEX	97–185	29

Table S3. Akaike information criterion (AIC) scores for fitted clines under different models using the R package HZAR (Derryberry *et al.*, 2013). Bold values with asterisks indicate the best-fit model from the 15-model comparison. *Q* is the proportion of ancestry estimated by STRUCTURE using microsatellite loci. The lowest AIC score indicates the best cline model.

	**Atlantic transect**		**Mediterranean transect**		**Ebro valley**
	**AIC (*Q*)**	**AIC (cyt *b*)**	**AIC (*Q*)**	**AIC (cyt *b*)**	**AIC (*Q*)**
Null model	268.839	223.463	197.467	125.847	546.310
Model 1	26.822	149.697	97.285	59.180	103.084
Model 2	14.426	N/A	95.180	N/A	97.415
Model 3	**14.282***	136.432	58.867	55.302	**62.477***
Model 4	17.803	141.982	62.781	58.920	64.407
Model 5	18.655	N/A	64.882	N/A	64.122
Model 6	22.493	160.070	67.181	63.619	68.431
Model 7	24.251	**122.002***	76.158	**54.725***	76.366
Model 8	15.607	N/A	68.263	N/A	72.018
Model 9	18.400	140.617	63.076	59.272	66.549
Model 10	29.667	153.812	101.333	63.292	106.997
Model 11	16.693	N/A	99.124	N/A	101.473
Model 12	18.404	140.613	61.893	59.466	64.371
Model 13	14.842	147.610	**58.560***	55.143	66.444
Model 14	14.666	N/A	58.582	N/A	66.538
Model 15	18.449	140.614	63.063	59.396	65.300

Table S4. Estimated parameters for the genetic clines using the R package HZAR (Derryberry *et al.*, 2013). *Q* is the proportion of ancestry estimated by STRUCTURE using microsatellite loci. The parameter *c* is the cline center measured in kilometers from the reference site (see Fig. 2, top). The cline width is presented as 1/maximum slope (*w*); d_M is the distance of both introgression tails from the center of the cline, d_L is the distance of the introgression tail to the left from center of the cline, d_R is the distance of the introgression tail to the right from center of the cline, *tauM* is the ratio of the slope of both tails to the slope of the center sigmoid at the transition point, *tauL* is the ratio of the slope of the left tail to the slope of the center sigmoid at the transition point, *tauR* is the ratio of the slope of the right tail to the slope of the center sigmoid at the transition point, *pMin* is the minimum estimated frequency of *Q* at the southern end of the cline, and *pMax* is the maximum estimated frequency at the northern end. CI = confidence interval. N/A stands for not obtained results.

	**Atlantic transect**		**Mediterranean transect**		**Ebro valley**
	Q	**cyt *b***	***Q***	**cyt *b***	***Q***
c	906.14	735.73	745.31	637.24	1099.93
95 % CI	(875.44-949.60)	(730.52-735.73)	(731.70-787.79)	(520.29-675.59)	(1082.71-1200.56)
w	39.18	26.67	40.03	230.52	21.20
95 % CI	(0.01-86.47)	(18.74-26.67)	(1.75-98.60)	(44.96-550.18)	(0.01-109.70)
d_M	N/A	N/A	23.39	N/A	N/A
95 % CI	N/A	N/A	(0.91-56.86)	N/A	N/A
d_L	N/A	N/A	N/A	N/A	N/A
95 % CI	N/A	N/A	N/A	N/A	N/A
d_R	N/A	2.66	N/A	164.18	N/A
95 % CI	N/A	(2.66-4.08)	N/A	(7.99-442.21)	N/A
tauM	N/A	N/A	0.01	N/A	N/A
95 % CI	N/A	N/A	(5.11*e^{-7}- 0.07)	N/A	N/A
tauL	N/A	N/A	N/A	N/A	N/A
95 % CI	N/A	N/A	N/A	N/A	N/A
tauR	N/A	0.09	N/A	0.12	N/A
95 % CI	N/A	(0.03-0.09)	N/A	(9.61*e^{-6}- 0.56)	N/A
pMin	0.03	N/A	N/A	N/A	0.06
95 % CI	(0.003-0.088)	N/A	N/A	N/A	(0.03-0.1)
pMax	0.99	N/A	N/A	N/A	0.98
95 % CI	(0.97-1.00)	N/A	N/A	N/A	(0.96-0.99)

Table S5. Pairwise fixation indices (F_{ST} values) for the comparison of pure (1 and 2) and admixed groups (4 and 5). Above the diagonal, F_{ST} values for the mitochondrial cyt *b* gene; below, F_{ST} values for microsatellite data. All F_{ST} values are significantly different from zero.

	**1 (*occ*)**	**2 (*orb*)**	**4 (N Pyr)**	**5 (SW Pyr)**
**Group 1 (*occidentalis*)**	–	0.975	0.966	0.408
**Group 2 (*orbicularis*)**	0.334	–	0.000	0.650
**Group 4 (N Pyrenees)**	0.326	0.026	–	0.545
**Group 5 (SW Pyrenees)**	0.181	0.160	0.154	-

Table S6. Pairwise fixation indices (F_{ST} values) for the comparison of pure (1–3) and admixed groups (6 and 7). Above the diagonal, F_{ST} values for the mitochondrial cyt *b* gene; below, F_{ST} values for microsatellite data. All F_{ST} values are significantly different from zero.

	**1 (*occ*)**	**2 (*orb*)**	**3 (*gal*)**	**6 (Spa)**	**7 (Fra)**
**Group 1 (*occidentalis*)**	–	0.975	0.950	0.545	0.656
**Group 2 (*orbicularis*)**	0.334	–	1.000	0.520	0.599
**Group 3 (*galloitalica*)**	0.360	0.083	–	0.484	0.430
**Group 6 (Spanish Mediterranean coast)**	0.255	0.081	0.093	–	0.003
**Group 7 (French Mediterranean coast)**	0.338	0.057	0.078	0.082	–

References

Ciofi, C. *et al.* Characterization of microsatellite loci in the European pond turtle *Emys orbicularis*. *Mol. Ecol. Resour.* **9**, 189–191 (2009).

Derryberry, E. P., Derryberry, G. E., Maley, J. M. & Brumfield, R. T. HZAR: hybrid zone analysis using an R software package. *Mol. Ecol. Resour.* **14**, 65–663 (2013).

Fritz, U. *Die Europäische Sumpfschildkröte* (*Emys orbicularis*). Laurenti Verlag (2003).

Fritz, U. *et al.* A new cryptic species of pond turtle from southern Italy, the hottest spot in the range of the genus *Emys*. *Zool. Scr.* **34**, 351–371 (2005).

Fritz, U. *et al.* Mitochondrial phylogeography of European pond turtles (*Emys orbicularis, Emys trinacris*) – an update. *Amphibia-Reptilia* **28**, 418–426 (2007).

Fritz, U. *et al*. Mitochondrial diversity of European pond turtles (*Emys orbicularis*) in Anatolia and the Ponto-Caspian Region: multiple old refuges, hotspot of extant diversification and critically endangered endemics. *Org. Divers. Evol*. **9**, 100–114 (2009).

King, T. L. & Julian, S. E. Conservation of microsatellite DNA flanking sequence across 13 emydid genera assayed with novel bog turtle (*Glyptemys muhlenbergii*) loci. *Conserv. Genet.* **5**, 719–725 (2004).

Lenk, P., Fritz, U., Joger, U. & Wink, M. Mitochondrial phylogeography of the European pond turtle, *Emys orbicularis* (Linnaeus 1758). *Mol. Ecol.* **8**, 1911–1922 (1999).

Pedall, I., Fritz, U., Stuckas, H., Valdeón, A. & Wink, M. Gene flow across secondary contact zones of the *Emys orbicularis* complex in the Western Mediterranean and evidence for extinction and re-introduction of pond turtles on Corsica and Sardinia (Testudines: Emydidae). *J. Zool. Syst. Evol. Res.* **49**, 44–57 (2011).

Pedall, I., Schäfer, H., Fritz, U. & Wink, M. Isolation of microsatellite markers in the *Emys orbicularis* complex and development of multiplex PCR amplification. *Conserv. Genet.* **10**, 725–727 (2009).

Prusak, B. *et al.* Distribution of mitochondrial haplotypes (cyt *b*) in Polish populations of *Emys orbicularis* (L., 1758). *Biologia* **66**, 893–898 (2011).

Sommer, R. *et al*. Unexpected early extinction of the European pond turtle (*Emys orbicularis*) in Sweden and climatic impact on its Holocene range. *Mol. Ecol*. **18**, 1252–1262 (2009).

Stuckas, H. *et al.* Where are you from, stranger? The enigmatic biogeography of North African pond turtles (*Emys orbicularis*). *Org. Divers. Evol.* **14**, 295–306 (2014).

Vamberger, M. *et al.* Differences in gene flow in a twofold secondary contact zone of pond turtles in southern Italy (Testudines: Emydidae: *Emys orbicularis galloitalica, E. o. hellenica, E. trinacris*). *Zool. Scr.* **44**, 233–249 (2015).

Velo-Antón, G., Wink, M., Schneeweiß, N. & Fritz, U. Native or not? Tracing the origin of wild-caught and captive freshwater turtles in a threatened and widely distributed species (*Emys orbicularis*). *Conserv. Genet.* **12**, 583–588 (2011).

Velo-Antón, G., Pereira, P., Fahd, S., Teixeira, J. & Fritz, U. Out of Africa: did cross the Strait of Gibraltar twice? *Amphibia-Reptilia* **36**, 133–140 (2015).
